# The genetic basis of aneuploidy tolerance in wild yeast

**DOI:** 10.7554/eLife.52063

**Published:** 2020-01-07

**Authors:** James Hose, Leah E Escalante, Katie J Clowers, H Auguste Dutcher, DeElegant Robinson, Venera Bouriakov, Joshua J Coon, Evgenia Shishkova, Audrey P Gasch

**Affiliations:** 1 Center for Genomic Science InnovationUniversity of Wisconsin–MadisonMadisonUnited States; 2 Laboratory of GeneticsUniversity of Wisconsin-MadisonMadisonUnited States; 3 Great Lakes Bioenergy Research CenterMadisonUnited States; 4 Department of Biomolecular ChemistryUniversity of Wisconsin–MadisonMadisonUnited States; 5 Department of ChemistryUniversity of Wisconsin–MadisonMadisonUnited States; 6 Morgridge Institute for ResearchMadisonUnited States; New York University School of MedicineUnited States; Weill Cornell MedicineUnited States

**Keywords:** aneuploidy, natural variation, proteotoxicity, wild strains, *S. cerevisiae*

## Abstract

Aneuploidy is highly detrimental during development yet common in cancers and pathogenic fungi – what gives rise to differences in aneuploidy tolerance remains unclear. We previously showed that wild isolates of *Saccharomyces cerevisiae* tolerate chromosome amplification while laboratory strains used as a model for aneuploid syndromes do not. Here, we mapped the genetic basis to Ssd1, an RNA-binding translational regulator that is functional in wild aneuploids but defective in laboratory strain W303. Loss of *SSD1* recapitulates myriad aneuploidy signatures previously taken as eukaryotic responses. We show that aneuploidy tolerance is enabled via a role for Ssd1 in mitochondrial physiology, including binding and regulating nuclear-encoded mitochondrial mRNAs, coupled with a role in mitigating proteostasis stress. Recapitulating *ssd1Δ* defects with combinatorial drug treatment selectively blocked proliferation of wild-type aneuploids compared to euploids. Our work adds to elegant studies in the sensitized laboratory strain to present a mechanistic understanding of eukaryotic aneuploidy tolerance.

## Introduction

Aneuploidy, in which cells carry an abnormal number of one or more chromosomes, is highly detrimental during mammalian development, since amplification of most human chromosomes is inviable during embryogenesis. Imbalanced and especially elevated expression from altered chromosomes is thought to tax cellular proteostasis, both by producing too much protein and when stoichiometric imbalance of interacting proteins disrupts cooperative folding (Donnelly and Storchová, 2015; Oromendia and Amon, 2014; Pavelka and Rancati, 2013). Yet ≥90% of tumors are aneuploid with little detriment and even benefits to cells, and the degree of aneuploidy is associated with poorer patient prognosis (Holland and Cleveland, 2012; Targa and Rancati, 2018). Aneuploidy is also common in several fungal species including fungal pathogens. In fact, chromosome amplification represents a major route to drug resistance in pathogenic infections, when amplification of drug transporters and defense mechanisms promotes drug evasion (Wertheimer et al., 2016; Ni et al., 2013; Bennett et al., 2014). Why aneuploidy is benign or beneficial in some cells but highly deleterious in others is not understood.

The yeast *Saccharomyces cerevisiae* has been a formidable model to understand why chromosome amplification is toxic in the first place. Several studies characterized suites of aneuploid laboratory strains to understand the mechanisms of aneuploidy toxicity and the effects of chromosomal amplification. In a well-studied laboratory strain, chromosome amplification leads to reduced cell growth, metabolic alterations, altered cell-cycle progression in part through aberrant cyclin regulation, activation of a common transcriptome program regardless of the amplified chromosome, and signatures of protein aggregation and defects clearing misfolded peptides, referred to as proteostasis stress (Torres et al., 2007; Torres et al., 2010; Oromendia et al., 2012; Sheltzer et al., 2012; Thorburn et al., 2013; Dephoure et al., 2014; Dodgson et al., 2016; Brennan et al., 2019). Despite the deleterious effects reported in lab strains, chromosome amplification is beneficial in the right environment and provides a rapid route to phenotypic evolution (Rancati et al., 2008; Pavelka et al., 2010; Yona et al., 2012; Filteau et al., 2015; Fontanillas et al., 2010). This is consistent with the prevalence of chromosome amplification in fungal pathogens emerging after drug-treatment regimens (Ni et al., 2013; Selmecki et al., 2009; Selmecki, 2006).

Studies in laboratory strains have clearly generated important information on the causes and consequences of aneuploidy. However, we previously reported a striking difference among wild isolates: a substantial number of wild strains are naturally aneuploid, in some cases carrying extra copies of multiple chromosomes (Gasch et al., 2016; Hose et al., 2015). Recent large-scale sequencing efforts provide confirmatory evidence, reporting over 20% of sequenced strains as aneuploid, with each of the 16 yeast chromosomes represented across affected strains (Peter et al., 2018). In contrast to well-studied laboratory strain W303, naturally aneuploid yeast show only subtle growth defects, no detectable metabolic differences, and lack evidence of the canonical stress response (Gasch et al., 2016; Hose et al., 2015). The relative tolerance is not a result of adaptation: we showed that naturally euploid strains selected for chromosome amplification also showed relatively mild growth defects, and euploid derivatives of aneuploid isolates grew similarly to the aneuploid parent. Although some strains show variable karyotypes over time, picking up or losing chromosomes during division, chromosome amplification in other strains is generally stable (Gasch et al., 2016). Thus, many wild yeast strains tolerate chromosome amplification whereas W303 cannot.

Here, we mapped the genetic basis for this phenotypic difference, by crossing a naturally aneuploid strain isolated from oak soil, YPS1009 with extra copies of Chromosome XII (Chr12), to laboratory strain W303 carrying an extra copy of Chr12. Mapping and confirmatory genetics reveal that the basis for the difference in aneuploidy tolerance lies in *SSD1*, encoding an RNA binding protein known to be hypomorphic in W303. Our results point to combinatorial dysfunction in mitochondrial physiology and cytosolic protein homeostasis as the basis for aneuploidy toxicity in *ssd1-* strains, and in wild-type aneuploids with drug-induced defects. Integrating our results with past yeast and mammalian studies presents a holistic view of eukaryotic responses to chromosome amplification.

## Results

**Figure 1.**
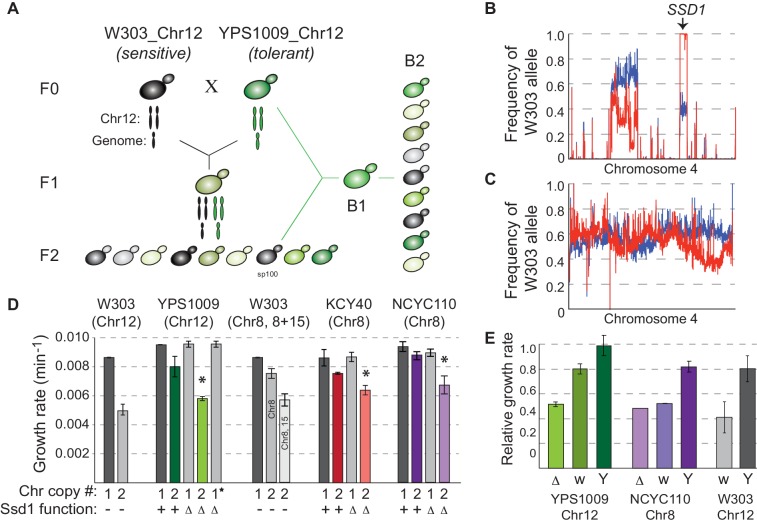
*SSD1* is required for aneuploidy tolerance. (**A**) Mapping schema, see Materials and methods. (**B**) W303 allele frequency across Chr4 in the pool of aneuploidy-sensitive (red) versus -tolerant (blue) B2 segregants or C) small (red) versus large (blue) colony pools from the euploid-control cross. (**D**) Average and standard deviation of growth rates for denoted strains with amplified chromosomes, indicated above. Number of chromosomes per haploid genome, *SSD1* status (Δ, deletion; –, *ssd1^W303^*), and star indicating euploid revertant are indicated below. Asterisk, p<0.005, T-test comparing aneuploids with and without *SSD1*. (**E**) Average and standard deviation of growth of aneuploid *ssd1-* strains harboring empty vector (Δ), *ssd1^W303^* (w) or *SSD1^YPS1009^* (Y), relative to the isogenic aneuploid wild type with empty vector (or euploid cells with empty vector in the case of W303 *ssd1^W303^* cells). Figure 1—source data 1.Source data for Figure 1.

To identify the genetic basis of differential aneuploidy tolerance, we crossed a haploid derivative of oak-soil strain YPS1009 disomic for chromosome 12 (YPS1009_Chr12) to W303 disomic for the same chromosome (W303_Chr12, Figure 1A). Haploid F2 segregants all harbor two copies of Chr12 but display quantitatively different growth rates (Figure 1—figure supplement 1). To score aneuploidy sensitivity, we focused on W303_Chr12 phenotypes, namely small colony size, slow growth, and/or propensity of the culture to lose the amplified chromosome during passaging. We realized during tetrad dissection that W303-inherited auxotrophies, especially adenine auxotrophy, influenced aneuploidy tolerance (Figure 1—figure supplement 2A-B). We therefore selected an F2 segregant prototrophic for influential markers (called ‘sp100’), backcrossed it to the tolerant YPS1009_Chr12 parent, and scored aneuploidy sensitivity as above (Figure 1A) to generate pools of aneuploidy-sensitive and aneuploidy-tolerant segregants (Figure 1—figure supplement 2C-D). To control for other genetic influences on growth rate and/or colony size, we also performed a control cross of the euploid parents, generating pools of euploid segregants with small versus large colony sizes (see Materials and methods).

**Figure 1—figure supplement 1.**
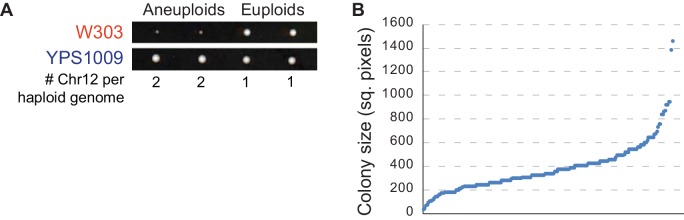
Aneuploidy tolerance varies in W303 and YPS1009 strains. (**A**). Tetrads resulting from dissection of diploid W303_Chr12-3n or YPS1009_Chr12-3n, each trisomic for Chr12, onto solid YPD medium. Aneuploidy segregates 2:2 (confirmed by qPCR as outlined in Materials and methods), with extreme colony size differences evident in aneuploid versus euploid W303 (top) but not YPS1009 (bottom). (**B**) Colony sizes of haploid, Chr12 aneuploid F2 segregants from the hYPS1009_Chr12 x W303_Chr12 cross. Cells are sorted by colony size.

**Figure 1—figure supplement 2.**
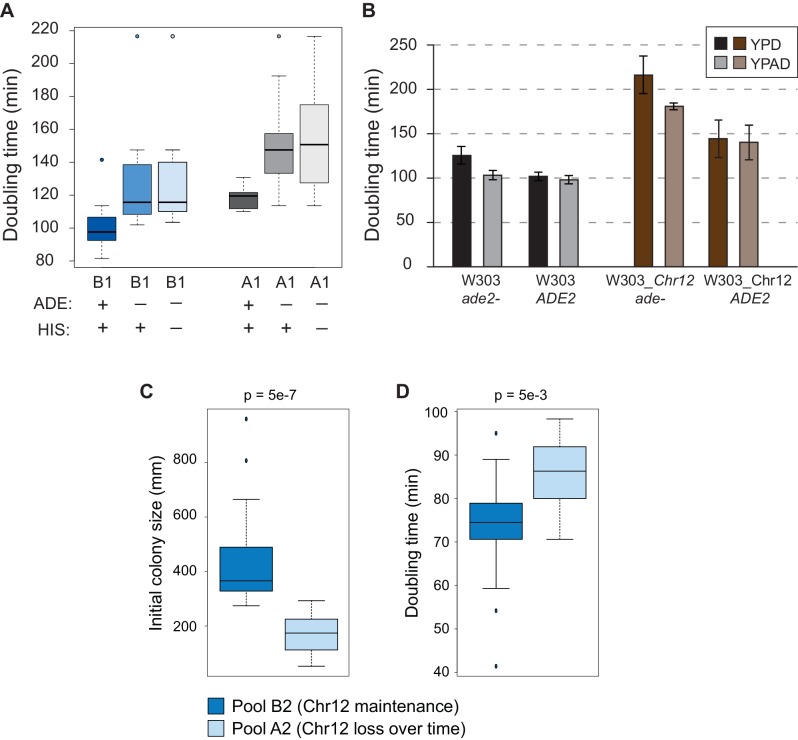
Auxotrophies influence aneuploidy tolerance. W303 harbors multiple auxotrophies that likely influence cell growth and potentially aneuploidy tolerance. We therefore phenotyped aneuploid F2s from the cross for auxotrophies including adenine (ade-), histidine (his-), uracil, leucine and tryptophan, by plating cells on synthetic medium lacking each nutrient. (**A**) Doubling-time distributions of aneuploidy-tolerant (B1 pool) and aneuploidy-sensitive (A1 pool) F2s in liquid YPD medium. Pools were defined based on propensity of each culture to lose Chr12 after ~20 generations of growth. Auxotrophies were determined at the time of tetrad dissection by replica plating onto drop-out media. Ade- cells in Pool B1 that maintained Chr12 after passaging showed slower growth in rich medium compare to Ade+ cells, with little additional effect if cells were also auxotrophic for histidine (his-), whereas the aneuploidy sensitive pool showed a wider range of growth rates. (**B**) To test if adenine auxotrophy influences the trait, we supplemented adenine to the media or reintroduced the missing ADE2 gene. Figure 1—figure supplement 2B shows the average and standard deviation of doubling times for euploid (gray scale) and aneuploid (brown scale) W303 with or without the *ADE2* gene integrated into the native genomic locus, as cells were grown in rich medium (YPD) or rich medium supplemented with additional adenine (YPAD). Reintroducing the *ADE2* gene into euploid W303 improved growth (*i.e.* decreased doubling time) to the same effect as adenine supplementation. However, reintroducing *ADE2* in the W303_Chr12 aneuploid produced a much more dramatic effect than supplementation, decreasing doubling time by nearly 30%. Thus, adenine prototrophy specifically improves the growth rate of aneuploid W303. Propensity of the culture to lose Chr12 is highly correlated with growth rate in backcrossed spores. Spores from the backcross (see Figure 1A) were partitioned into aneuploidy-tolerant pool B2 and aneuploidy-sensitive pool A2 based only on the propensity of the culture to lose Chr12 after passaging. This phenotype was highly correlated with initial colony size after dissection (**C**) and doubling time (**D**), even though colony size and growth rate were not considered in the pooling. In both cases, the aneuploidy-sensitive strains showed considerably reduced growth (p-value above plots, Welch’s T-test).

**Figure 1—figure supplement 3.**
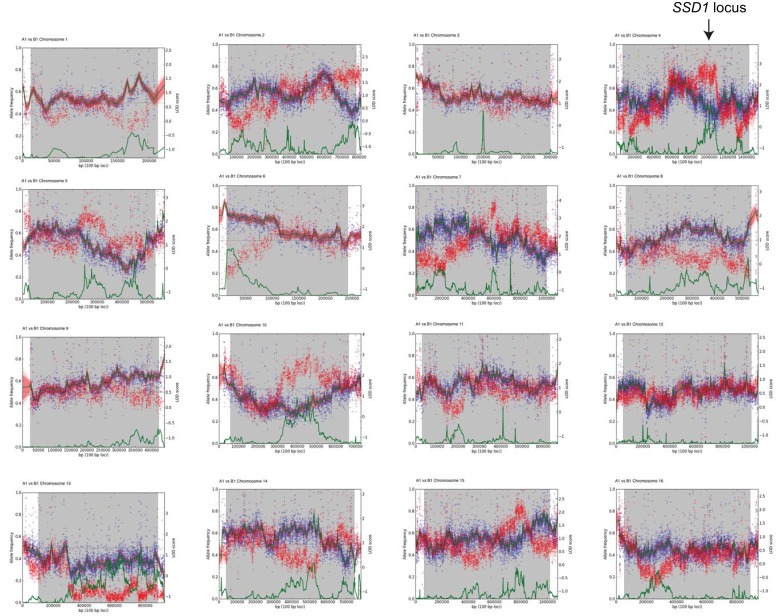
Multipool output for initial cross. As shown in Figure 1B but for each of the 16 yeast chromosomes from the initial cross.

**Figure 1—figure supplement 4.**
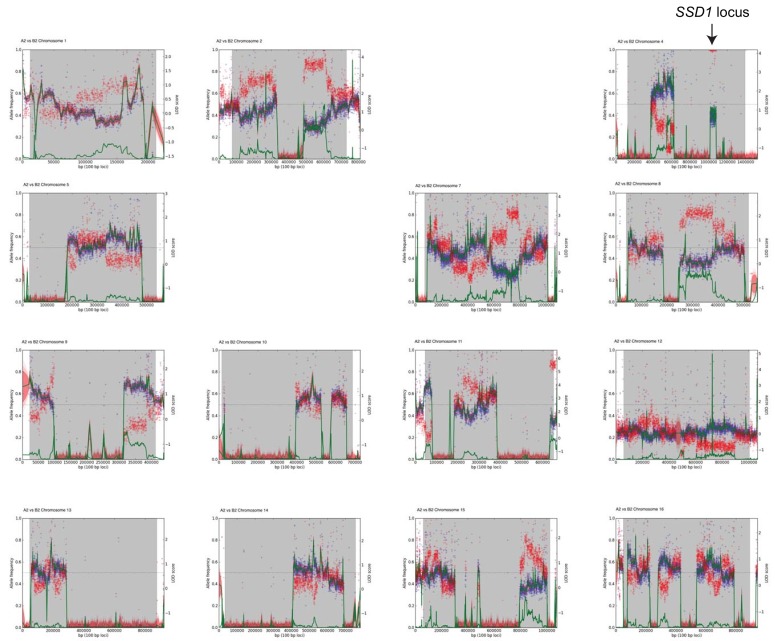
Multipool output for the backcross. As shown in Figure 1B but for each of the 16 yeast chromosomes from the YPS1009_Chr12 backcross. LOD traces are shown in green. Missing plots for Chr three and Chr six result from failed Multipool runs, presumably due to low recombination rates in the backcross. We focused on peaks enriched for W303 alleles in both the initial cross (Figure 1—figure supplement 3) and backcross that were not significant in the euploid cross scoring growth rate.

**Figure 1—figure supplement 5.**
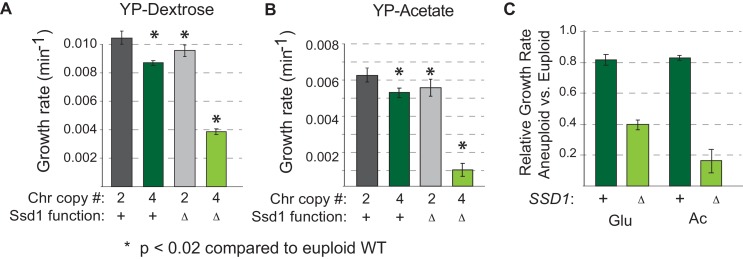
Ssd1 is required for aneuploidy tolerance in diploid YPS1009. As shown in Figure 1D. for diploid YPS1009 with two or four copies of Chr12, with and without *SSD1* according to the key, for cells grown on A) dextrose or B) acetate as a sole carbon source. (**C**) Growth rates in each aneuploid relative to the paired euploid is shown for direct comparison to haploid strains in Figure 4A. The relative growth rate of diploid cells tetrasomic for Chr12 was more severely affected by *SSD1* deletion, especially on acetate where cells grew extremely slowly. The latter result is consistent with our published results in which we were unable to make diploid W303 aneuploids that could maintain respiration, whereas haploid W303 with extra chromosomes was defective but showed some respiratory capability (Hose et al., 2015). Interestingly, unlike in haploids, the diploid YPS1009 euploid strain showed a subtle but statistically significant (*=p < 0.02, paired T-test) growth reduction compared to wild-type, euploid cells, implicating Ssd1 in base-ploidy specific effects.

Bulk analysis of aneuploidy-sensitive and -tolerant backcrossed segregants revealed a major-effect locus on Chr4 that was nearly fixed for W303 alleles in the aneuploidy-sensitive pool (Figure 1B and Figure 1—figure supplements 3, 4) but not small colonies from a euploid-control cross (Figure 1C, see Materials and methods). The locus spanned *SSD1*, encoding an RNA-binding protein. This locus harbors a premature stop codon in W303 that deletes 44% of the Ssd1 protein including conserved RNA binding domains (Uesono et al., 1997; Sutton et al., 1991). Ssd1 is best characterized for regulating localization and translation of cell-wall destined mRNAs, delivered by Ssd1 to the growing bud during active growth but to P-bodies for translational silencing following stress or mitotic defects (Jansen et al., 2009; Kurischko et al., 2011a). Ssd1 has also been implicated in a large number of suppressor screens and has a role in aging and quiescence (Miles et al., 2019; Li et al., 2013; Hu et al., 2018). W303 carries a premature stop codon that ablates RNA binding domains, which underlies several phenotypic differences reported between W303 and other strains (Kaeberlein et al., 2004; Moriya and Isono, 1999; Ohyama et al., 2010; Uesono et al., 1994).

Genetic analysis confirmed that *SSD1* underlies the difference in aneuploidy tolerance. *SSD1* deletion had little effect on the growth of euploid YPS1009 but significantly retarded YPS1009_Chr12 proliferation, comparable to W303_Chr12 (Figure 1D). The phenotype was true in both haploid and diploid versions of the strain (Figure 1—figure supplement 5). Growth rate was restored if cells lost the extra chromosome during passaging (Figure 1D, star) or if *SSD1^YPS1009^* was reintroduced (Figure 1E). To test if Ssd1’s role was exclusive to this genetic background or chromosome amplification, we deleted *SSD1* in naturally aneuploid, diploid West African strain tetrasomic for Chr 8 (NCYC110_Chr8) and in a derived aneuploid vineyard strain, KCY40_Chr8 (Hose et al., 2015). *SSD1* deletion sensitized cells to chromosome amplification, showing that the effect is independent of genetic background and duplicated chromosome (and is thus also independent of the rDNA locus on Chr 12) (Figure 1D). Reintroducing the YPS1009 allele of *SSD1* complemented the aneuploidy sensitivity of multiple strain backgrounds (Figure 1E), whereas the W303 allele provided no complementation in the NCYC110_Chr8 *ssd1Δ* strain and partial complementation in YPS1009_Chr12 *ssd1Δ* (Figure 1D). Importantly, expressing the YPS1009 allele in W303_Chr12 largely corrected its sensitivity (with a remaining contribution likely from the adenine auxotrophy, see Figure 1—figure supplement 2B), demonstrating that the *ssd1^W303^* allele is responsible for aneuploidy sensitivity in W303. Thus, Ssd1 plays a generalizable role in tolerating chromosome amplification across multiple strains and chromosome duplications.

### Loss of Ssd1 recapitulates multiple signatures of aneuploid W303

**Figure 2.**
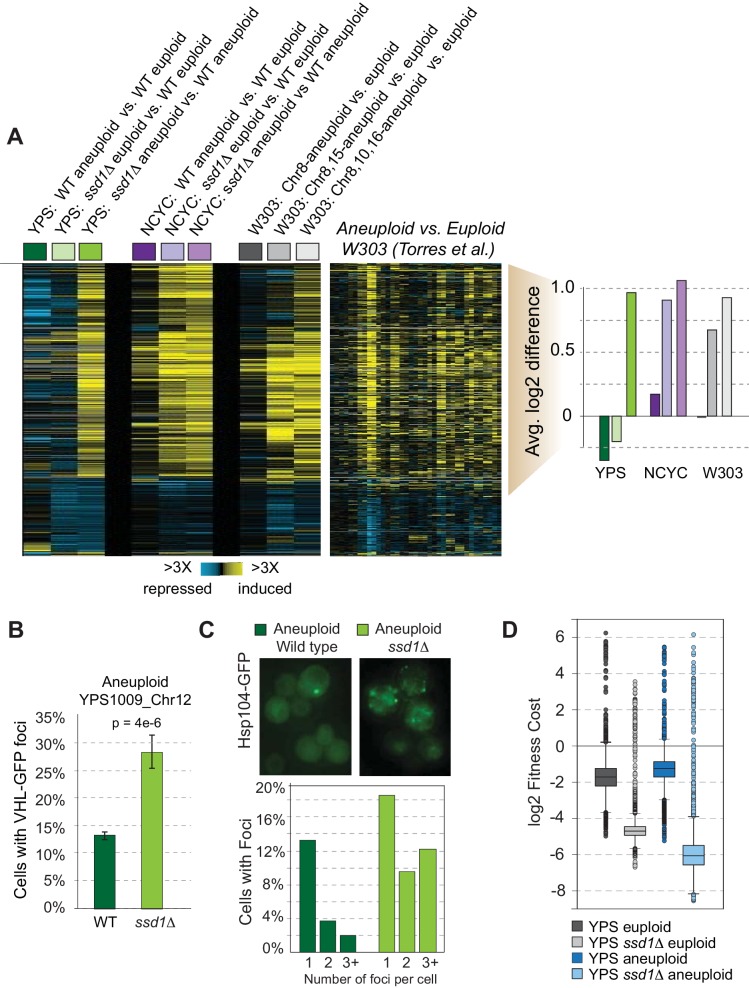
*SSD1* deletion induces aneuploidy signatures. (**A**) Replicate-averaged log_2_ expression differences for strain comparisons (columns) across 861 genes (rows) differentially expressed in mutant versus wild-type aneuploids, see text. Strains include haploid YPS1009 disomic for Chr12, diploid NCYC110 tetrasomic for Chr 8, or haploid W303 derivatives with different chromosome amplifications. Corresponding data from Torres et al. and average log_2_ differences in expression of induced genes are shown, where colors indicate strain labels from left. (**B–C**) Quantification of B) VHL-GFP foci and C) Hsp104-GFP in aneuploid strains. Data represent average and standard error of the mean (SEM) across biological triplicates, p from Fisher’s exact test. (**D**) Distribution of replicate-averaged fitness costs from high-copy plasmid over-expression in each strain (see Materials and methods). Figure 2—source data 1.Transcriptome data shown in Figure 2A.

W303 studies reported a transcriptomic signature of aneuploidy independent of amplified chromosome identity, but this is not seen in wild aneuploid strains (Torres et al., 2007; Hose et al., 2015). To test dependence on Ssd1, we followed transcriptomes of natural aneuploids and their *ssd1Δ* derivatives, with or without extra chromosomes. We identified 861 genes with altered expression in both YPS1009_Chr12 *ssd1Δ* and NCYC110_Chr8 *ssd1Δ* mutants compared to their isogenic wild-type aneuploids (false discovery rate, FDR < 0.05, Figure 2A). Induced genes showed little change in euploid YPS1009 *ssd1Δ* but were up-regulated when *SSD1* was deleted in the context of Chr12 amplification. NCYC110 showed similar trends, except that in this strain we observed a weak expression signature in euploid *ssd1Δ* cells that was exacerbated when Chr8 was amplified. Repressed genes included rRNA and tRNA processing and cytokinesis factors, whereas induced genes encompassed the environmental stress response (ESR Gasch et al., 2000), oxidoreductases, carbohydrate and energy metabolism, and genes involved in mitochondrial degradation (p<1e-4, hypergeometric test). This response effectively recapitulates the expression signature seen in W303 aneuploids (Torres et al., 2007, Figure 2A). The response was exacerbated with increasing DNA content in W303 carrying multiple extra chromosomes (Figure 2A). Thus, the previously reported aneuploidy transcriptome signature results from defective Ssd1 function, independent of affected chromosome, exacerbated with additional DNA content, and with some strain-specific nuances.

**Figure 2—figure supplement 1.**
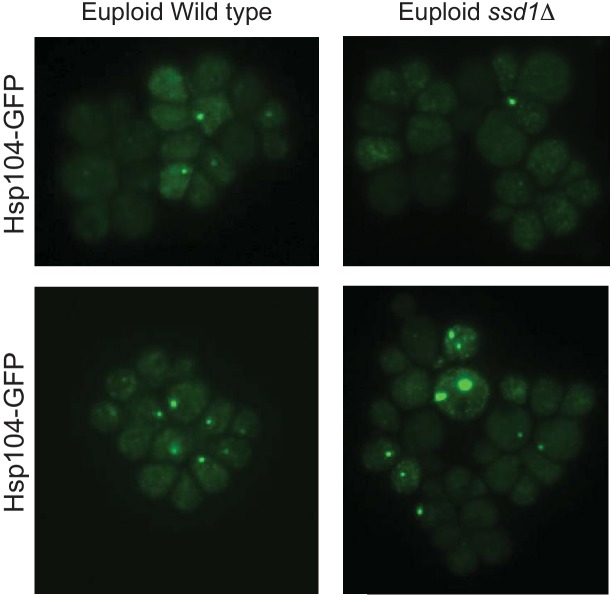
Hsp104-GFP foci in euploid cells. Representative images of euploid YPS1009 wild type and *ssd1Δ* cells. For the most part, the euploid mutant looked very similar to the wild-type strain in terms of Hsp104-GFP foci number and quality, with only occasional cells harboring >1 Hsp104-GFP focus (bottom example). Thus, defects in proteostasis are occasionally seen in the euploid mutant but exacerbated by Chr12 duplication in YPS1009_Chr12 *ssd1Δ* cells.

We wondered if other aneuploidy signatures seen in W303 could be explained by defective Ssd1. In addition to growth delay, aneuploid W303 strains reportedly exhibit larger cell size, altered cyclin Cln2 abundance, delayed G1/S progression, metabolic defects, and signatures of proteotoxicity including protein aggregation and a defect degrading misfolded protein (Torres et al., 2007; Oromendia et al., 2012; Thorburn et al., 2013). While these seminal studies generated important information on aneuploidy toxicity in a sensitized strain, many phenotypes likely result from defective Ssd1. YPS1009_Chr12 *ssd1Δ* grows slower (Figure 1), produces ~33% higher optical versus cell density indicating larger size, and displays metabolic defects encompassing defective respiratory growth (see Figure 4). We confirmed that Ssd1 binds many cell-cycle transcripts including *CLN2* (Supplementary file 2), which is translationally regulated by Ssd1 and explains altered G1/S progression (Ohyama et al., 2010).

Loss of *SSD1* also explains proteotoxicity observed for aneuploid strains. First, we followed accumulation of human Von Hippel Lindau (VHL) protein upon over-expression. Because VHL cannot fold in the absence of its interacting proteins in yeast, accumulation of VHL-GFP foci reflects misfolded protein that has yet to be cleared by the proteasome (McClellan et al., 2005; Kaganovich et al., 2008). We found that significantly more YPS1009_Chr12 *ssd1Δ* cells accumulated VHL-GFP foci compared to wild type (Figure 2B). We also followed the protein disaggregase Hsp104, which binds misfolded and aggregated proteins in discrete protein quality-control centers (Kaganovich et al., 2008; Chernova et al., 2017). Wild-type aneuploids showed no obvious difference in the number of Hsp104-GFP foci compared to euploids (Figure 2C and Figure 2—figure supplement 1), indicating that gross protein aggregation is not a universal feature of aneuploid yeast. However, mutant aneuploids lacking *SSD1* showed a higher proportion of cells with Hsp104-GFP foci, and more foci within those cells, compared to wild-type aneuploids (Figure 2C). Ssd1 was previously implicated in protein homeostasis after heat shock (Mir et al., 2009), but our results demonstrate that chromosome amplification in the absence of other stresses is enough to provoke misfolding in *ssd1Δ* cells. Together, these results show that myriad signatures of W303 aneuploidy can occur due to defective Ssd1.

Ssd1 mutants could be sensitive to specific genes on the amplified chromosomes, or they could have a generalized sensitivity to the burden of extra DNA/protein. To distinguish these models, we transformed YPS1009 strains with a barcoded, high-copy gene over-expression library and measured relative fitness costs after 5 generations of growth (see Materials and methods). Both the euploid and aneuploid *ssd1Δ* mutants were highly sensitive to the library (Figure 2D): genes that were deleterious in wild type were toxic in the mutant, while many genes with neutral effect in parental strains were deleterious in the absence of *SSD1*. We cannot exclude a defect maintaining the high-copy 2-micron plasmid (indeed, the mutant cells do not grow well with the empty vector, and Ssd1 is already implicated in plasmid maintenance Uesono et al., 1994). Nonetheless, both the euploid and aneuploid *ssd1Δ* mutants are highly sensitive to the 2-micron overexpression library.

### Ssd1 binds RNAs and alters aneuploid proteomes

We focused on YPS1009 strains to elucidate Ssd1 function in aneuploidy tolerance. Revisiting the YPS1009_Chr12 *ssd1Δ* transcriptome revealed broader induction of genes, including mRNAs whose proteins localize to subcellular compartments such as mitochondria, ER, vacuole, peroxisome, plasma membrane, and the cell wall (p<1e-4, hypergeometric test). Many of these organelles functionally and physical interact (Scorrano et al., 2019), raising the possibility of broader inter-organelle issues. Consistent with this notion, the *ssd1Δ* aneuploid also showed transcriptional signatures of the unfolded ER-protein response (Travers et al., 2000) and mitochondrial protein import stress (Weidberg and Amon, 2018) (see Materials and methods).

To test if Ssd1 binds a broader set of mRNAs, we sequenced RNAs recovered from Ssd1 immunoprecipitation (see Materials and methods). The 286 associated mRNAs (FDR < 0.05, Supplementary file 2) were heavily enriched for transcripts encoding cell-wall proteins as expected, but the group was also enriched for cell-cycle regulated mRNAs (including cyclins *CLN2* and *CLB2/4/5*) and those involved in budding, RNA metabolism, and sterol transport (p<1e-4). Myriad other functions were also represented, such as proteins in chromatin regulation, transcription, lipid biogenesis, endocytosis, protein homeostasis, and mitochondrial function, some previously noted (Jansen et al., 2009; Hogan et al., 2008). Interestingly, Ssd1 also bound mRNAs encoding osmotic-response regulators (*SLN1, SHO1, MSB2, HOT1*), notable since osmotic stress was recently implicated in aneuploidy responses of a different lab strain (Tsai et al., 2019). Ssd1-bound mRNAs were not enriched for those encoded on the amplified chromosome, nor transcripts disproportionately expressed compared to DNA content (Hose et al., 2015), suggesting that the mechanism of aneuploidy tolerance is not through modulation of amplified-gene expression. Most (60%) bound transcripts were not differentially expressed in the aneuploid mutant, downplaying a generalizable role in regulating mRNA abundance. In turn, most mRNAs differentially expressed in the aneuploid mutant are not Ssd1-bound, suggesting widespread secondary responses to the primary defect(s).

**Figure 3.**
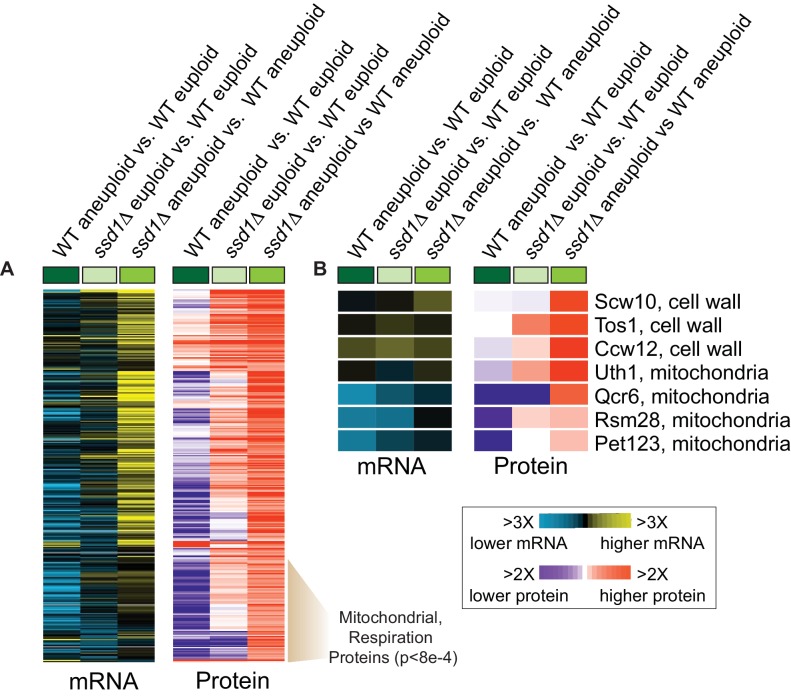
Ssd1 affects the proteome in aneuploid YPS1009 cells. (**A**) Replicate-averaged log2(fold difference) in abundance across 301 significant proteins (FDR < 0.05) and their corresponding mRNAs (rows) for denoted comparisons (columns), where colors represent the magnitude of change according to the key. The indicated cluster is enriched for mitochondrial proteins and respiration factors (hypergeometric test). (**B**) Representative Ssd1-bound transcripts from (**A**). Figure 3—source data 1.source data for Figure 3.

Since Ssd1 regulates translation via direct RNA binding (Jansen et al., 2009; Wanless et al., 2014; Kurischko et al., 2011b), we next used quantitative proteomics to measure effects on the cellular proteome. 301 of 3906 measured proteins were more abundant in the aneuploid mutant versus wild type (FDR < 0.05, Figure 3A). Many emerge from induced transcripts; however, a large fraction of proteins was elevated beyond mRNA differences, including cell-wall proteins, nuclear-encoded mitochondrial proteins (p=6e-4, hypergeometric test), and others. Many of these proteins were also elevated in the euploid mutant without significant mRNA changes (Figure 3A). Although there was no enrichment for proteins encoded by Ssd1-bound transcripts, several elevated proteins emerge from Ssd1 targets, including cell-wall transcripts and several nuclear transcripts encoding mitochondrial proteins. For example, *UTH1* encoding a mitochondrial protein linked to aging is bound by Ssd1 in our and other studies and is known to be translationally regulated by direct Ssd1 binding at specific mRNA locations (Wanless et al., 2014; Camougrand et al., 2004; Camougrand et al., 2000). Although protein induction was evident in euploid *ssd1Δ* cells, the defect was exacerbated by Chr12 amplification, with ~4X more Uth1 protein despite little difference in mRNA (Figure 3B). Other mitochondrial proteins emanating from Ssd1-bound mRNAs were also significantly elevated in the *ssd1Δ* aneuploid, including several mitochondrial ribosomal proteins (Figure 3B). Thus, Ssd1 affects the proteome of aneuploid cells, including from bound transcripts.

### Ssd1 is important for mitochondrial function and inheritance

**Figure 4.**
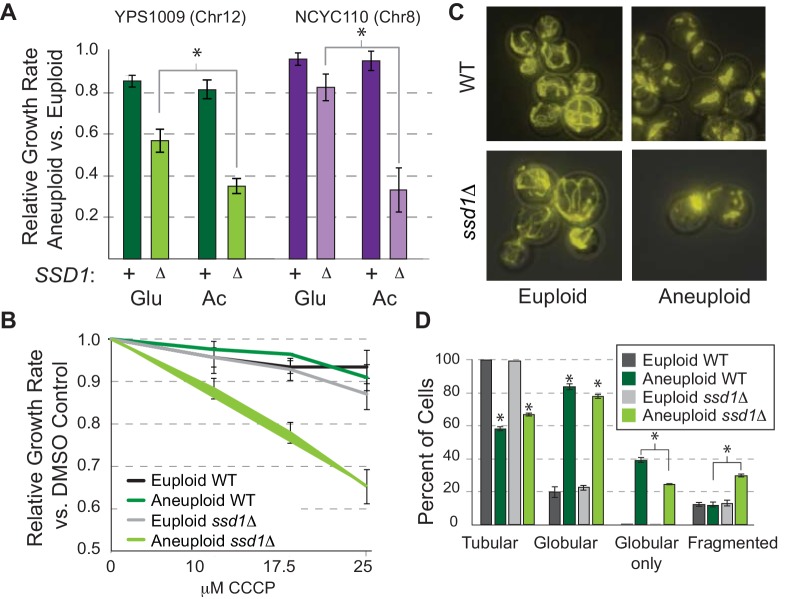
Ssd1 affects mitochondrial function and morphology. (**A**) Average and standard deviation of growth rates for denoted aneuploids versus euploids ± *SSD1* in glucose (Glu) or acetate (Ac). Asterisk, p<2e-4, replicate-paired T-test. (**B**) Average growth rates across CCCP doses. (**C**) Representative images of rhodamine-B stained mitochondria and D) quantified morphologies for cells with any tubular, any globular, only globular, or fragmented mitochondria (average and SEM, see Materials and methods). p<0.0001, Fisher’s exact test.

Our past work revealed that wild aneuploid strains down-regulate many nuclear encoded mitochondrial transcripts, a response also seen in Down syndrome models (Helguera et al., 2013; Liu et al., 2017), hinting that mitochondrial regulation is important for tolerating chromosome amplification (Hose et al., 2015). In the current work, multiple lines implicated mitochondrial effects in *ssd1Δ* aneuploids. To explore this, we tested mitochondrial function in YPS1009 strains. We found a synergistic defect between *SSD1* deletion and aneuploidy when cells experienced mitochondrial stress. YPS1009_Chr12 *ssd1Δ*, NCYC110_Chr8 *ssd1Δ* (Figure 4A), and aneuploid W303 (Hose et al., 2015) were all sensitive to non-fermentable acetate, beyond what is expected from the additive effects of aneuploidy and reduced respiratory growth rate. This demonstrates Ssd1-dependent respiratory dysfunction autonomous of strain background or affected chromosome. YPS1009_Chr12 *ssd1Δ* cells were also susceptible to sub-lethal doses of carbonyl-cyanide 3-chlorophenylhydrazone (CCCP), which uncouples mitochondrial membrane potential (Figure 4B). Notably, aneuploid *ssd1Δ* cells were no more sensitive than expected to cell wall or ER stress (Figure 4—figure supplement 1), indicating a specific interaction with mitochondrial challenge. In the process of this work, we discovered that *ssd1Δ* aneuploid cells also showed a striking difference in mitochondrial morphology. Wild-type YPS1009_Chr12 grew well but harbored many globular mitochondria compared to the euploid tubular shape (Figure 4C). Although the impact of this morphology is not clear, YPS1009_Chr12 *ssd1Δ* displayed significant differences, including more tubular forms and increased mitochondria fragmentation (Figure 4D). The West African aneuploid did not display globular mitochondria but did display dysfunction (Figure 4A). Thus, *ssd1Δ* cells show numerous signs of mitochondrial dysfunction compared to wild-type aneuploid cells.

**Figure 4—figure supplement 1.**
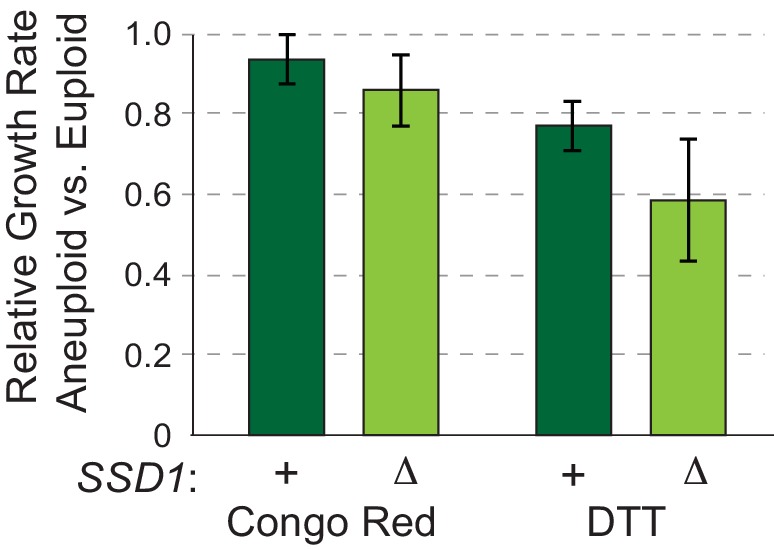
No synergistic defects between aneuploidy and cell-wall or ER stress. Average and standard deviation of growth rates in aneuploid relative to euploid cells in the presence of 0.02 mg/mL Congo red or 2.5 mM dithiolthreitol (DTT). As shown in Figure 4A. Aneuploid YPS1009_Chr12 *ssd1Δ* cells are not significantly more sensitive than wild-type cells to cell-wall stress inflicted by Congo red or ER stress via DTT (p>0.05, replicate-paired T-test). The lack of synergistic sensitivity to these drugs indicates that the synergistic sensitivity of the mutant to aneuploidy plus mitochondrial stress, non-fermentable acetate, and NTC (see main text) is not due to overall stress sensitivity of the aneuploid mutant but rather implicates a specific defect in mitochondrial function and proteostasis in aneuploid *ssd1Δ* cells.

**Figure 5.**
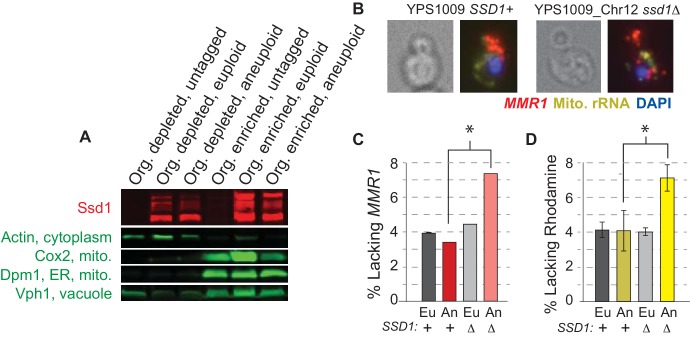
Ssd1 affects mRNA localization. (**A**) Representative Western blot showing Ssd1-GFP and markers of mitochondria, ER, and vacuole as detected in organelle-depleted and organelle-enriched fractions from *SSD1-GFP* or untagged-Ssd1 strains (see Methods). Ssd1 fragments migrating below the expected top band emerge during the fractionation incubations. (**B**) Representative smFISH showing bud-localized M*MR1* transcript in wild-type and *ssd1Δ* aneuploids. (**C**) Quantification of percent buds lacking *MMR1* from smFISH (see Materials and methods). (**D**) Percent of cells lacking Rhodamine staining. Histograms represent average and SEM across biological triplicates; *, p<0.02, Fisher’s exact test.

We wondered if Ssd1 plays a direct role in mitochondrial function, perhaps by localizing nuclear-encoded mitochondrial mRNAs as it does cell-wall mRNAs. We first scored Ssd1 localization by centrifugation-based cellular fractionation. Ssd1 was reproducibly recovered in the organelle-enriched fraction that was depleted of cytosolic actin but enriched for markers of mitochondria (Cox2), ER (Dpm1), and vacuole (Vph1, Figure 5A), which themselves interact. Attempts to separate the components by immunoprecipitation of Ssd1 from the fractions were not successful. We next followed cellular localization of *MMR1* transcript, encoding a bud-mitochondria localized protein involved in mitochondrial inheritance (Itoh et al., 2004), by single-molecule RNA FISH (smFISH, Figure 5B). In most wild-type cells, mother-encoded *MMR1* was directed to the nascent bud, before the nucleus migrated. Although scoring precise differences in mRNA patterns was challenging, YPS1009_Chr12 *ssd1Δ* cells displayed twice as many buds lacking *MMR1* as wild-type aneuploids (Figure 5C, p<0.02, Fisher’s exact test). In addition, the mutant showed double the cells lacking mitochondria as indicated by Rhodamine staining (Figure 5D), consistent with a defect in mitochondrial inheritance. It is possible that the mutant suffers from delayed dynamics, rather than fully aberrant localization in individual cells. Other nuclear-encoded mitochondrial mRNAs bound by Ssd1 were too abundant to follow by smFISH and will require further delineation. Nonetheless, together our work shows that Ssd1 associates with organelle fractions that include mitochondria (Figure 5A), binds several nuclear-encoded mitochondrial transcripts (Supplementary file 2), and can influence abundance of mitochondrial proteins (Figure 3B) or localization patterns of bound transcript (Figure 5B–C), consistent with the requirement of *SSD1* for proper mitochondrial function in aneuploid cells (Figure 4).

### Combinatorial mitochondrial dysfunction and proteostasis stress underlie aneuploidy sensitivity in *ssd1Δ* cells

A remaining question is why Ssd1 dependence and mitochondrial dysfunction are more severe in aneuploids. We reasoned that underlying *ssd1Δ* defects are exacerbated by effects of chromosome amplification. One candidate is proteome stress that may emerge from over-production of proteins from the amplified chromosome in the absence of Ssd1-dependent translational silencing, which could tax the proteostatic buffering capacity specifically in the mutant (Donnelly and Storchová, 2015; Oromendia and Amon, 2014; Oromendia et al., 2012). Many recent studies have revealed a connection between mitochondrial function and cytosolic proteome stress: defects in mitochondrial protein import induce cytosolic proteostatic defense mechanisms, and misfolded cytosolic proteins interact with and can even be cleared by mitochondria (Qureshi et al., 2017; Ruan et al., 2017; Wrobel et al., 2015). Furthermore, mitochondrial defects and cytosolic proteostasis stress co-emerge in neurological syndromes, aging, and aneuploidy (Oromendia and Amon, 2014; Helguera et al., 2013; Ruan et al., 2017; Franco-Iborra et al., 2018; Kauppila et al., 2017).

**Figure 6.**
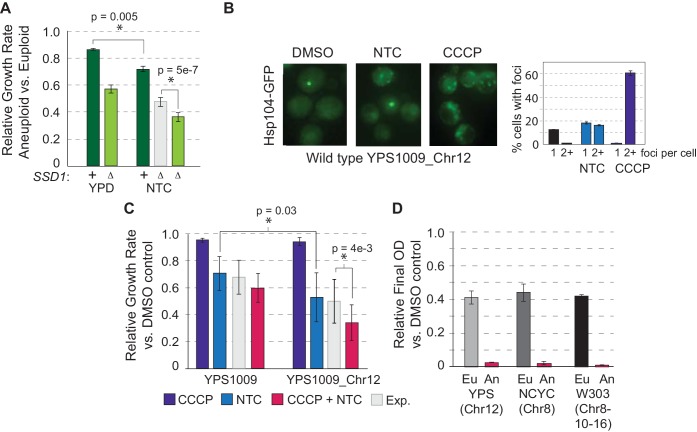
Protein misfolding and mitochondrial dysfunction sensitize aneuploids. (**A**) Average and standard deviation of relative growth rates in rich YPD medium or with 1 ug/mL NTC. The expected (Exp, light gray) additive effect was calculated based on the fold-drop in growth rate of NTC-treated euploid cells applied to the wild-type aneuploid growth rate in the absence of NTC. (**B**) Representative Hsp104-GFP foci triggered by 1 ug/mL NTC or 25 uM CCCP and quantification in YPS1009_Chr12 (average and SEM). (**C**) Average relative growth rate over three generations for indicated treatments or additive expectation (Exp, paired T-test). (**D**) Relative final optical density after overnight CCCP + NTC treatment in euploid (Eu) and aneuploid (An) strains (see Materials and methods). Figure 6—source data 1.Clustal Omega alignment of YPS1009 and seven other strains with truncated alleles.Fasta files were recapitulated by mapping SNPs from the published vcf file^8^ onto the S288c sequence and performing a multiple alignment using Clustal Omega (https://www.ebi.ac.uk/Tools/msa/clustalo/). Allelic differences are highlighted in yellow below. Only regions with polymorphisms are shown.

To test the model that synergistic dysfunction underlies aneuploidy sensitivity in *ssd1Δ* aneuploid yeast, we applied nourseothricin (NTC), among the aminoglycoside drugs that induce mistranslation and protein misfolding (Ling et al., 2012). We confirmed that NTC treatment increased the number of Hsp104-GFP foci in the aneuploid wild type, and discovered that CCCP produced an even stronger effect even though wild-type aneuploids grew well in the drug (Figures 6 and 4B). Wild-type aneuploids were slightly sensitive to NTC, but the mutant was significantly more sensitive, beyond the expected additivity of aneuploidy and NTC response, revealing a synergistic defect induced by the drug in combination with aneuploidy (Figure 6A). The NTC sensitivity suggests that wild-type aneuploids with full length *SSD1* can largely buffer proteostasis upon chromosome amplification but may exist near capacity. Mitochondrial defect, protein over-abundance, and mislocalized transcripts/proteins resulting from *SSD1* deletion may simply push cells over the edge (see Discussion).

This raised an important prediction: if synergistic defects in mitochondrial function and proteostasis sensitize *ssd1Δ* cells to chromosome amplification, then combinatorial drug treatment to mimic these defects should selectively target wild-type aneuploids. In fact, this was the case: wild-type euploid and aneuploid cells tolerated short-term CCCP and NTC individually, but when combined aneuploid growth was significantly delayed beyond the euploid strain and the expectation of additive effects (Figure 6C). Longer-term combinatorial drug treatment limited growth of euploid YPS1009 but selectively blocked proliferation in YPS1009_Chr12 (Figure 6D). The effect was persistent across strains and chromosome amplifications: combinatorial treatment halted over-night growth of W303 with duplications of Chr8, Chr10, and Chr16 and NCYC110 carrying extra Chr 8 (although in this strain CCCP was actually protective against NTC toxicity in the euploid cells at the doses used)(Figure 6D).

## Discussion

Our work has several major implications for understanding the consequences of aneuploidy and how to modulate them. First, we resolve the discrepancy in the literature between wild and laboratory-strain responses to aneuploidy, by showing that mutation of a single gene explains the phenotypic difference among strains studied here. Many of the yeast phenotypes previously reported as signatures of aneuploidy, including proteostasis defects, metabolic defects, cell-cycle defects, and transcriptome response, can be caused by *SSD1* deletion or mutation as seen in the commonly used W303 lab strain. This result explains why wild yeast from our studies, other studied *S. cerevisiae* strains, and pathogenic fungi do not show major defects upon chromosome amplification – these fungi have mechanisms to tolerate extra chromosomes. Our results underscore the importance of studying multiple strain backgrounds to understand model organism biology. At the same time, although W303 is clearly a sensitized strain we highlight that many important insights have come from its dissection. We propose that integrating our results with past yeast and mammalian studies reconciles to a holistic view of eukaryotic aneuploidy physiology.

Our model posits that Ssd1’s function in translational silencing and mitochondrial physiology enable aneuploidy tolerance in wild yeast. The wild aneuploid strains studied here do not show signs of metabolic or proteostatic stress under standard growth conditions. But mimicking *ssd1Δ* defects through combinatorial drug treatment sensitizes cells to extra chromosomes, showing that it is indeed combined dysfunction in mitochondria and proteome management that is responsible for aneuploidy sensitivity. We propose that, under normal conditions, wild aneuploids handle the extra chromosome by buffering the effects of gene/protein amplification – yet cells may exist close to their proteostatic buffering capacity. Additional stress on the proteostasis system, due to drugs or *SSD1* deletion, pushes cells beyond capacity, thereby limiting fitness.

How does Ssd1 fulfill this function? Ssd1 has a clear role in translational regulation: it localizes to P-bodies during times of stress, suppresses encoded protein abundance via direct RNA binding, and is linked to the reduction of polysomes in aged cells (Jansen et al., 2009; Kurischko et al., 2011a; Hu et al., 2018; Wanless et al., 2014; Kurischko et al., 2011b). Ssd1’s role in mitigating proteostatic stress likely emerges via RNA binding, since the *ssd1^W303^* allele lacks the carboxyl-terminal RNA binding domain (Uesono et al., 1997). A remaining question is if mRNAs bound by Ssd1 are especially relevant to proteome homeostasis. Brennan et al. (2019) recently identified aggregation-prone proteins in aneuploid W303, including proteins encoded on and off the amplified chromosomes. The hypothesis was raised that aggregation may be a beneficial mode of protein-dosage compensation (Brennan et al., 2019). However, that aggregation is a hallmark of *ssd1* deficiency, which itself causes aneuploidy sensitivity, argues against a beneficial function of aggregates. Instead, it points to a protective role for Ssd1 in handling aggregation-prone proteins. Consistent with this notion, the set of 22 proteins most prone to aggregation across W303 aneuploids is enriched for proteins encoded by Ssd1-bound transcripts identified in our study (p=0.005, hypergeometric test). Furthermore, as a group, proteins encoded by Ssd1 targets are predicted to display substantially higher fractions of disordered regions (based both on median IUPred score compared to all proteins and the fraction of residues with scores > 0.5, Mann Whitney p<2e-16); the trends remain significant even after disordered cell-wall proteins are removed from consideration. Although details of Ssd1’s function remain to be worked out, these results are consistent with a role for Ssd1 in regulating where and when mRNAs are translated to minimize aggregation and misfolding, and to enable normal cells to handle extra chromosomes.

Our results also reveal that Ssd1 affects mitochondrial physiology in aneuploid cells. Defects in mitochondrial function and cytosolic proteome management have long been linked, in neurological syndromes, aging, and even aneuploidy. Disruption of mitochondrial protein folding, import, and localization induces cytosolic protein stress and triggers cytosolic proteostasis systems (Wrobel et al., 2015; Wang and Chen, 2015; Nargund et al., 2012), consistent with our observation that CCCP induces cytosolic Hsp104 foci (Figure 6B). Conversely, clearance of misfolded cytosolic proteins relies on mitochondria: in addition to providing sufficient ATP for chaperone function, mitochondria can retain and import misfolded cytosolic proteins for sequestration and degradation (Ruan et al., 2017; Zhou et al., 2014). Extreme cytosolic misfolding, for example aggregated Huntington protein, perhaps consequently causes mitochondrial dysfunction (Franco-Iborra et al., 2018; Ocampo et al., 2010). Defects in these processes also co-occur in aneuploid syndromes, which produce altered mitochondrial morphology and function and premature aging phenotypes (Oromendia and Amon, 2014; Bambrick and Fiskum, 2008; Chang and Min, 2005). It is possible that mitochondrial defects in *ssd1****Δ*** cells arise as a secondary consequence of Ssd1 dysfunction; however, that Ssd1 binds several nuclear-encoded mitochondrial mRNAs, controls protein abundance of several of them, and purifies with mitochondria-enriched fractions raises the possibility of a more direct function. Given its role in localizing cell-wall mRNAs to the bud neck during division, Ssd1 may play a broader role in localizing and/or handling mitochondrial mRNAs – we provide evidence for one, *MMR1*, which showed a defect in localization patterns consistent with a defect in mitochondrial inheritance.

Our model that normal wild strains can handle the stress of extra chromosomes but exist near their buffering capacity is compatible with results from other systems. Some, but notably not all, aneuploid mouse and human cell lines show indirect signs of proteome stress, including increased autophagy and sensitivity to 17-AAG that inhibits Hsp90 chaperone (which is also required for proper chromosome segregation, confounding interpretation Chen et al., 2012). However, not all aneuploid lines display these signatures (Santaguida et al., 2015; Stingele et al., 2013; Stingele et al., 2012; Donnelly et al., 2014; Tang et al., 2011) and observed phenotypes are reportedly weaker than seen in W303 aneuploids (Brennan et al., 2019), as predicted by our study. While some phenotypic differences may result from differences in the load and identity of the aneuploid chromosome, another possibility is that proteostasis stress is not a universal feature of aneuploid cells. Rather, it may reflect a variable response influenced by environmental, developmental, or genetic differences in mitochondrial/proteostatic buffering capacity across lines. It has long been known that trisomy 21 produces phenotypes of variable severity in Down syndrome (DS), implicating genetic modifiers that augment tolerance (Antonarakis, 2017). A recent proteomic study showed that proteomes of unrelated DS skin fibroblasts showed some commonalities, including down-regulation of nuclear-encoded mitochondrial proteins, while other responses (such as altered lysosome activity) were variable across unrelated individuals and may thus contribute to variable DS severity (Liu et al., 2017). It is possible that natural genetic variation in wild yeast strains also contributes to natural variation in aneuploidy sensitivity. Interestingly, a recent large-scale genome sequencing study reported at least five truncated *SSD1* alleles segregating in yeast populations (Figure 6—source data 1) (Peter et al., 2018). The power of yeast genetics provides an opportunity to identify other modifiers of aneuploidy tolerance.

Ssd1 is orthologous to human Dis3L2 (Heinicke et al., 2007), an RNA binding protein best characterized for its ability to degrade poly-uridylated RNAs targeted for decay by terminal-uridyl transferases (TUTases) (Astuti et al., 2012; Chang et al., 2013; Lubas et al., 2013; Malecki et al., 2013; Morris et al., 2013; Ustianenko et al., 2013). Ssd1 is thought to have lost its catalytic activity (perhaps concomitant with loss of TUTase enzymes from *S. cerevisiae*; Uesono et al., 1997; Viegas et al., 2015). Dis3L2 was first identified in the causal mapping of Perlman syndrome, characterized by cellular over-growth, and is also implicated in Wilms tumor (Astuti et al., 2012). Dis3L2 shares several features with Ssd1: both can localize to the cytosol and nucleus, both bind RNAs and interact with P-bodies, and ablation of both proteins produces protein inclusion bodies (Astuti et al., 2012; Malecki et al., 2013; Mori et al., 2018; Liu et al., 2018; Thomas et al., 2015). Dis3L2 is also implicated in apoptosis trigged by mitochondrial signals (Liu et al., 2018; Thomas et al., 2015). Remarkably, mutation of Dis3L2 is also linked to aneuploidy: knockdown of Dis3L2 actually increases chromosome instability, leading to chromosome loss and aneuploidy (Astuti et al., 2012). Dissecting its role in aneuploidy syndromes is an exciting avenue for future work.

## Materials and methods

**Key resources table:** 

Reagent type (species) or resource	Designation	Source or reference	Identifier	Additional information
Gene (kan^r^)	kan^r^	Yeast Knockout Collection; Horizon Discovery		kanMX
Gene (*Klebsiella pneumoniae*)	hph	pAG26; Goldstein AL, McCusker JH		hphMX
Gene (*Streptomyces noursei*)	nat1	pPKI		natMX
Gtrain (*Saccharomyces cerevisiae*)	YPS1009 Mat a Euploid, hoΔ::HYG	this study	AGY731	Haploid, available on request from the Gasch Lab
Strain (*Saccharomyces cerevisiae*)	YPS1009 Mat alpha Euploid, hoΔ::HYG	this study	AGY732	Haploid, available on request from the Gasch Lab
Strain (*Saccharomyces cerevisiae*)	YPS1009_Chr12 Mat a Disome12, hoΔ::HYG	this study	AGY735	Haploid, available on request from the Gasch Lab
Strain (*Saccharomyces cerevisiae*)	YPS1009_Chr12 Mat alpha Disome12, hoΔ::HYG	this study	AGY736	Haploid, available on request from the Gasch Lab
Strain (*Saccharomyces cerevisiae*)	YPS1009 Mat a Euploid, hoΔ::HYG, ssd1Δ::KAN	this study	AGY1444	Haploid, available on request from the Gasch Lab
Strain (*Saccharomyces cerevisiae*)	YPS1009_Chr12 Mat a Disome12, hoΔ::HYG, ssd1Δ::KAN	this study	AGY1445	Haploid, available on request from the Gasch Lab
Strain (*Saccharomyces cerevisiae*)	YPS1009 Mat a Euploid, hoΔ::HYG, ssd1-Δ2 (KANMX removed)	this study	AGY1503	Haploid, marker rescued for plasmid expression, available on request from the Gasch Lab
Strain (*Saccharomyces cerevisiae*)	YPS1009_Chr12 Mat a Disome12, hoΔ::HYG, ssd1-Δ2 (KANMX removed)	this study	AGY1517	Haploid, marker rescued for plasmid expression, available on request from the Gasch Lab
Strain (*Saccharomyces cerevisiae*)	YPS1009 Mat a Euploid, hoΔ::HYG, SSD1-GFP-SSD1YPS1009-terminator-NATMX	this study	AGY1446	Haploid, GFP tagged Ssd1, available on request from the Gasch Lab
Strain (*Saccharomyces cerevisiae*)	YPS1009_Chr12 Mat a Disome12, hoΔ::HYG, SSD1-GFP-SSD1YPS1009-terminator-NATMX	this study	AGY1447	Haploid, GFP tagged Ssd1, available on request from the Gasch Lab
Strain (*Saccharomyces cerevisiae*)	YPS1009 Mat a Euploid, hoΔ::HYG, his3Δ::KAN	this study	AGY1504	Haploid, his3 deletion enabling HIS3 selection, available on request from the Gasch Lab
Strain (*Saccharomyces cerevisiae*)	YPS1009_Chr12 Mat a Disome12, hoΔ::HYG, his3Δ::KAN	this study	AGY1505	Haploid, his3 deletion enabling HIS3 selection, available on request from the Gasch Lab
Strain (*Saccharomyces cerevisiae*)	YPS1009 Mat a Euploid, hoΔ::HYG, his3Δ::KAN, ssd1Δ::KAN	this study	AGY1506	Haploid, his3 deletion enabling HIS3 selection, available on request from the Gasch Lab
Strain (*Saccharomyces cerevisiae*)	YPS1009_Chr12 Mat a Disome12, hoΔ::HYG, his3Δ::KAN, ssd1Δ::KAN	this study	AGY1507	Haploid, his3 deletion enabling HIS3 selection, available on request from the Gasch Lab
Strain (*Saccharomyces cerevisiae*)	YPS1009 Mat a Euploid, hoΔ::HYG, his3Δ::KAN, PET123-GFP-ADH1terminator-HIS3M × 6	this study	AGY1513	Haploid, available on request from the Gasch Lab
Strain (*Saccharomyces cerevisiae*)	YPS1009_Chr12 Mat a Disome12, hoΔ::HYG, his3Δ::KAN, PET123-GFP-ADH1terminator-HIS3M × 6	this study	AGY1514	Haploid, available on request from the Gasch Lab
Strain (*Saccharomyces cerevisiae*)	YPS1009 Mat a Euploid, hoΔ::HYG, his3Δ::KAN, HSP104-GFP-ADH1terminator-HIS3M × 6	this study	AGY1518	Haploid, available on request from the Gasch Lab
Strain (*Saccharomyces cerevisiae*)	YPS1009_Chr12 Mat a Disome12, hoΔ::HYG, his3Δ::KAN, HSP104-GFP-ADH1terminator-HIS3M × 6/HSP104	this study	AGY1519	Haploid, available on request from the Gasch Lab
Strain (*Saccharomyces cerevisiae*)	YPS1009 Mat a Euploid, hoΔ::HYG, his3Δ::KAN, ssd1Δ::KAN, HSP104-GFP-ADH1terminator-HIS3M × 6	this study	AGY1520	Haploid, available on request from the Gasch Lab
Strain (*Saccharomyces cerevisiae*)	YPS1009_Chr12 Mat a Disome12, hoΔ::HYG, his3Δ::KAN, ssd1Δ::KAN, HSP104-GFP-ADH1terminator-HIS3M × 6/HSP104	this study	AGY1521	Haploid, available on request from the Gasch Lab
Strain (*Saccharomyces cerevisiae*)	d-YPS1009_Chr12.2n Euploid	Hose et al.	AGY613	Diploid, available on request from the Gasch Lab
Strain (*Saccharomyces cerevisiae*)	d-YPS1009_Chr12.4n Aneuploid	Hose et al.	AGY614	Diploid, available on request from the Gasch Lab
Strain (*Saccharomyces cerevisiae*)	d-YPS1009_Chr12.2n Euploid, ssd1Δ::KAN/ssd1Δ::KAN	this study	AGY1560	Diploid, available on request from the Gasch Lab
Strain (*Saccharomyces cerevisiae*)	d-YPS1009_Chr12.4n Aneuploid, ssd1Δ::KAN/ssd1Δ::KAN	this study	AGY1561	Diploid, available on request from the Gasch Lab
Strain (*Saccharomyces cerevisiae*)	W303 Mat a Euploid ade2-1 his3-11,15 leu2-3,112 trp1-1 ura3-1 can1-100 Gal+ ade16Δ::KAN	this study	AGY1387	Haploid, available on request from the Gasch Lab
Strain (*Saccharomyces cerevisiae*)	W303_Chr12 Mat a Disome12 ade2-1 his3-11,15 leu2-3,112 trp1-1 ura3-1 can1-100 Gal+ ade16Δ::KAN/ade16Δ::HYG	this study	AGY768	Haploid, available on request from the Gasch Lab
Strain (*Saccharomyces cerevisiae*)	W303 Mat a Euploid ADE2+ his3-11,15 leu2-3,112 trp1-1 ura3-1 can1-100 Gal+ ade16Δ::KAN	this study	AGY1388	Haploid, available on request from the Gasch Lab
Strain (*Saccharomyces cerevisiae*)	W303_Chr12 Mat a Disome12 ADE2+ his3-11,15 leu2-3,112 trp1-1 ura3-1 can1-100 Gal+ ade16Δ::KAN/ade16Δ::HYG	this study	AGY1389	Haploid, available on request from the Gasch Lab
Strain (*Saccharomyces cerevisiae*)	W303 Mat a Euploid ade2-1 his3-11,15 leu2-3,112 trp1-1 ura3-1 can1-100 Gal+	this study	AGY103	Haploid, available on request from the Gasch Lab
Strain (*Saccharomyces cerevisiae*)	W303_Chr8 Mat a Disome8 ade2-1 his3-11,15 leu2-3,112 trp1-1 ura3-1 can1-100 Gal+	this study	AGY1495	Haploid, available on request from the Gasch Lab
Strain (*Saccharomyces cerevisiae*)	W303_Chr8-15 Mat a Disome8,15 ade2-1 his3-11,15 leu2-3,112 trp1-1 ura3-1 can1-100 Gal+	this study	AGY1496	Haploid, available on request from the Gasch Lab
Strain (*Saccharomyces cerevisiae*)	W303_Chr8-10-16 Mat a Disome8,10,16 ade2-1 his3-11,15 leu2-3,112 trp1-1 ura3-1 can1-100 Gal+	this study	AGY1497	Haploid, available on request from the Gasch Lab
Strain (*Saccharomyces cerevisiae*)	YPS1009xW303 (sp100) Mat alpha Disome12 trp1-1 ade16Δ::KAN HYG+	this study	AGY1548	Haploid, available on request from the Gasch Lab
Strain (*Saccharomyces cerevisiae*)	d-NCYC110 Euploid	Hose et al.	AGY729	Diploid, available on request from the Gasch Lab
Strain (*Saccharomyces cerevisiae*)	d-NCYC110_Chr8-4n Aneuploid	Hose et al.	AGY703	Diploid, available on request from the Gasch Lab
Strain (*Saccharomyces cerevisiae*)	d-NCYC110 Euploid, ssd1Δ::KAN/ssd1Δ::KAN	this study	AGY1493	Diploid, available on request from the Gasch Lab
Strain (*Saccharomyces cerevisiae*)	d-NCYC110_Chr8-4n Aneuploid, ssd1Δ::KAN/ssd1Δ::KAN	this study	AGY1494	Diploid, available on request from the Gasch Lab
Strain (*Saccharomyces cerevisiae*)	KCY40 (or VC580) Euploid, hoΔ::MFA^prom^-HYGMX-NATMX	Hose et al.	AGY806	Haploid
Strain (*Saccharomyces cerevisiae*)	KCY40 (or VC580) Disome8, hoΔ::MFA^prom^-HYGMX-NATMX	Hose et al.	AGY1105	Haploid
Strain (*Saccharomyces cerevisiae*)	KCY40 (or VC580) Euploid, hoΔ::MFA^prom^-HYGMX-NATMX, ssd1Δ::KAN	this study	AGY1385	Haploid, available on request from the Gasch Lab
Strain (*Saccharomyces cerevisiae*)	KCY40 (or VC580) Disome8, hoΔ::MFA^prom^-HYGMX-NATMX, ssd1Δ::KAN	this study	AGY1386	Haploid, available on request from the Gasch Lab
Strain (*Saccharomyces cerevisiae*)	YPS1009_Chr12 Mat a Disome12, hoΔ::HYG + pPKI	this study	AGY735 transformed with plasmid	Haploid, available on request from the Gasch Lab
Strain (*Saccharomyces cerevisiae*)	YPS1009_Chr12 Mat a Disome12, hoΔ::HYG, ssd1Δ::KAN + pPKI	this study	ABY1445 transformed with plasmid	Haploid, available on request from the Gasch Lab
Strain (*Saccharomyces cerevisiae*)	YPS1009_Chr12 Mat a Disome12, hoΔ::HYG, ssd1Δ::KAN + pJH1-SSD1-W303	this study	ABY1445 transformed with plasmid	Haploid, available on request from the Gasch Lab
Strain (*Saccharomyces cerevisiae*)	YPS1009_Chr12 Mat a Disome12, hoΔ::HYG, ssd1Δ::KAN + pJH1-SSD1-YPS1009	this study	ABY1445 transformed with plasmid	Haploid, available on request from the Gasch Lab
Strain (*Saccharomyces cerevisiae*)	d-NCYC110_Chr8-4n Aneuploid + pJH1	this study	AGY703 tranformed with plasmid	Diploid, available on request from the Gasch Lab
Strain (*Saccharomyces cerevisiae*)	d-NCYC110_Chr8-4n Aneuploid, ssd1Δ::KAN/ssd1Δ::KAN + pJH1	this study	AGY1494 transformed with plasmid	Diploid, available on request from the Gasch Lab
Strain (*Saccharomyces cerevisiae*)	d-NCYC110_Chr8-4n Aneuploid, ssd1Δ::KAN/ssd1Δ::KAN + pJH1-SSD1-W303	this study	AGY1494 transformed with plasmid	Diploid, available on request from the Gasch Lab
Strain (*Saccharomyces cerevisiae*)	d-NCYC110_Chr8-4n Aneuploid, ssd1Δ::KAN/ssd1Δ::KAN + pJH1-SSD1-YPS1009	this study	AGY1494 transformed with plasmid	Diploid, available on request from the Gasch Lab
Strain (*Saccharomyces cerevisiae*)	W303 Mat a Euploid ade2-1 his3-11,15 leu2-3,112 trp1-1 ura3-1 can1-100 Gal+ ade1::HIS3, lys2::KAN	Torres et al.	AGY487	Haploid
Strain (*Saccharomyces cerevisiae*)	W303_Chr12 Mat a Disome12 ade2-1 his3-11,15 leu2-3,112 trp1-1 ura3-1 can1-100 Gal+ ade16::HIS3 ade16::KAN	Torres et al.	AGY488	Haploid
Strain (*Saccharomyces cerevisiae*)	W303 Mat a Euploid ade2-1 his3-11,15 leu2-3,112 trp1-1 ura3-1 can1-100 Gal+ ade1::HIS3, lys2::KAN + pJH1	this study	AGY487 transformed with plasmid	Haploid, available on request from the Gasch Lab
Strain (*Saccharomyces cerevisiae*)	W303_Chr12 Mat a Disome12 ade2-1 his3-11,15 leu2-3,112 trp1-1 ura3-1 can1-100 Gal+ ade16::HIS3 ade16::KAN + pJH1	this study	AGY488 transformed with plasmid	Haploid, available on request from the Gasch Lab
Strain (*Saccharomyces cerevisiae*)	W303_Chr12 Mat a Disome12 ade2-1 his3-11,15 leu2-3,112 trp1-1 ura3-1 can1-100 Gal+ ade16::HIS3 ade16::KAN + pJH1-SSD1-YPS1009	this study	AGY488 transformed with plasmid	Haploid, available on request from the Gasch Lab
Antibody	Rabbit polyclonal Anti-GFP	Abcam	Abcam catalog #ab290	Rabbit polyclonal; 1:2000
Antibody	Mouse monoclonal Anti-Actin	Thermo Fisher Scientific	Thermo Fisher Scientific catalog #MA1-744	Mouse monoclonal; 1:1000
Antibody	Mouse monoclonal Anti-COX2	Abcam	Abcam catalog #ab110271	Mouse monoclonal; 1:500
Antibody	Mouse monoclonal Anti-DPM1	Abcam	Abcam catalog #ab113686	Mouse monoclonal; 1:250
Antibody	Mouse monoclonal Anti-VPH1	Abcam	Abcam catalog #ab113683	Mouse monoclonal; 1:1000
Recombinant DNA reagent	pXIPHOS	GenBank accession MG897154	PAM sgRNA sequence (GAATCGAATG CAACCGGCGC) that targeted KanMX	Higgins et al., Wrobel et al.
Recombinant DNA reagent	pPKI	this study	AGB185	CEN plasmid with the natMX selection marker.
Recombinant DNA reagent	pJH1	this study	AGB090	CEN plasmid derived from pKI that has natMX selection marker. pJH is equivalent to pKI except for a fragment of unexpressed DNA that was removed during generation.
Recombinant DNA reagent	pJH1-SSD1-YPS1009	this study		ORF + 1000 bp upstream and 337 bp downstream of SSD1 from YPS1009 genomic DNA. Plasmid has natMX selection marker
Recombinant DNA reagent	pJH1-SSD1-W303	this study		ORF + 1000 bp upstream and 337 bp downstream of SSD1 from aW303 genomic DNA. Plasmid has natMX selection marker
Recombinant DNA reagent	Molecular Barcoded Yeast (MoBY) v2.0 ORF Library	other	obtained from Great Lakes Bioenergy Research Center (GLBRC)	Ho, CH. et al. A molecular barcoded yeast ORF library enables mode-of-action analysis of bioactive compounds. Nat. Biotech. 27 (Holland and Cleveland, 2012), 369–377 (2009).
Sequence-based reagent	*MMR1* FISH probes	Stellaris		designed against MMR1 mRNA
Sequence-based reagent	Mitochondrial rRNA FISH probes	Stellaris		designed against 15 s and 21 s rRNA
Peptide, recombinant protein	von Hippel-Lindau (VHL) tumor suppressor	Kaganovich et al.	Addgene catalog #21053	Kaganovich D, Kopito R, Frydman J. Misfolded proteins partition between two distinct quality control compartments. Nature. 2008 Aug 28. 454 (7208):1088–95.
Peptide, recombinant protein	*Aequorea victoria* GFP (S65T)	Huh et al.		Huh W, Falvo JV, Gerke LC, Carroll AS, Howson RW, Weissman JS, and O'Shea EK (2003) Global Analysis of Protein Localization in Budding Yeast Nature 425:686–691.
Commercial assay or kit	Mitochondrial Yeast Isolation Kit	Abcam	Abcam catalog #ab178779	
Commercial assay or kit	Illumina TruSeq Total RNA Stranded	Illumina	Illumina catalog #20020597; previously RS-122–2203	
Commercial assay or kit	NEBNext Ultra DNA Library Prep Kit for Illumina	New England Biolabs	NEB catalog #E7370L	
Commercial assay or kit	Yeast Mitochondrial Stain Sampler Kit	Thermo Fisher Scientific	Thermo Fisher Scientific catalog #Y7530	
Chemical compound, drug	Nourseothricin-dihydrogen sulfate(clonNAT)	Werner BioAgents	Werner BioAgents catalog #5.005.000	
Chemical compound, drug	4',6-Diamidino-2-phenylindole, dihydrochloride (DAPI)	Thermo Fisher Scientific	Thermo Fisher Scientific catalog #PI62247	
Chemical compound, drug	Carbonyl cyanide 3-chlorophenylhydrazone (CCCP)	Sigma-Aldrich	Sigma catalog #C2759	
Chemical compound, drug	Radicicol, Humicola fuscoatra	A.G. Scientific	A.G. Scientific catalog #R-1130	
Chemical compound, drug	GFP-Trap Magnetic Agarose	Chromotek	Chromotek catalog #gtma-20	

### Strains and plasmids

Strains used in this study are listed in the Resource Table. W303_Chr8, W303_Chr8-Chr15, and W303_Chr8-Chr10-Chr16 were generated using the method of Chen et al. (2012), passaging 16 generations in 20 μg/mL radicicol (A.G. Scientific) and plating on 8 or 16 μg/mL fluconazole to select for Chr8 aneuploidy. Karyotype was determined by array-comparative genomic hybridization and sequencing. W303 strains shown in Figure 1E were grown in SC-his + G418 to maintain marked copies of Chr12 (or corresponding markers in the otherwise isogenic wild type Torres et al., 2007). In general, deletions were generated by homologous recombination of relevant makers (e.g. *KAN-MX* or *HIS3*) into the designated locus, followed by diagnostic PCR to confirm correct integration and absence of the target gene. Because *ssd1Δ* cultures lose extra chromosomes (perhaps simply due to overtaking of the culture by stochastic euploid revertants), deletions were generated in wild-type strains that were then crossed to YPS1009_Chr12 *ssd1Δ*, followed by tetrad dissection to isolate aneuploid spore clones with desired genotypes. In all cases, aneuploidy was confirmed and periodically checked through diagnostic qPCR of one or two genes on the affected chromosomes (*AAT1* and *SDH2*) normalized to a single-copy gene elsewhere in the genome (*ERV25 or ACT1*) – normalized ratios close to two reflect gene duplication, and ratios between 1.2–1.8X indicated partial loss of aneuploidy in the cell population. GFP-tagged genes were generated by integrating a *GFP-ADH2terminator-HIS*3 cassette (Huh et al., 2003) via homologous recombination into strain series AGY1504-1507 in which *HIS3* was previously deleted by replacement with *KAN-MX* marker. In the case of *SSD1-GFP* strains, a cassette consisting of GFP followed by the native *SSD1* terminator, 337 bp downstream of *SSD1^YPS1009^*, was generated by PCR sewing with the *NAT-MX* marker. In all cases, cloned or tagged genes were confirmed by sequencing. *SSD1^YPS1009^* plus 1000 bp upstream and 300 bp downstream was cloned into a pRS-derived CEN plasmid for complementation. Because YPS1009_Chr12 *ssd1Δ* cannot tolerate the 2-micron plasmid, the VHL-GFP gene plus promoter and terminator sequences were cloned from pESC-LEU-GFP-VHL (ADDGENE #21053) into a pRS-derived CEN plasmid. Human VHL cannot fold without cofactors but is typically cleared from cells through proteasome activity (McClellan et al., 2005). Accumulation of VHL-GFP foci is generally taken as an inability to clear misfolded proteins.

### Growth conditions

Unless otherwise noted, strains were cultured for ~3 generations into log phase in rich YPD medium at 30°C, with the exception of microscopy experiments where cells were grown in low-fluorescence synthetic-complete medium and imaged live. Induction of VHL-GFP was performed by growing cells in YP with 2% raffinose + 2% galactose for 4 hr. Wild-type strains shown in Figure 6C–D were grown over-night in log-phase before addition of 1 ug/mL nourseothricin (Werner BioAgents, Jena, Germany) or 25 uM CCCP (Millipore-Sigma, St. Louis, MO). Growth rates were calculated by exponentially fitting changes in optical density. Relative final OD in Figure 6D was measured in biological triplicate after 24 hr growth of YPS1009, NCYC110, and W303 strains exposed to 25 uM CCCP with 0.5 ug/mL (NCYC, W303) or 1 ug/mL (YPS1009) NTC, respectively. Aneuploidy was periodically verified through diagnostic qPCR as described above. Expected growth rates under an additive model were estimated based on the fold-defect in one condition (*e.g.* aneuploidy versus euploidy) multiplied by the fold-defect in a second condition (*e.g.* NTC sensitivity in the euploid); significant differences in observed versus expected data were assessed with replicate-paired T-tests. Unless otherwise noted, all studies used at least biological triplicates with data represented as the average and standard deviation (except count data in Figures 2B–C, 4D, 5C–D and 6B in which average and standard error of the mean across biological replicates is shown).

### Bulk-segregant phenotyping and mapping

Haploid strain AGY736 (YPS1009_Chr12 Mat alpha *ho::HYGMX*) was crossed with AGY768 (W303_Chr12 Mat a) and the resulting diploid sporulated and dissected evenly on agar plates. Colony diameter after 72 hr was scored for 208 spores; 76 spores ranking in the smallest ~40% of the distribution were scored for their propensity to lose Chr12 within 20 culture generations of growth: each spore was passaged for 2 days in liquid YPD, after which genomic DNA was isolated and Chr12 abundance scored by diagnostic PCR as described above. Loss of Chr12 signal was taken as aneuploidy sensitivity (20 spores, Pool A1) whereas cells that maintained Chr12 signal were taken as enriched for aneuploid tolerant cells (40 spores, Pool B1). Spore sp100 (Mat alpha *ADE2 HIS3 LEU2 trp- URA3*) that was prototrophic for influential markers was selected, its aneuploidy status verified by qPCR, and it was backcrossed to AGY735 (YPS1009_Chr12 Mat a *ho::HYGMX)*. 37 segregants were scored only for their propensity to lose aneuploidy after 2 days of passage, generating an aneuploidy-sensitive pool (10 spores, Pool A2) and a pool enriched for aneuploidy-tolerant strains (25 spores, Pool B2). A control cross of euploid hYPS1009 X euploid W303 was generated and phenotyped for colony size as above. 46 and 50 spores were taken as ‘small’ (colony diameter <437 square pixels) or ‘large’ (colony >591 square pixels) for Pool D and Pool F, respectively.

Each clone was grown to saturation, an equal volume of each culture pooled appropriately, and genomic DNA isolated (Qiagen, Germantown, MD) from ach pool. Pooled genomic DNA was sequenced using NEBNext Ultra DNA Library Prep Kit for Illumina on an Illumina HiSeq 2000 to an average of 20M 100 bp reads per pool. To avoid potential mapping biases, an artificial reference genome was created where single nucleotide polymorphisms (SNPs) between the two parental genomes were substituted for a third allele not present in either genome. Reads from sequenced pools were aligned to the artificial reference using bwa-mem (Li and Durbin, 2010). A pileup at known parental SNPs was created using samtools (Li et al., 2009), and allele counts at each SNP were calculated. SNP positions were filtered to retain SNPs with at least 15X coverage, both parental alleles scored, and allele frequency between 0.1–0.9 to eliminate false signals during bulk segregant analysis. Bulk-segregant analysis was performed using MULTIPOOL (v 0.10.1) (Edwards and Gifford, 2012) run across ~60,000 SNPs in contrast mode using the default recombination fraction (3300 cM) with -N set to the number of segregants in the aneuploidy-sensitive/small-colony pool in each cross (A1 = 20; A2 = 10; D = 46). Potential QTLs were identified at loci where allele frequency varied the greatest between the two pools. *SSD1* was validated as the causal locus through gene deletions and complementation as shown in Figure 1. Sequencing data for each pool are available in the Short Read Archive (SRA) under access number PRJNA548343, and MULTIPOOL output files are available in Supplementary file 1, as described in Edwards and Gifford (2012).

### RNA sequencing, RNA immunoprecipitation, and plasmid barcode sequencing

RNA-seq was done as previously described (Jovaisaite et al., 2014) using total RNA isolated from log-phase cultures. Illumina reads were mapped to the S288c genome substituted with SNPs from YPS1009, NCYC110, or W303 as called in Sardi et al. (2018), using bwa-meme. In general, data represent the average of biological triplicate, with the exception of h-YPS1009 strains shown in Figure 2 done in quadruplicate and W303_Chr8-Chr15 and W303_Chr8-Chr10-Chr16 done in duplicate. Replicates for each strain suite were paired on the same day, enabling replicate-paired statistical analysis, done in edgeR (Robinson et al., 2010). Genes in Figure 2 were selected by considering both YPS1009_Chr12 *ssd1Δ* versus YPS1009_Chr12 wild type and NCYC110_Chr8 *ssd1Δ* versus NCYC110_Chr8 wild type. Hierarchical clustering was performed using Cluster 3.0 (Eisen et al., 1998) and visualized in Java Treeview (Saldanha, 2004). Functional enrichment of GO terms was performed using the program SetRank (Simillion et al., 2017). Activation of the UPR was inferred from enrichment of Hac1 targets among induced genes (p<1e-4, hypergeometric test, compiled in Chasman et al., 2014), and signatures of mito-CPR was indicated as up-regulation of Pdr3 targets including *CIS3* as reported in Weidberg and Amon (2018). Sequencing data are available from the GEO database under accession number GSE132425. Processed data are also available in Supplementary file 2.

RNA-immunoprecipitation (RIP) was performed similar to previously described (Jansen et al., 2009) with the following modifications: Cell lysate was treated with RQ1 RNase-free DNase (Promega, Madison, WI) for 15 min at room temperature, an aliquot was removed as the input material, and RNA was immunoprecipitated using GFP-Trap Magnetic Agarose (Chromotek, Planegg-Martinsried, Germany) against Ssd1-GFP from euploid and aneuploid lysate for 1 hr at 4°C (due to Ssd1-GFP degradation with longer incubation). An identical procedure was performed with untagged YPS1009 cells as a mock-RIP. Recovered RNA was subjected to Illumina sequencing as described above. RIP-seq was performed in duplicate for euploid and for aneuploid cells; bound transcripts were identified through combined edgeR (Robinson et al., 2010) analysis of the four RIP-seq samples, contrasting RIP to input for each sample and then to mock-IP normalized to its own input. Bound transcripts were taken as those with FDR < 0.05 (Supplementary file 2). Sequencing data are available from the GEO database under accession number GSE132425.

The suite of YPS1009_Chr12 strains (AGY731, AGY735, AGY1503, AGY1517) was transformed with Moby 2.0 high-copy expression library (Magtanong et al., 2011) and an aliquot removed as the starting pool. Cells were grown in biological triplicate for five generations in YPD medium, plasmid DNA collected from the starting and ending pools, and barcodes sequenced as previously described (Magtanong et al., 2011). Pools were normalized by total barcode reads per sample and fitness costs taken as the log2(fold change) in barcode abundance after versus before outgrowth. Data in Figure 2D represent the distribution of replicate-averaged data.

### Proteomics

Cell pellets were resuspended in 6 M guanidine HCl and boiled for 5–10 min; proteins were precipitated with methanol up to 90%, spun 5 min at 15 K g, and resuspended in lysis buffer (8 M urea, 100 mM Tris, pH = 8.0, 10 mM TCEP, 40 mM chloroacetamide). Samples were diluted to 1.5 M urea and digested overnight at room temperature with LysC (Wako Chemicals, USA) and for 3 hr with trypsin (Promega, USA) at 1:50 enzyme to protein ratio. Samples were desalted using Strata X columns (Phenomenex Strata-X Polymeric RP, USA). For LC-MS/MS, samples were resuspended in 0.2% formic acid and separated via reversed phase (RP) chromatography. 2 µg of tryptic peptides were injected onto a capillary RP column prepared in-house and packed with 1.7 μm diameter Bridged Ethylene Hybrid C18 particles as described in Shishkova et al. (2018). Columns were installed onto Dionex nanoHPLC (Thermo, Sunnyvale CA) and heated to 50°C using a home-built column heater. Mobile phase buffer A was composed of water and 0.2% formic acid, mobile phase B - 70% ACN and 0.2% formic acid. Samples were separated over a 120 min gradient at flow rate of 325 nl/min. Peptide cations were converted into gas-phase ions via electrospray ionization and analyzed using a Thermo Orbitrap Fusion Lumos (Thermo, San Jose CA) mass spectrometer, according to the previously published methods (Hebert et al., 2018). Raw data were searched using MaxQuant (v. 1.6.1.0) against *Saccharomyces cerevisiae* database (SGD, downloaded 10.15.2018). Searches were performed using precursor mass tolerance of 27 ppm and a product mass tolerance of 0.3 Da. Proteins were identified and quantified via MaxLFQ using default settings with enabled ‘Match between runs,’ requiring LFQ ratio of 1, and MS/MS spectra not required for LFQ comparisons. Raw data are available in the PRIDE database (Project accession # PXD013847). Prior to publication, reviewers can access the files using the following credentials: Username: reviewer95858@ebi.ac.uk, Password: 6w9IaMi3. Processed data and a list of proteins shown in Figure 3 are available in Supplementary file 2, and normalized protein abundance data are available in Supplementary file 3.

### Mitochondrial fractionation, microscopy and single-molecule smFISH

Organelle-enriched and -depleted fractions were generated for euploid (AGY1446) and aneuploid YPS1009_Ch12 *SSD1-GFP* (AGY1447) and untagged *SSD1* cells as a control, using Mitochondrial Yeast Isolation Kit (Abcam, Cambridge, United Kingdom) according to manufacturer protocol with slight modifications to minimize protein degradation. Western blots were developed using anti-GFP ab290 (Abcam), anti-Actin MA1-744 (Thermo Fisher Scientific), anti-Cox2 ab110271 (Abcam), anti-Vph1 ab113683 (Abcam), and anti-Dpm1 ab113686 (Abcam) on a Li-COR Odyssey instrument (Model 9120). Cells for microscopy were plated on plain or poly-L-Lysine coated slides and either single images (HSP104-GFP in Figure 6) or z-stack images (all other microscopy) every 0.5 µm were acquired with an EVOS FL Auto two equipped with an RFP EVOS light cube. Z-stacks were collapsed into a single image with EVOS software for publication. Mitochondria in Figure 4C were visualized with Rhodamine B Hexyl Ester (ThermoFisher, R648MP) according to manufacturer’s protocol; images represent an overlay of the bright-field image onto the fluorescence image to highlight cell boundaries. Cells were scored by marking total cells in bright-field images and the scoring presence or absence of Rhodamine B Hexyl Ester signal. A minimum of 380 cells were scored per strain across three biological replicates. Very similar results were obtained tracking Pet123-GFP signal.

smFISH was performed as previously described (Gasch et al., 2017) except performed on an EVOS FL Auto two and with transcripts detected manually in FIJI (Schindelin et al., 2012). FISH probe sets were designed against *MMR1* (conjugated to Quasar 670) and mitochondrial 15 s and 21 s rRNAs (conjugated to Quasar 570, Stellaris, Middlesex, United Kingdom). Mitochondrial morphology in Figure 3D was quantified using mitochondrial rRNA probes, which produced images very similar to Rhodamine staining but enabled visualization independent of mitochondrial membrane potential. Morphology was scored in each cell manually using the multi-point tool in FIJI, recording the number of cells with any tubular, any globular, or only globular morphologies. Cells with fragmented mitochondria were defined as those with at least three discontinuous fragments from the tubular structure or having at least three fragments in addition to the largest globular focus. >200–400 cells were scored for all microscopy experiments and across multiple biological replicates per strain. *MMR1* localization was scored by identifying buds (scored as cells lacking DAPI or containing bar nuclei by DAPI staining) and scoring those either containing or lacking *MMR1* transcripts.

## Data Availability

Sequencing data for genetic mapping are available in the Short Read Archive (SRA) under access number PRJNA548343, and MULTIPOOL output files are available in Supplementary file 1. RNA and RNA Immunoprecipitation (RIP) sequencing data are available from the GEO database under accession number GSE132425, and processed data are also available in Supplementary file 2. Raw proteomic data are available in the PRIDE database (Project accession # PXD013847); processed data are available in Supplementary file 2, and normalized protein abundance data are available in Dataset 3. The following datasets were generated: EvgeniaShishkovaJoshuaJ Coon2020Aneuploid yeast proteomes in wild-type and ssd1 strains.PRIDEPXD013847 Hose2020DNA mapping dataNCBI BioProjectPRJNA548343 HoseJ2020The Genetic Basis of Aneuploidy Tolerance in Wild YeastNCBI Gene Expression OmnibusGSE13242510.7554/eLife.52063PMC697051431909711

## Additional files

Supplementary file 1. Zipped file with MULTIPOOL output files, as described in the MULTIPOOL manual, comparing pools A1 versus B1, A2 versus B2, and D versus F as described in Materials and methods. Plots represent the W303 allele frequency in the aneuploidy-sensitive or small-colony pools (red) versus the aneuploidy-tolerant or larger-colony pools (blue).

Supplementary file 2. Compiled information and data. Genes bound by Ssd1 (column 3), genes shown in Figure 2A (column 4), proteins shown in Figure 3 (column 5), log2(fold change) in mRNA abundance for denoted strains (columns 6–42), log2(fold change) in protein abundance for denoted strains (columns 46–56).

Supplementary file 3. Normalized absolute protein abundance for each sample, see Materials and methods.

Transparent reporting form

## References

[bib1] Antonarakis SE (2017). Down syndrome and the complexity of genome dosage imbalance. Nature Reviews Genetics.

[bib2] Astuti D, Morris MR, Cooper WN, Staals RH, Wake NC, Fews GA, Gill H, Gentle D, Shuib S, Ricketts CJ, Cole T, van Essen AJ, van Lingen RA, Neri G, Opitz JM, Rump P, Stolte-Dijkstra I, Müller F, Pruijn GJ, Latif F, Maher ER (2012). Germline mutations in DIS3L2 cause the Perlman syndrome of overgrowth and Wilms tumor susceptibility. Nature Genetics.

[bib3] Bambrick LL, Fiskum G (2008). Mitochondrial dysfunction in mouse trisomy 16 brain. Brain Research.

[bib4] Bennett RJ, Forche A, Berman J (2014). Rapid mechanisms for generating genome diversity: whole ploidy shifts, aneuploidy, and loss of heterozygosity. Cold Spring Harbor Perspectives in Medicine.

[bib5] Brennan CM, Vaites LP, Wells JN, Santaguida S, Paulo JA, Storchova Z, Harper JW, Marsh JA, Amon A (2019). Protein aggregation mediates stoichiometry of protein complexes in aneuploid cells. Genes & Development.

[bib6] Camougrand NM, Mouassite M, Velours GM, Guérin MG (2000). The "SUN" family: UTH1, an ageing gene, is also involved in the regulation of mitochondria biogenesis in Saccharomyces cerevisiae. Archives of Biochemistry and Biophysics.

[bib7] Camougrand N, Kissová I, Velours G, Manon S (2004). Uth1p: a yeast mitochondrial protein at the crossroads of stress, degradation and cell death. FEMS Yeast Research.

[bib8] Chang HM, Triboulet R, Thornton JE, Gregory RI (2013). A role for the Perlman syndrome exonuclease Dis3l2 in the Lin28-let-7 pathway. Nature.

[bib9] Chang KT, Min KT (2005). Drosophila Melanogaster homolog of down syndrome critical region 1 is critical for mitochondrial function. Nature Neuroscience.

[bib10] Chasman D, Ho YH, Berry DB, Nemec CM, MacGilvray ME, Hose J, Merrill AE, Lee MV, Will JL, Coon JJ, Ansari AZ, Craven M, Gasch AP (2014). Pathway connectivity and signaling coordination in the yeast stress-activated signaling network. Molecular Systems Biology.

[bib11] Chen G, Bradford WD, Seidel CW, Li R (2012). Hsp90 stress potentiates rapid cellular adaptation through induction of aneuploidy. Nature.

[bib12] Chernova TA, Wilkinson KD, Chernoff YO (2017). Prions, chaperones, and proteostasis in yeast. Cold Spring Harbor Perspectives in Biology.

[bib13] Dephoure N, Hwang S, O'Sullivan C, Dodgson SE, Gygi SP, Amon A, Torres EM (2014). Quantitative proteomic analysis reveals posttranslational responses to aneuploidy in yeast. eLife.

[bib14] Dodgson SE, Santaguida S, Kim S, Sheltzer J, Amon A (2016). The pleiotropic deubiquitinase Ubp3 confers aneuploidy tolerance. Genes & Development.

[bib15] Donnelly N, Passerini V, Dürrbaum M, Stingele S, Storchová Z (2014). HSF 1 deficiency and impaired HSP 90‐dependent protein folding are hallmarks of aneuploid human cells. The EMBO Journal.

[bib16] Donnelly N, Storchová Z (2015). Causes and consequences of protein folding stress in aneuploid cells. Cell Cycle.

[bib17] Edwards MD, Gifford DK (2012). High-resolution genetic mapping with pooled sequencing. BMC Bioinformatics.

[bib18] Eisen MB, Spellman PT, Brown PO, Botstein D (1998). Cluster analysis and display of genome-wide expression patterns. PNAS.

[bib19] Filteau M, Hamel V, Pouliot MC, Gagnon-Arsenault I, Dubé AK, Landry CR (2015). Evolutionary rescue by compensatory mutations is constrained by genomic and environmental backgrounds. Molecular Systems Biology.

[bib20] Fontanillas P, Landry CR, Wittkopp PJ, Russ C, Gruber JD, Nusbaum C, Hartl DL (2010). Key considerations for measuring allelic expression on a genomic scale using high-throughput sequencing. Molecular Ecology.

[bib21] Franco-Iborra S, Vila M, Perier C (2018). Mitochondrial quality control in neurodegenerative diseases: focus on Parkinson's Disease and Huntington's Disease. Frontiers in Neuroscience.

[bib22] Gasch AP, Spellman PT, Kao CM, Carmel-Harel O, Eisen MB, Storz G, Botstein D, Brown PO (2000). Genomic expression programs in the response of yeast cells to environmental changes. Molecular Biology of the Cell.

[bib23] Gasch AP, Hose J, Newton MA, Sardi M, Yong M, Wang Z (2016). Further support for aneuploidy tolerance in wild yeast and effects of dosage compensation on gene copy-number evolution. eLife.

[bib24] Gasch AP, Yu FB, Hose J, Escalante LE, Place M, Bacher R, Kanbar J, Ciobanu D, Sandor L, Grigoriev IV, Kendziorski C, Quake SR, McClean MN (2017). Single-cell RNA sequencing reveals intrinsic and extrinsic regulatory heterogeneity in yeast responding to stress. PLOS Biology.

[bib25] Hebert AS, Prasad S, Belford MW, Bailey DJ, McAlister GC, Abbatiello SE, Huguet R, Wouters ER, Dunyach JJ, Brademan DR, Westphall MS, Coon JJ (2018). Comprehensive Single-Shot proteomics with FAIMS on a hybrid orbitrap mass spectrometer. Analytical Chemistry.

[bib26] Heinicke S, Livstone MS, Lu C, Oughtred R, Kang F, Angiuoli SV, White O, Botstein D, Dolinski K (2007). The princeton protein orthology database (P-POD): a comparative genomics analysis tool for biologists. PLOS ONE.

[bib27] Helguera P, Seiglie J, Rodriguez J, Hanna M, Helguera G, Busciglio J (2013). Adaptive downregulation of mitochondrial function in down syndrome. Cell Metabolism.

[bib28] Hogan DJ, Riordan DP, Gerber AP, Herschlag D, Brown PO (2008). Diverse RNA-Binding proteins interact with functionally related sets of RNAs, suggesting an extensive regulatory system. PLOS Biology.

[bib29] Holland AJ, Cleveland DW (2012). Losing balance: the origin and impact of aneuploidy in Cancer. EMBO Reports.

[bib30] Hose J, Yong CM, Sardi M, Wang Z, Newton MA, Gasch AP (2015). Dosage compensation can buffer copy-number variation in wild yeast. eLife.

[bib31] Hu Z, Xia B, Postnikoff SD, Shen ZJ, Tomoiaga AS, Harkness TA, Seol JH, Li W, Chen K, Tyler JK (2018). Ssd1 and Gcn2 suppress global translation efficiency in replicatively aged yeast while their activation extends lifespan. eLife.

[bib32] Huh WK, Falvo JV, Gerke LC, Carroll AS, Howson RW, Weissman JS, O'Shea EK (2003). Global analysis of protein localization in budding yeast. Nature.

[bib33] Itoh T, Toh-E A, Matsui Y (2004). Mmr1p is a mitochondrial factor for Myo2p-dependent inheritance of mitochondria in the budding yeast. The EMBO Journal.

[bib34] Jansen JM, Wanless AG, Seidel CW, Weiss EL (2009). Cbk1 regulation of the RNA-binding protein Ssd1 integrates cell fate with translational control. Current Biology.

[bib35] Jovaisaite V, Mouchiroud L, Auwerx J (2014). The mitochondrial unfolded protein response, a conserved stress response pathway with implications in health and disease. Journal of Experimental Biology.

[bib36] Kaeberlein M, Andalis AA, Liszt GB, Fink GR, Guarente L (2004). Saccharomyces cerevisiae SSD1-V confers longevity by a Sir2p-independent mechanism. Genetics.

[bib37] Kaganovich D, Kopito R, Frydman J (2008). Misfolded proteins partition between two distinct quality control compartments. Nature.

[bib38] Kauppila TES, Kauppila JHK, Larsson NG (2017). Mammalian mitochondria and aging: an update. Cell Metabolism.

[bib39] Kurischko C, Kim HK, Kuravi VK, Pratzka J, Luca FC (2011a). The yeast Cbk1 kinase regulates mRNA localization via the mRNA-binding protein Ssd1. The Journal of Cell Biology.

[bib40] Kurischko C, Kuravi VK, Herbert CJ, Luca FC (2011b). Nucleocytoplasmic shuttling of Ssd1 defines the destiny of its bound mRNAs. Molecular Microbiology.

[bib41] Li H, Handsaker B, Wysoker A, Fennell T, Ruan J, Homer N, Marth G, Abecasis G, Durbin R, 1000 Genome Project Data Processing Subgroup (2009). The sequence alignment/Map format and SAMtools. Bioinformatics.

[bib42] Li L, Miles S, Melville Z, Prasad A, Bradley G, Breeden LL (2013). Key events during the transition from rapid growth to quiescence in budding yeast require posttranscriptional regulators. Molecular Biology of the Cell.

[bib43] Li H, Durbin R (2010). Fast and accurate long-read alignment with Burrows-Wheeler transform. Bioinformatics.

[bib44] Ling J, Cho C, Guo LT, Aerni HR, Rinehart J, Söll D (2012). Protein aggregation caused by aminoglycoside action is prevented by a hydrogen peroxide scavenger. Molecular Cell.

[bib45] Liu Y, Borel C, Li L, Müller T, Williams EG, Germain PL, Buljan M, Sajic T, Boersema PJ, Shao W, Faini M, Testa G, Beyer A, Antonarakis SE, Aebersold R (2017). Systematic proteome and proteostasis profiling in human trisomy 21 fibroblast cells. Nature Communications.

[bib46] Liu X, Fu R, Pan Y, Meza-Sosa KF, Zhang Z, Lieberman J (2018). PNPT1 release from mitochondria during apoptosis triggers decay of poly(A) RNAs. Cell.

[bib47] Lubas M, Damgaard CK, Tomecki R, Cysewski D, Jensen TH, Dziembowski A (2013). Exonuclease hDIS3L2 specifies an exosome-independent 3'-5' degradation pathway of human cytoplasmic mRNA. The EMBO Journal.

[bib48] Magtanong L, Ho CH, Barker SL, Jiao W, Baryshnikova A, Bahr S, Smith AM, Heisler LE, Choy JS, Kuzmin E, Andrusiak K, Kobylianski A, Li Z, Costanzo M, Basrai MA, Giaever G, Nislow C, Andrews B, Boone C (2011). Dosage suppression genetic interaction networks enhance functional wiring diagrams of the cell. Nature Biotechnology.

[bib49] Malecki M, Viegas SC, Carneiro T, Golik P, Dressaire C, Ferreira MG, Arraiano CM (2013). The exoribonuclease Dis3L2 defines a novel eukaryotic RNA degradation pathway. The EMBO Journal.

[bib50] McClellan AJ, Scott MD, Frydman J (2005). Folding and quality control of the VHL tumor suppressor proceed through distinct chaperone pathways. Cell.

[bib51] Miles S, Li LH, Melville Z, Breeden LL (2019). Ssd1 and the cell wall integrity pathway promote entry, maintenance, and recovery from quiescence in budding yeast. Molecular Biology of the Cell.

[bib52] Mir SS, Fiedler D, Cashikar AG (2009). Ssd1 is required for thermotolerance and Hsp104-mediated protein disaggregation in Saccharomyces cerevisiae. Molecular and Cellular Biology.

[bib53] Mori F, Tanji K, Miki Y, Toyoshima Y, Sasaki H, Yoshida M, Kakita A, Takahashi H, Wakabayashi K (2018). Immunohistochemical localization of exoribonucleases (DIS3L2 and XRN1) in intranuclear inclusion body disease. Neuroscience Letters.

[bib54] Moriya H, Isono K (1999). Analysis of genetic interactions betweenDHH1,SSD1 andELM1 indicates their involvement in cellular morphology determination inSaccharomyces cerevisiae. Yeast.

[bib55] Morris MR, Astuti D, Maher ER (2013). Perlman syndrome: overgrowth, Wilms tumor predisposition and DIS3L2. Am J Med Genet C Semin Med Genet..

[bib56] Nargund AM, Pellegrino MW, Fiorese CJ, Baker BM, Haynes CM (2012). Mitochondrial import efficiency of ATFS-1 regulates mitochondrial UPR activation. Science.

[bib57] Ni M, Feretzaki M, Li W, Floyd-Averette A, Mieczkowski P, Dietrich FS, Heitman J (2013). Unisexual and heterosexual meiotic reproduction generate aneuploidy and phenotypic diversity de novo in the yeast cryptococcus neoformans. PLOS Biology.

[bib58] Ocampo A, Zambrano A, Barrientos A (2010). Suppression of polyglutamine-induced cytotoxicity in *Saccharomyces cerevisiae* by enhancement of mitochondrial biogenesis. The FASEB Journal.

[bib59] Ohyama Y, Kasahara K, Kokubo T (2010). Saccharomyces cerevisiae Ssd1p promotes CLN2 expression by binding to the 5'-untranslated region of CLN2 mRNA. Genes to Cells.

[bib60] Oromendia AB, Dodgson SE, Amon A (2012). Aneuploidy causes proteotoxic stress in yeast. Genes & Development.

[bib61] Oromendia AB, Amon A (2014). Aneuploidy: implications for protein homeostasis and disease. Disease Models & Mechanisms.

[bib62] Pavelka N, Rancati G, Zhu J, Bradford WD, Saraf A, Florens L, Sanderson BW, Hattem GL, Li R (2010). Aneuploidy confers quantitative proteome changes and phenotypic variation in budding yeast. Nature.

[bib63] Pavelka N, Rancati G (2013). Never in neutral: a systems biology and evolutionary perspective on how Aneuploidy contributes to human diseases. Cytogenetic and Genome Research.

[bib64] Peter J, De Chiara M, Friedrich A, Yue J-X, Pflieger D, Bergström A, Sigwalt A, Barre B, Freel K, Llored A, Cruaud C, Labadie K, Aury J-M, Istace B, Lebrigand K, Barbry P, Engelen S, Lemainque A, Wincker P, Liti G, Schacherer J (2018). Genome evolution across 1,011 Saccharomyces cerevisiae isolates. Nature.

[bib65] Qureshi MA, Haynes CM, Pellegrino MW (2017). The mitochondrial unfolded protein response: Signaling from the powerhouse. Journal of Biological Chemistry.

[bib66] Rancati G, Pavelka N, Fleharty B, Noll A, Trimble R, Walton K, Perera A, Staehling-Hampton K, Seidel CW, Li R (2008). Aneuploidy underlies rapid adaptive evolution of yeast cells deprived of a conserved cytokinesis motor. Cell.

[bib67] Robinson MD, McCarthy DJ, Smyth GK (2010). edgeR: a Bioconductor package for differential expression analysis of digital gene expression data. Bioinformatics.

[bib68] Ruan L, Zhou C, Jin E, Kucharavy A, Zhang Y, Wen Z, Florens L, Li R (2017). Cytosolic proteostasis through importing of misfolded proteins into mitochondria. Nature.

[bib69] Saldanha AJ (2004). Java Treeview--extensible visualization of microarray data. Bioinformatics.

[bib70] Santaguida S, Vasile E, White E, Amon A (2015). Aneuploidy-induced cellular stresses limit autophagic degradation. Genes & Development.

[bib71] Sardi M, Paithane V, Place M, Robinson DE, Hose J, Wohlbach DJ, Gasch AP (2018). Genome-wide association across Saccharomyces cerevisiae strains reveals substantial variation in underlying gene requirements for toxin tolerance. PLOS Genetics.

[bib72] Schindelin J, Arganda-Carreras I, Frise E, Kaynig V, Longair M, Pietzsch T, Preibisch S, Rueden C, Saalfeld S, Schmid B, Tinevez J-Y, White DJ, Hartenstein V, Eliceiri K, Tomancak P, Cardona A (2012). Fiji: an open-source platform for biological-image analysis. Nature Methods.

[bib73] Scorrano L, De Matteis MA, Emr S, Giordano F, Hajnóczky G, Kornmann B, Lackner LL, Levine TP, Pellegrini L, Reinisch K, Rizzuto R, Simmen T, Stenmark H, Ungermann C, Schuldiner M (2019). Coming together to define membrane contact sites. Nature Communications.

[bib74] Selmecki A (2006). Aneuploidy and isochromosome formation in Drug-Resistant candida albicans. Science.

[bib75] Selmecki AM, Dulmage K, Cowen LE, Anderson JB, Berman J (2009). Acquisition of aneuploidy provides increased fitness during the evolution of antifungal drug resistance. PLOS Genetics.

[bib76] Sheltzer JM, Torres EM, Dunham MJ, Amon A (2012). Transcriptional consequences of aneuploidy. PNAS.

[bib77] Shishkova E, Hebert AS, Westphall MS, Coon JJ (2018). Ultra-High pressure (>30,000 psi) Packing of capillary columns enhancing depth of shotgun proteomic analyses. Analytical Chemistry.

[bib78] Simillion C, Liechti R, Lischer HEL, Ioannidis V, Bruggmann R (2017). Avoiding the pitfalls of gene set enrichment analysis with SetRank. BMC Bioinformatics.

[bib79] Stingele S, Stoehr G, Peplowska K, Cox J, Mann M, Storchova Z (2012). Global analysis of genome, transcriptome and proteome reveals the response to aneuploidy in human cells. Molecular Systems Biology.

[bib80] Stingele S, Stoehr G, Storchova Z (2013). Activation of autophagy in cells with abnormal karyotype. Autophagy.

[bib81] Sutton A, Immanuel D, Arndt KT (1991). The SIT4 protein phosphatase functions in late G1 for progression into S phase. Molecular and Cellular Biology.

[bib82] Tang Y-C, Williams BR, Siegel JJ, Amon A (2011). Identification of Aneuploidy-Selective antiproliferation compounds. Cell.

[bib83] Targa A, Rancati G (2018). Cancer: a CINful evolution. Current Opinion in Cell Biology.

[bib84] Thomas MP, Liu X, Whangbo J, McCrossan G, Sanborn KB, Basar E, Walch M, Lieberman J (2015). Apoptosis Triggers Specific, Rapid, and Global mRNA Decay with 3′ Uridylated Intermediates Degraded by DIS3L2. Cell Reports.

[bib85] Thorburn RR, Gonzalez C, Brar GA, Christen S, Carlile TM, Ingolia NT, Sauer U, Weissman JS, Amon A (2013). Aneuploid yeast strains exhibit defects in cell growth and passage through START. Molecular Biology of the Cell.

[bib86] Torres EM, Sokolsky T, Tucker CM, Chan LY, Boselli M, Dunham MJ, Amon A (2007). Effects of aneuploidy on cellular physiology and cell division in haploid yeast. Science.

[bib87] Torres EM, Dephoure N, Panneerselvam A, Tucker CM, Whittaker CA, Gygi SP, Dunham MJ, Amon A (2010). Identification of aneuploidy-tolerating mutations. Cell.

[bib88] Travers KJ, Patil CK, Wodicka L, Lockhart DJ, Weissman JS, Walter P (2000). Functional and genomic analyses reveal an essential coordination between the unfolded protein response and ER-associated degradation. Cell.

[bib89] Tsai H-J, Nelliat AR, Choudhury MI, Kucharavy A, Bradford WD, Cook ME, Kim J, Mair DB, Sun SX, Schatz MC, Li R (2019). Hypo-osmotic-like stress underlies general cellular defects of aneuploidy. Nature.

[bib90] Uesono Y, Fujita A, Toh-e A, Kikuchi Y (1994). The MCS1/SSD1/SRK1/SSL1 gene is involved in stable maintenance of the chromosome in yeast. Gene.

[bib91] Uesono Y, Toh-e A, Kikuchi Y (1997). Ssd1p of *Saccharomyces cerevisiae* Associates with RNA. Journal of Biological Chemistry.

[bib92] Ustianenko D, Hrossova D, Potesil D, Chalupnikova K, Hrazdilova K, Pachernik J, Cetkovska K, Uldrijan S, Zdrahal Z, Vanacova S (2013). Mammalian DIS3L2 exoribonuclease targets the uridylated precursors of let-7 miRNAs. RNA.

[bib93] Viegas SC, Silva IJ, Apura P, Matos RG, Arraiano CM (2015). Surprises in the 3'-end: 'U' can decide too!. FEBS Journal.

[bib94] Wang X, Chen XJ (2015). A cytosolic network suppressing mitochondria-mediated proteostatic stress and cell death. Nature.

[bib95] Wanless AG, Lin Y, Weiss EL (2014). Cell morphogenesis proteins are translationally controlled through UTRs by the ndr/LATS target Ssd1. PLOS ONE.

[bib96] Weidberg H, Amon A (2018). MitoCPR-A surveillance pathway that protects mitochondria in response to protein import stress. Science.

[bib97] Wertheimer NB, Stone N, Berman J (2016). Ploidy dynamics and evolvability in fungi. Philosophical Transactions of the Royal Society B: Biological Sciences.

[bib98] Wrobel L, Topf U, Bragoszewski P, Wiese S, Sztolsztener ME, Oeljeklaus S, Varabyova A, Lirski M, Chroscicki P, Mroczek S, Januszewicz E, Dziembowski A, Koblowska M, Warscheid B, Chacinska A (2015). Mistargeted mitochondrial proteins activate a proteostatic response in the cytosol. Nature.

[bib99] Yona AH, Manor YS, Herbst RH, Romano GH, Mitchell A, Kupiec M, Pilpel Y, Dahan O (2012). Chromosomal duplication is a transient evolutionary solution to stress. PNAS.

[bib100] Zhou C, Slaughter BD, Unruh JR, Guo F, Yu Z, Mickey K, Narkar A, Ross RT, McClain M, Li R (2014). Organelle-based aggregation and retention of damaged proteins in asymmetrically dividing cells. Cell.

