# Supplementary figures and images for: The genetic basis of aneuploidy tolerance in wild yeast
Source: eLife. 2020 Jan 7;9:e52063. doi: 10.7554/eLife.52063 (PMC6970514; doi:10.7554/eLife.52063)

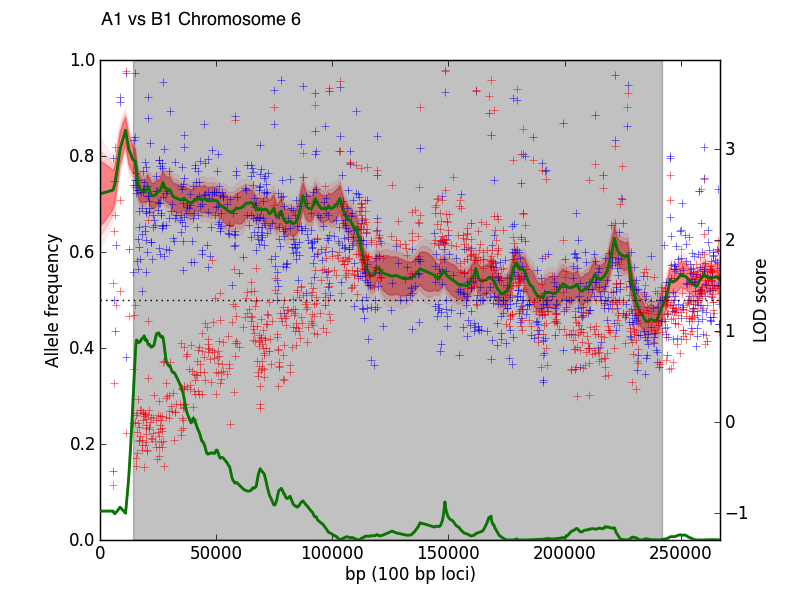

Supplement: Figure 1—source data 1. [file elife-52063-fig1-data1.zip › A1vB1/A1vB1_n20_ch6.png]

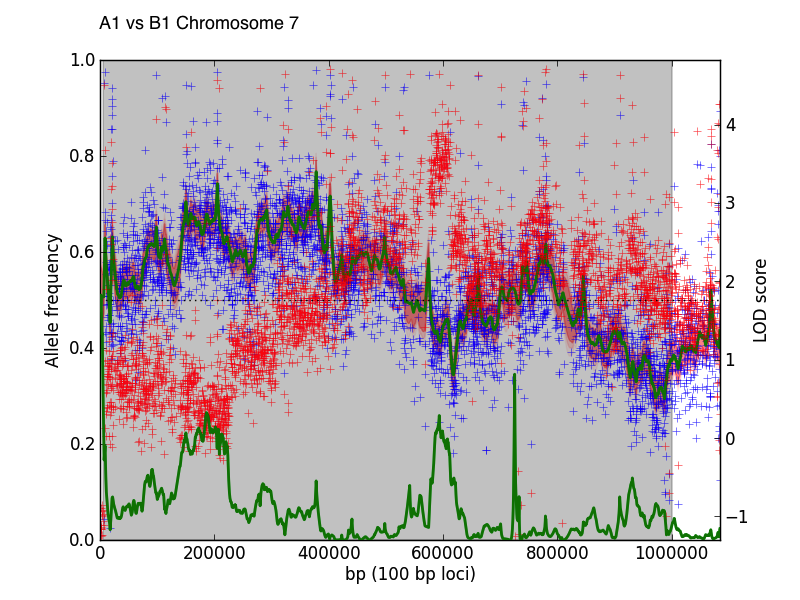

Supplement: Figure 1—source data 1. [file elife-52063-fig1-data1.zip › A1vB1/A1vB1_n20_ch7.png]

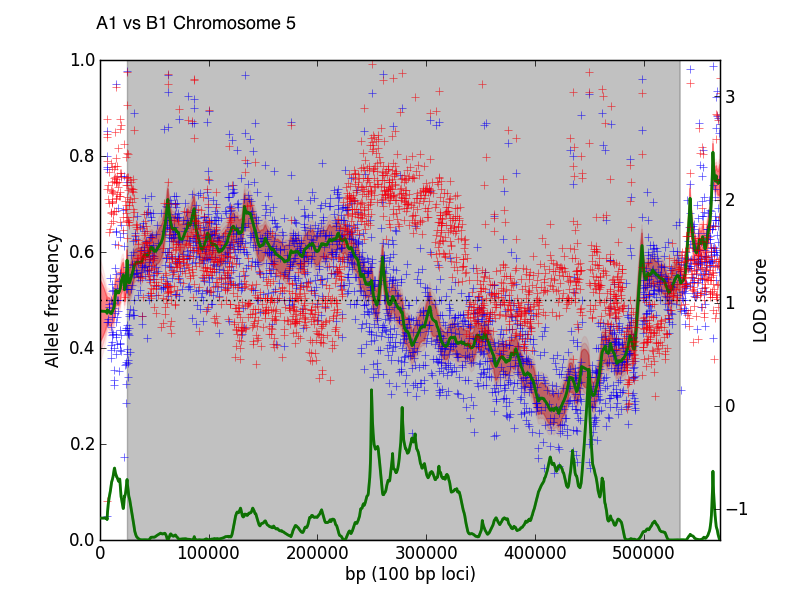

Supplement: Figure 1—source data 1. [file elife-52063-fig1-data1.zip › A1vB1/A1vB1_n20_ch5.png]

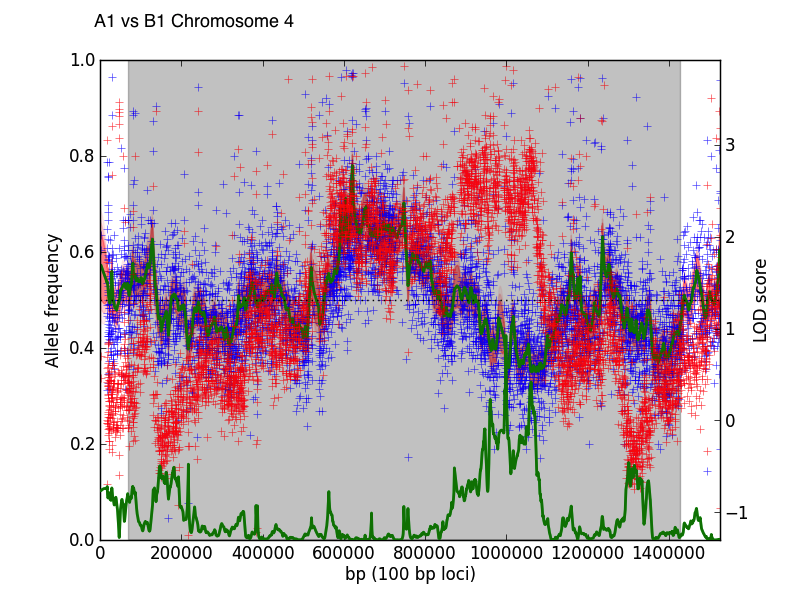

Supplement: Figure 1—source data 1. [file elife-52063-fig1-data1.zip › A1vB1/A1vB1_n20_ch4.png]

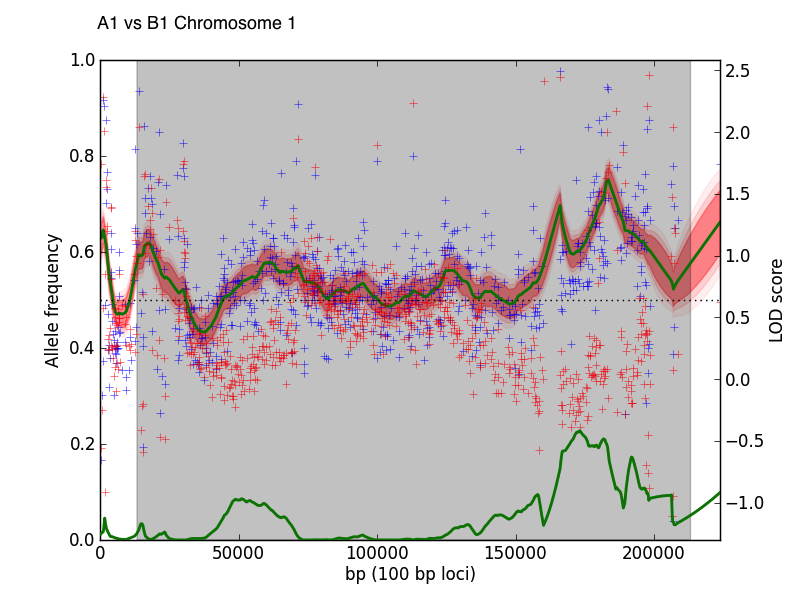

Supplement: Figure 1—source data 1. [file elife-52063-fig1-data1.zip › A1vB1/A1vB1_n20_ch1.png]

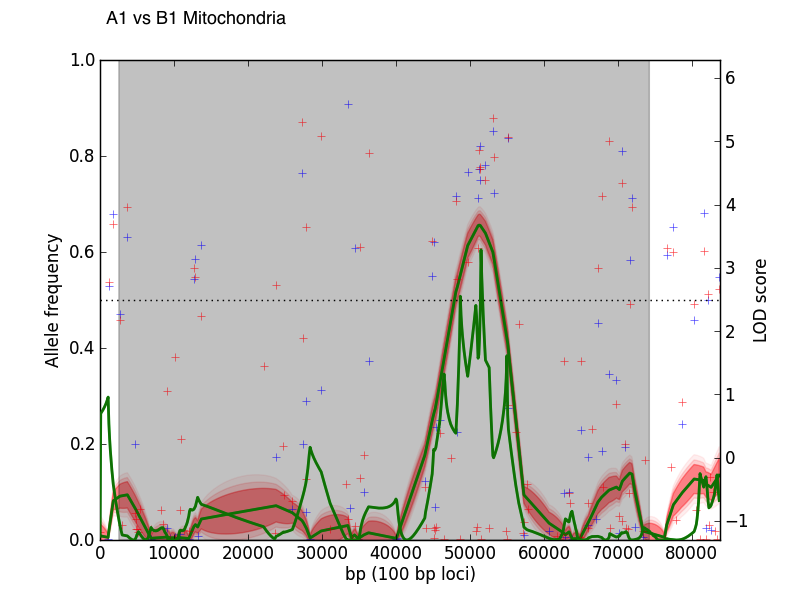

Supplement: Figure 1—source data 1. [file elife-52063-fig1-data1.zip › A1vB1/A1vB1_n20_mito.png]

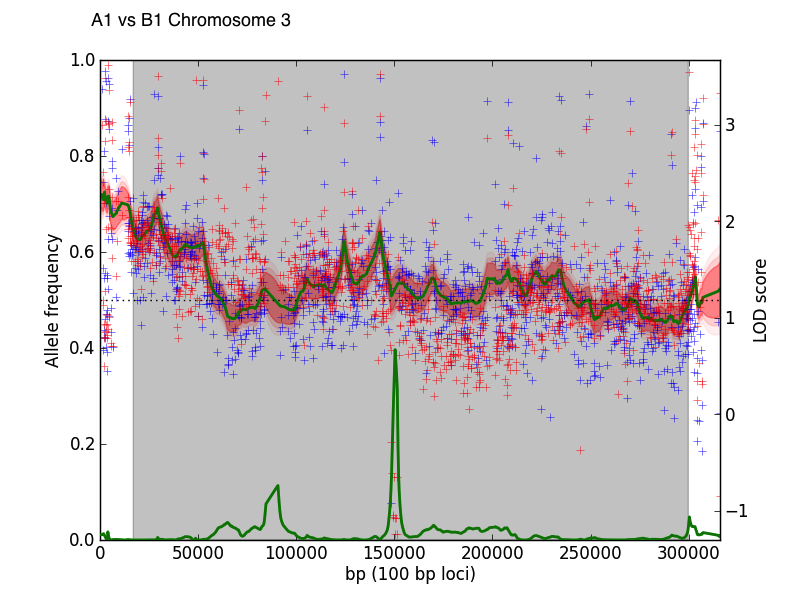

Supplement: Figure 1—source data 1. [file elife-52063-fig1-data1.zip › A1vB1/A1vB1_n20_ch3.png]

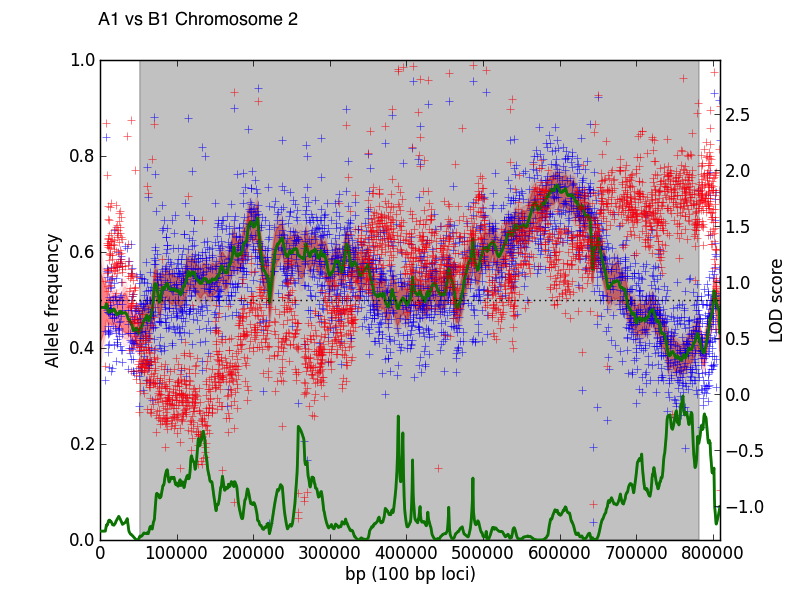

Supplement: Figure 1—source data 1. [file elife-52063-fig1-data1.zip › A1vB1/A1vB1_n20_ch2.png]

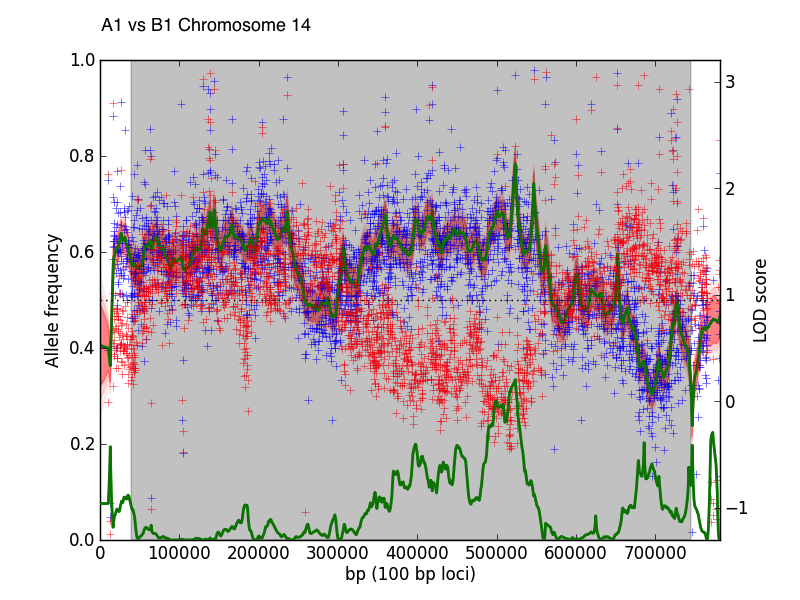

Supplement: Figure 1—source data 1. [file elife-52063-fig1-data1.zip › A1vB1/A1vB1_n20_ch14.png]

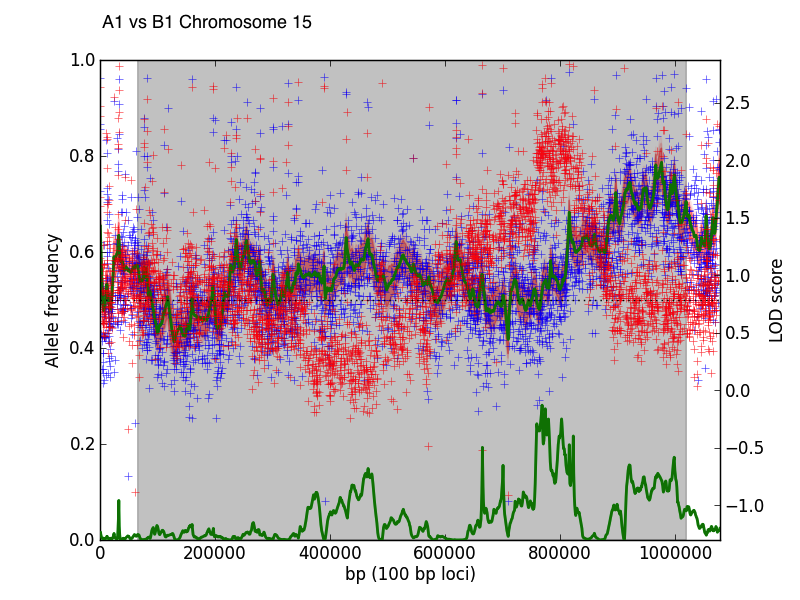

Supplement: Figure 1—source data 1. [file elife-52063-fig1-data1.zip › A1vB1/A1vB1_n20_ch15.png]

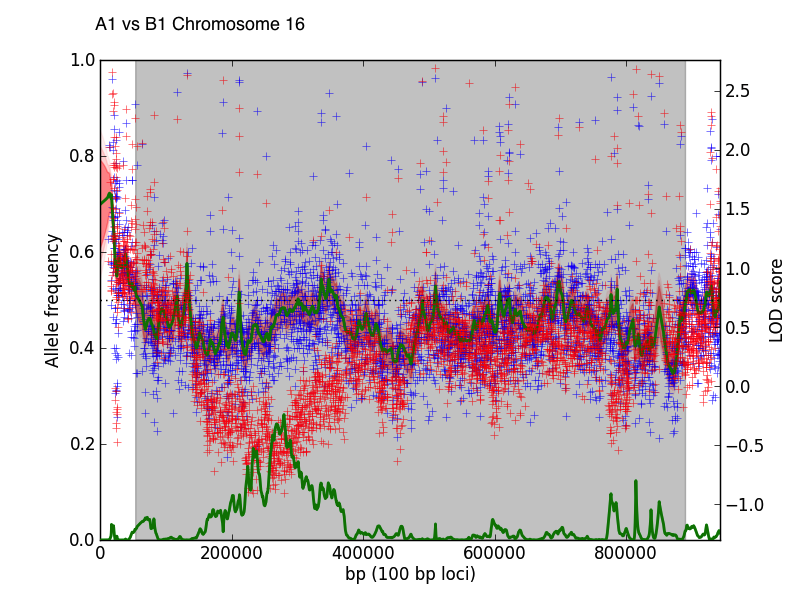

Supplement: Figure 1—source data 1. [file elife-52063-fig1-data1.zip › A1vB1/A1vB1_n20_ch16.png]

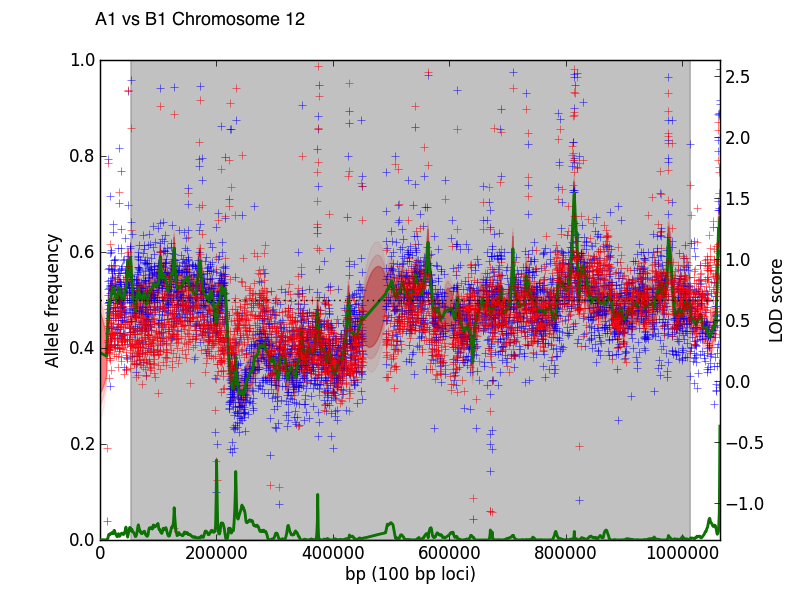

Supplement: Figure 1—source data 1. [file elife-52063-fig1-data1.zip › A1vB1/A1vB1_n20_ch12.png]

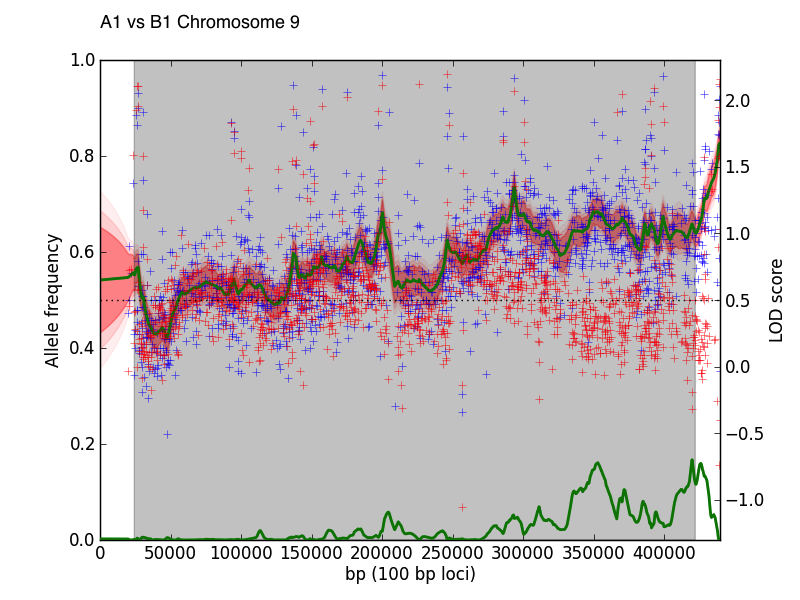

Supplement: Figure 1—source data 1. [file elife-52063-fig1-data1.zip › A1vB1/A1vB1_n20_ch9.png]

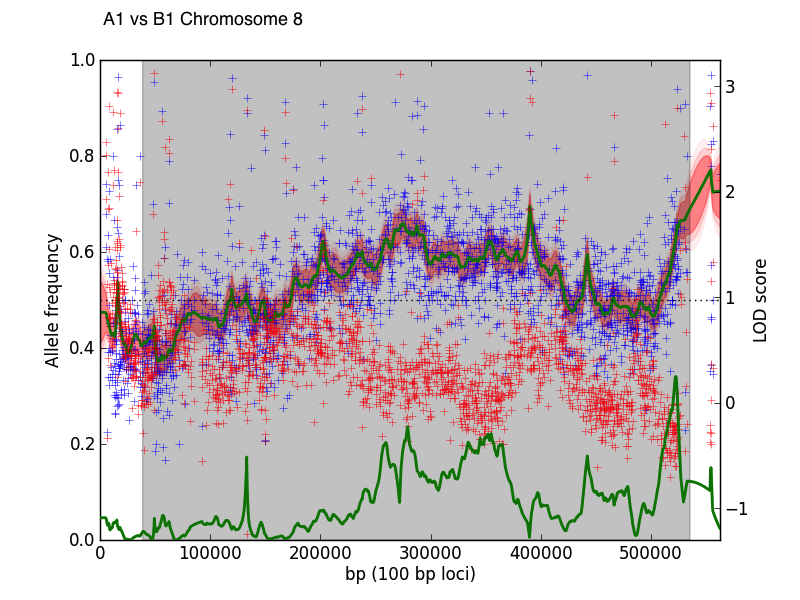

Supplement: Figure 1—source data 1. [file elife-52063-fig1-data1.zip › A1vB1/A1vB1_n20_ch8.png]

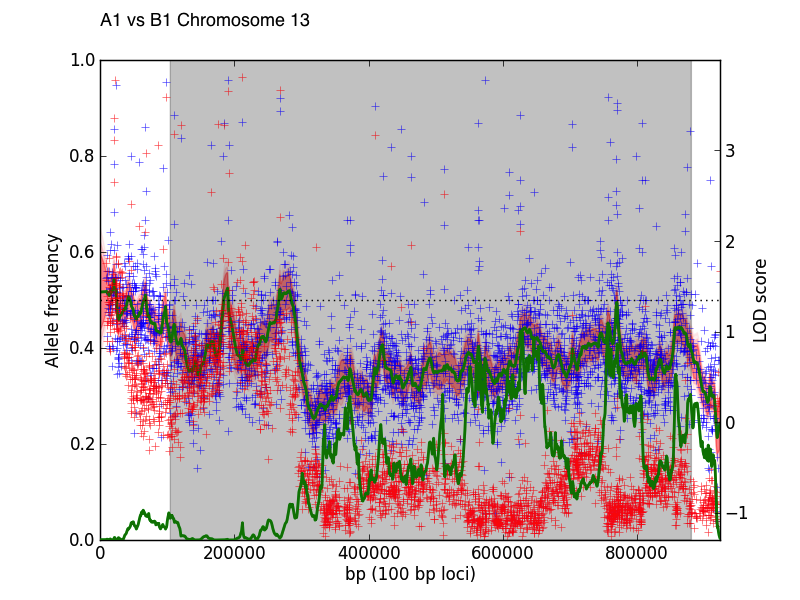

Supplement: Figure 1—source data 1. [file elife-52063-fig1-data1.zip › A1vB1/A1vB1_n20_ch13.png]

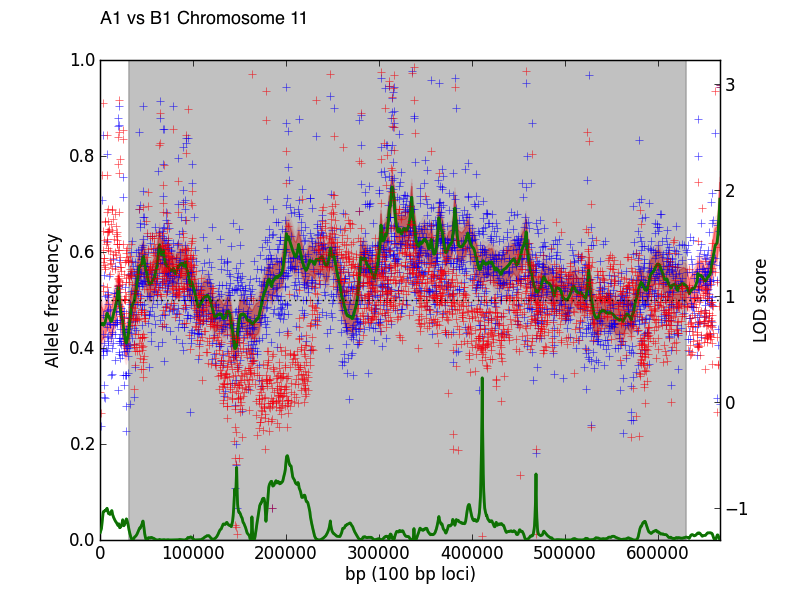

Supplement: Figure 1—source data 1. [file elife-52063-fig1-data1.zip › A1vB1/A1vB1_n20_ch11.png]

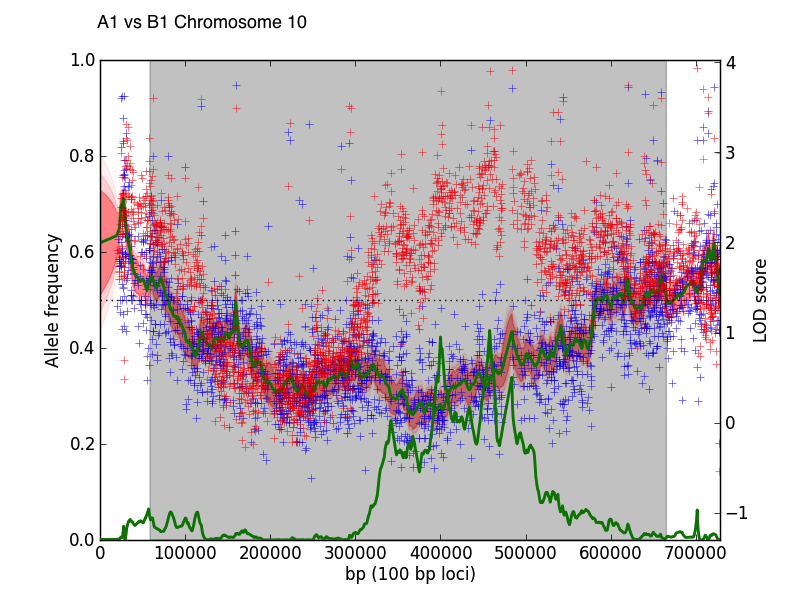

Supplement: Figure 1—source data 1. [file elife-52063-fig1-data1.zip › A1vB1/A1vB1_n20_ch10.png]

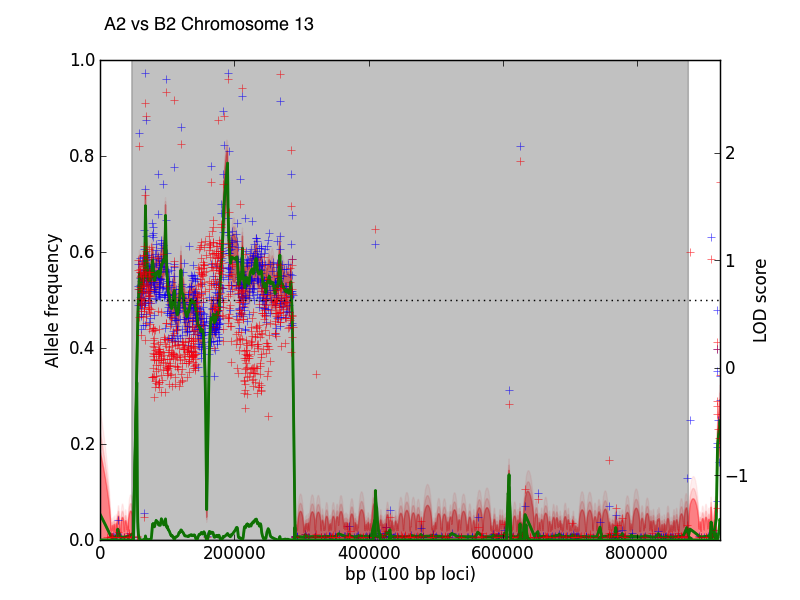

Supplement: Figure 1—source data 1. [file elife-52063-fig1-data1.zip › A2vB2/A2vB2_n10_ch13.png]

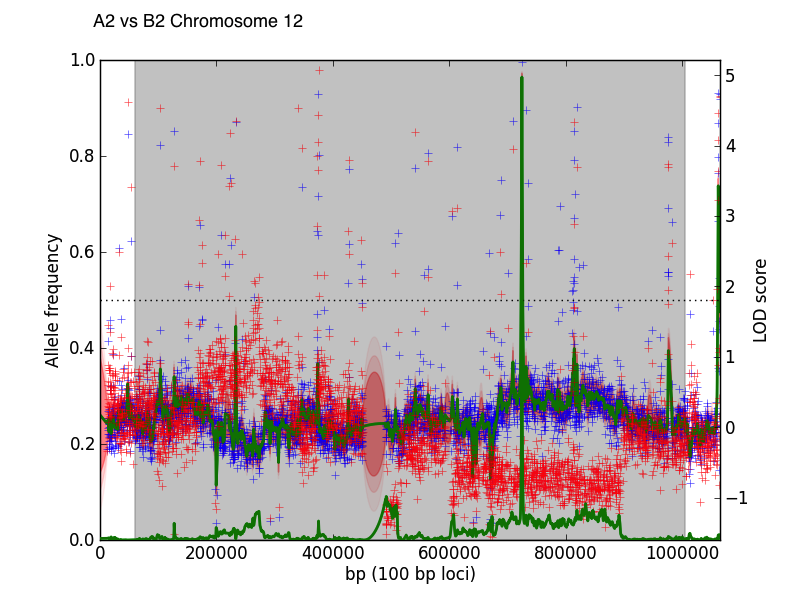

Supplement: Figure 1—source data 1. [file elife-52063-fig1-data1.zip › A2vB2/A2vB2_n10_ch12.png]

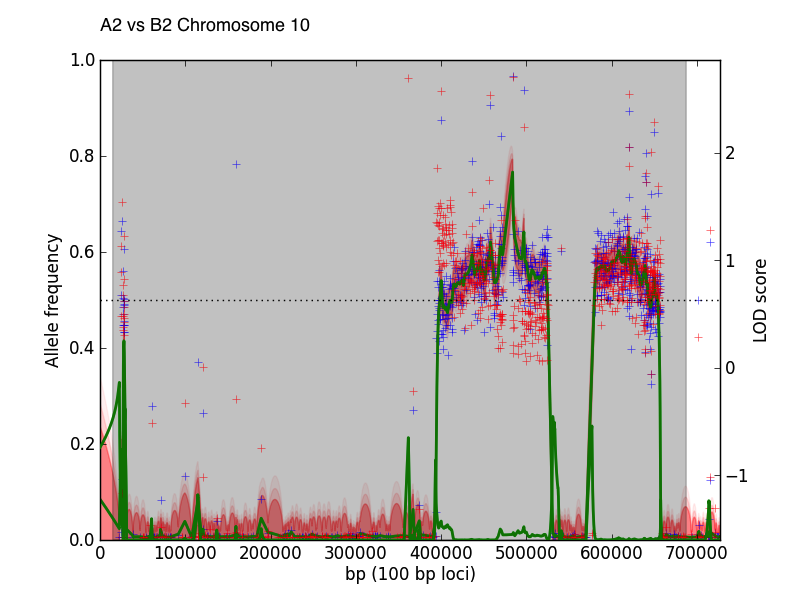

Supplement: Figure 1—source data 1. [file elife-52063-fig1-data1.zip › A2vB2/A2vB2_n10_ch10.png]

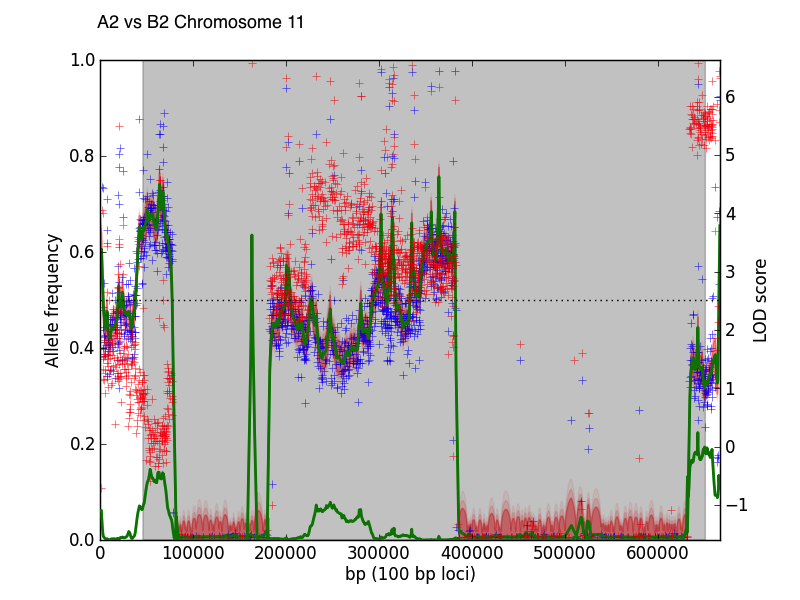

Supplement: Figure 1—source data 1. [file elife-52063-fig1-data1.zip › A2vB2/A2vB2_n10_ch11.png]

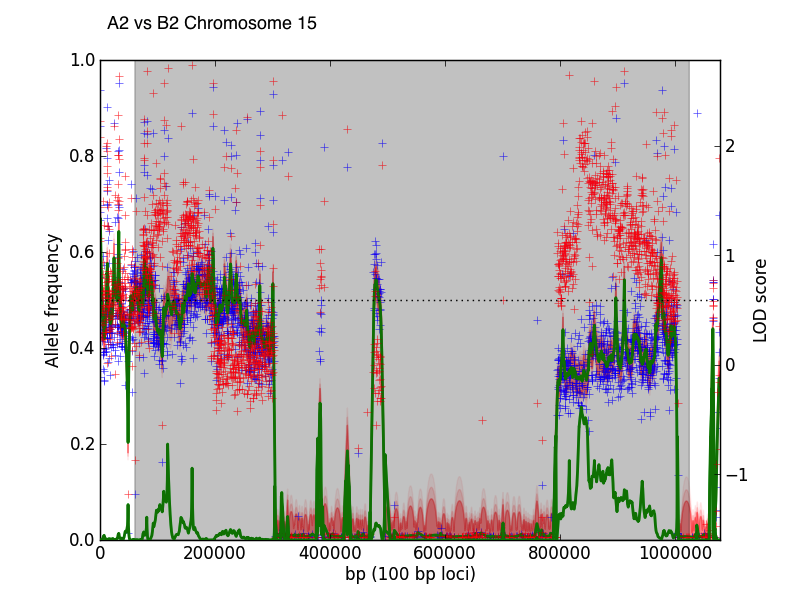

Supplement: Figure 1—source data 1. [file elife-52063-fig1-data1.zip › A2vB2/A2vB2_n10_ch15.png]

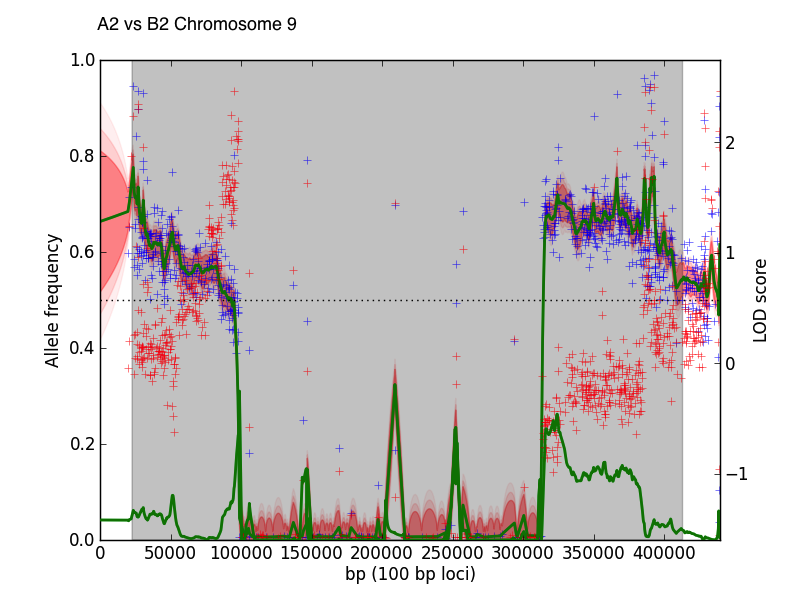

Supplement: Figure 1—source data 1. [file elife-52063-fig1-data1.zip › A2vB2/A2vB2_n10_ch9.png]

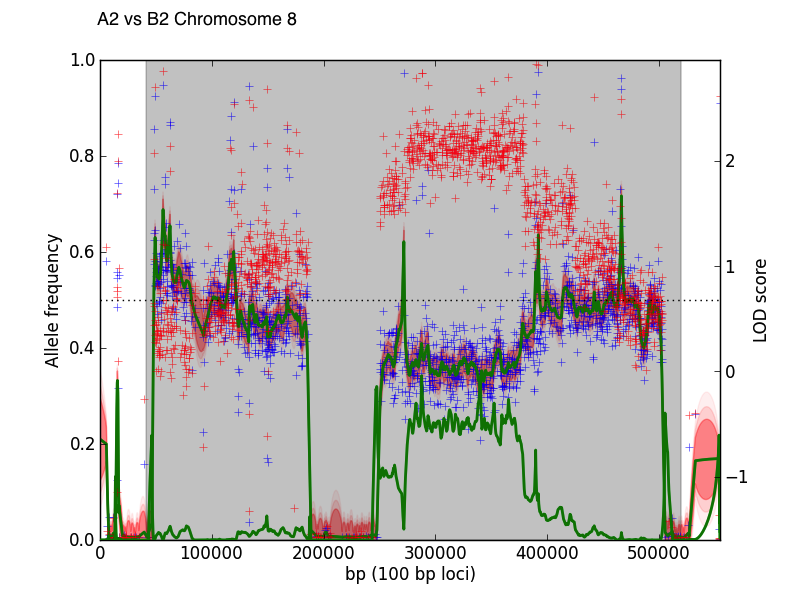

Supplement: Figure 1—source data 1. [file elife-52063-fig1-data1.zip › A2vB2/A2vB2_n10_ch8.png]

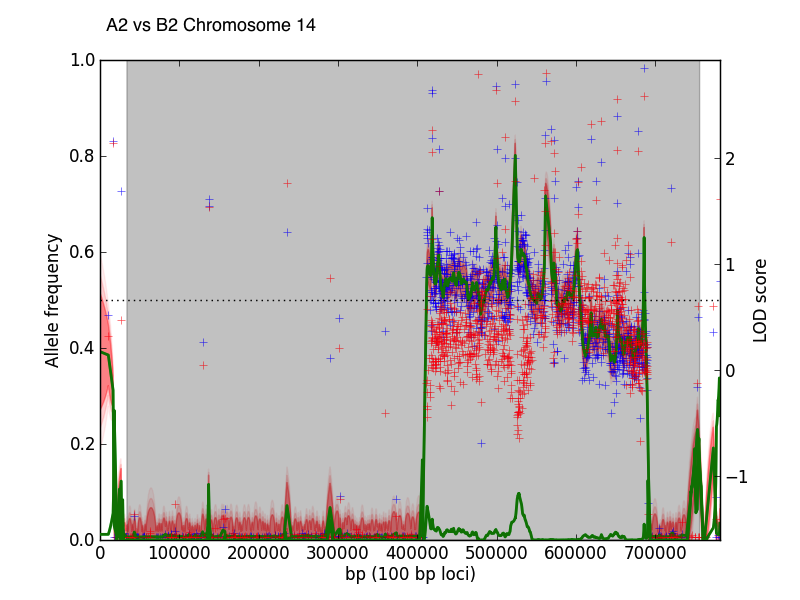

Supplement: Figure 1—source data 1. [file elife-52063-fig1-data1.zip › A2vB2/A2vB2_n10_ch14.png]

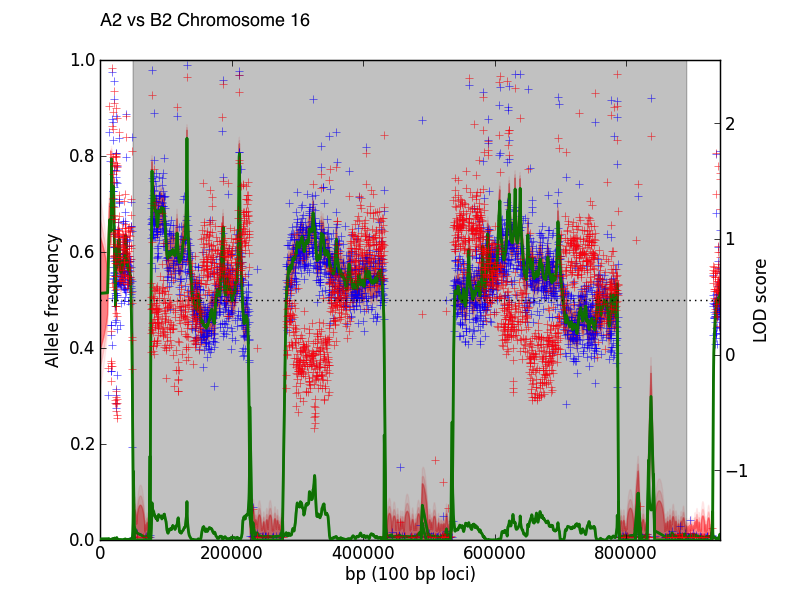

Supplement: Figure 1—source data 1. [file elife-52063-fig1-data1.zip › A2vB2/A2vB2_n10_ch16.png]

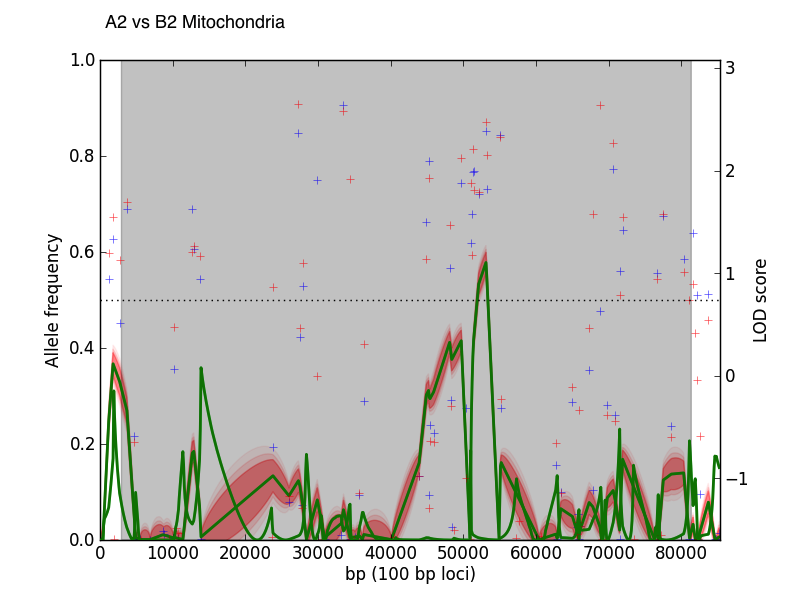

Supplement: Figure 1—source data 1. [file elife-52063-fig1-data1.zip › A2vB2/A2vB2_n10_mito.png]

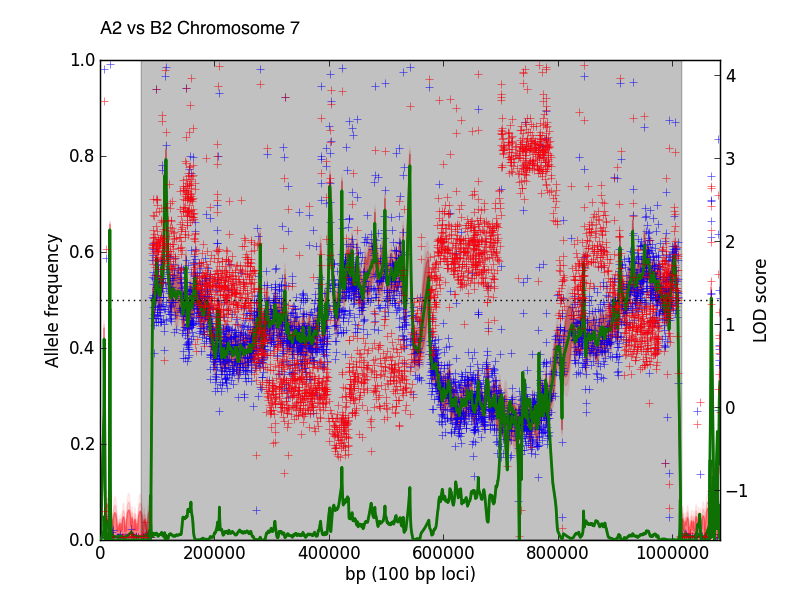

Supplement: Figure 1—source data 1. [file elife-52063-fig1-data1.zip › A2vB2/A2vB2_n10_ch7.png]

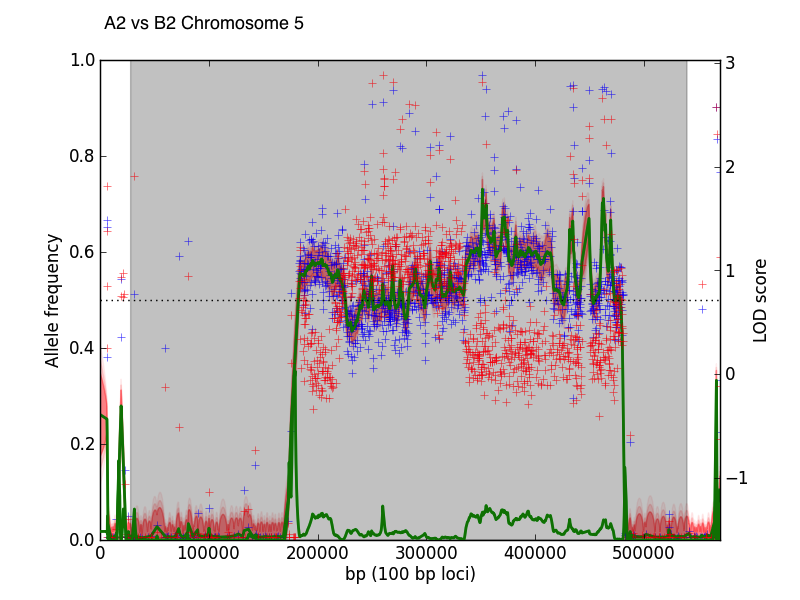

Supplement: Figure 1—source data 1. [file elife-52063-fig1-data1.zip › A2vB2/A2vB2_n10_ch5.png]

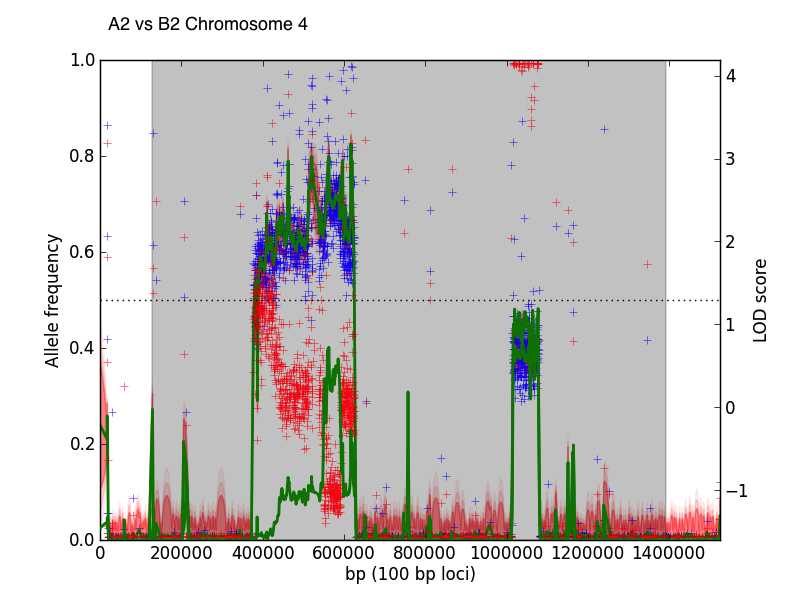

Supplement: Figure 1—source data 1. [file elife-52063-fig1-data1.zip › A2vB2/A2vB2_n10_ch4.png]

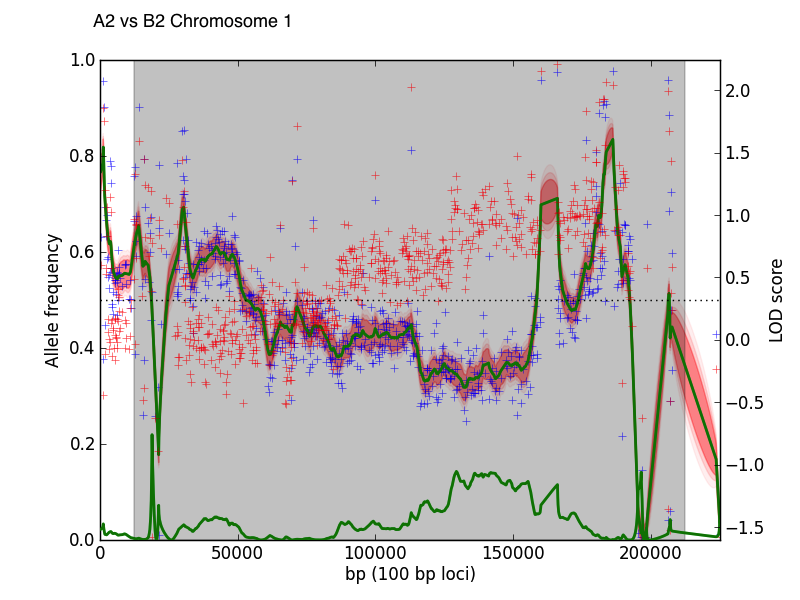

Supplement: Figure 1—source data 1. [file elife-52063-fig1-data1.zip › A2vB2/A2vB2_n10_ch1.png]

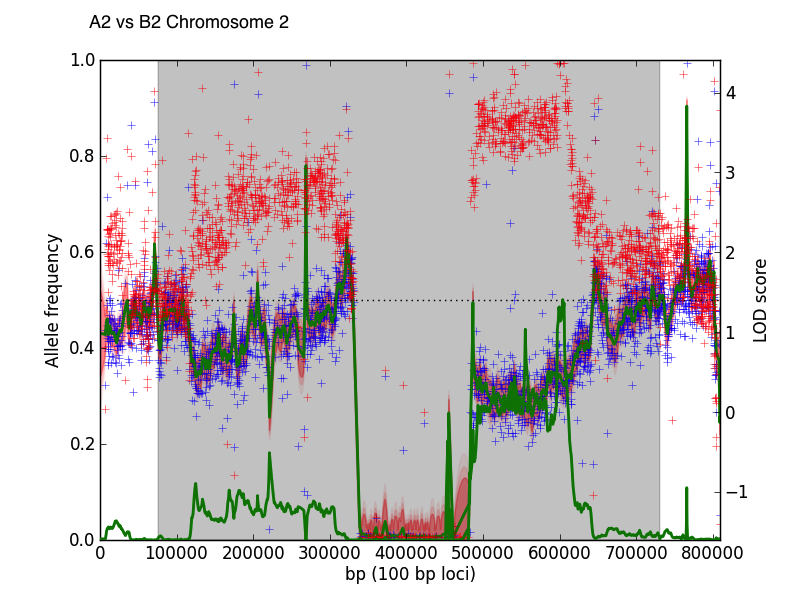

Supplement: Figure 1—source data 1. [file elife-52063-fig1-data1.zip › A2vB2/A2vB2_n10_ch2.png]

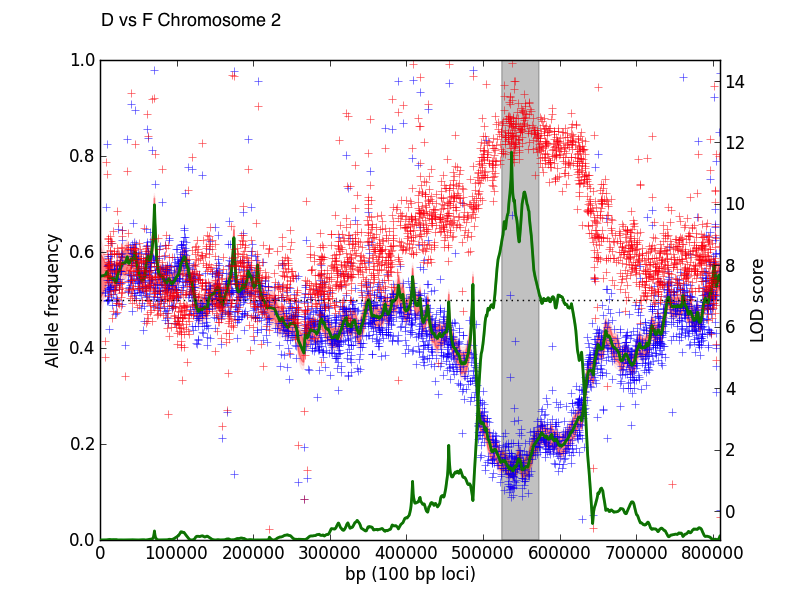

Supplement: Figure 1—source data 1. [file elife-52063-fig1-data1.zip › DvF/DvF_n46_ch2.png]

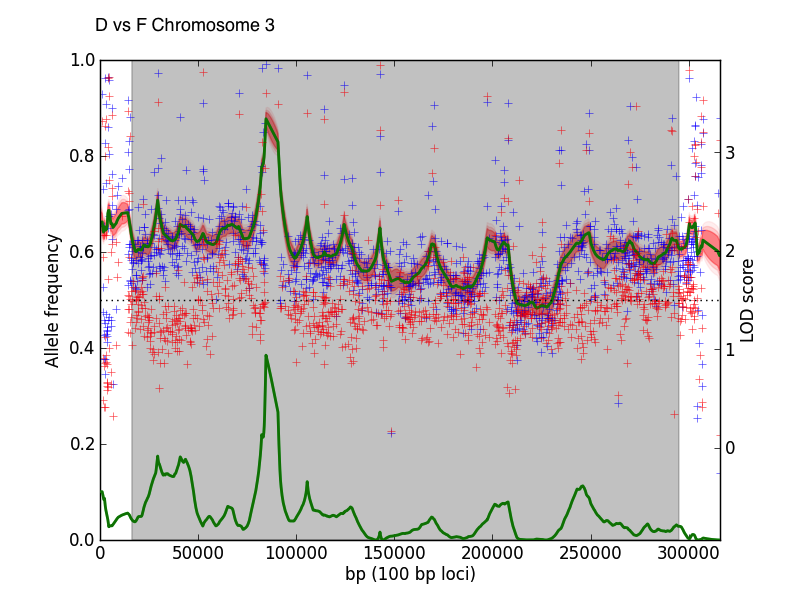

Supplement: Figure 1—source data 1. [file elife-52063-fig1-data1.zip › DvF/DvF_n46_ch3.png]

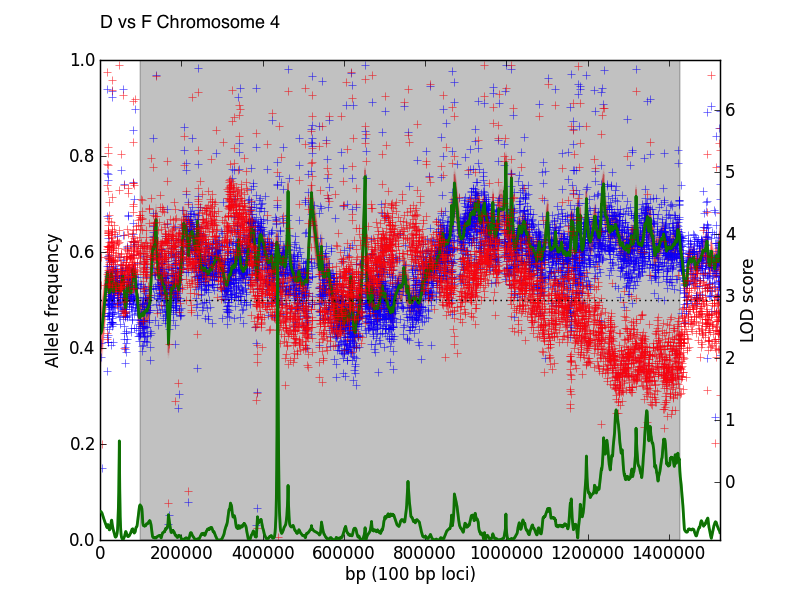

Supplement: Figure 1—source data 1. [file elife-52063-fig1-data1.zip › DvF/DvF_n46_ch4.png]

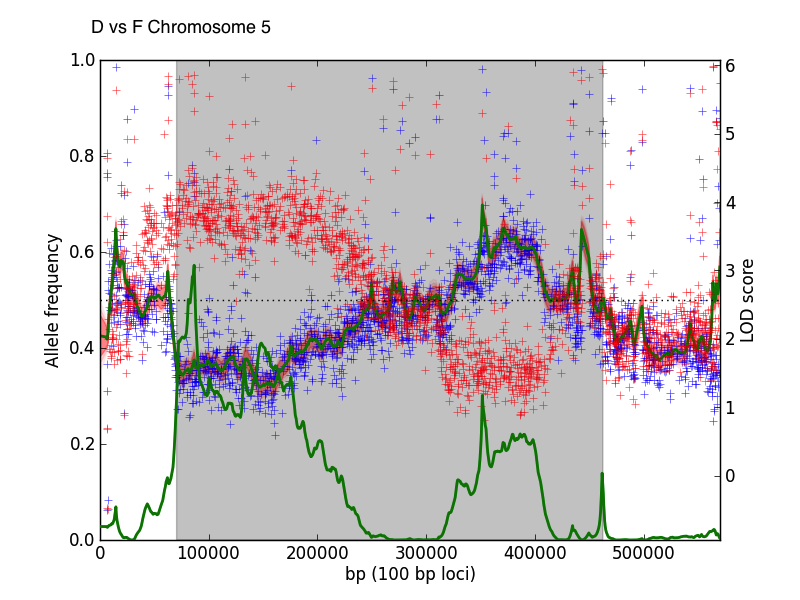

Supplement: Figure 1—source data 1. [file elife-52063-fig1-data1.zip › DvF/DvF_n46_ch5.png]

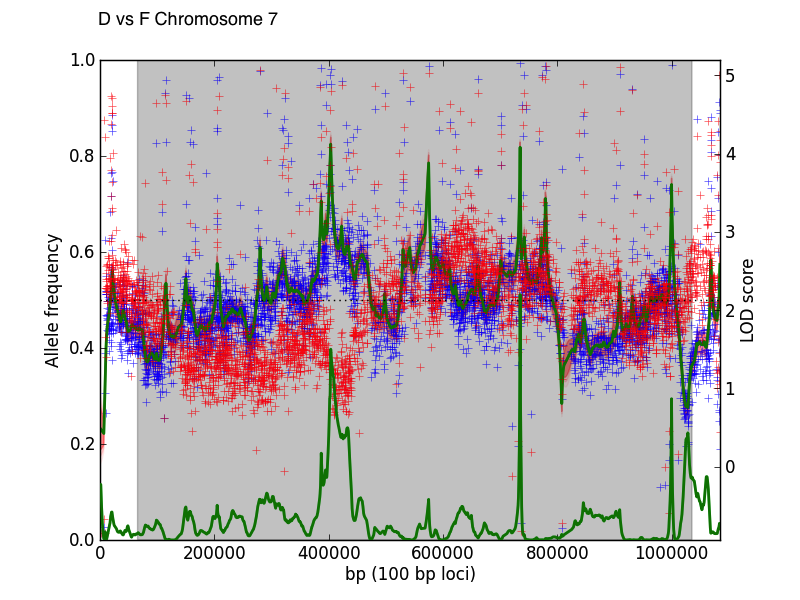

Supplement: Figure 1—source data 1. [file elife-52063-fig1-data1.zip › DvF/DvF_n46_ch7.png]

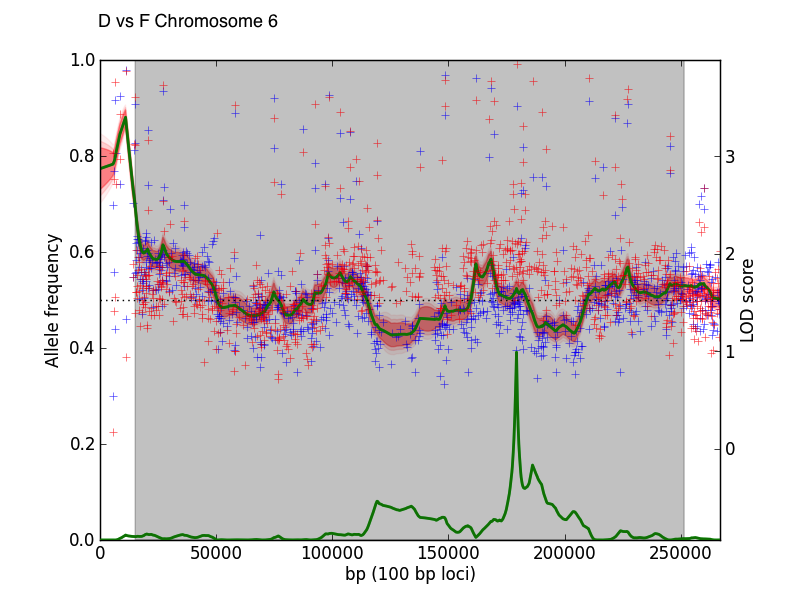

Supplement: Figure 1—source data 1. [file elife-52063-fig1-data1.zip › DvF/DvF_n46_ch6.png]

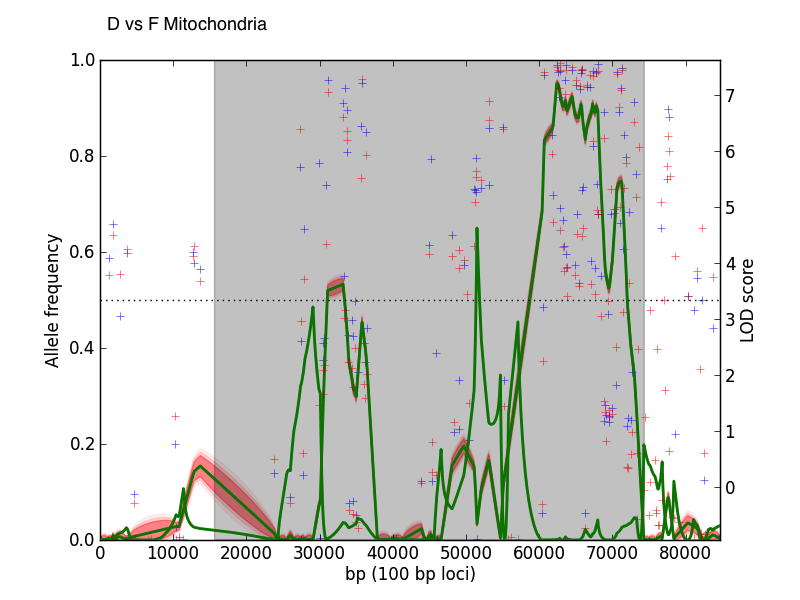

Supplement: Figure 1—source data 1. [file elife-52063-fig1-data1.zip › DvF/DvF_n46_mito.png]

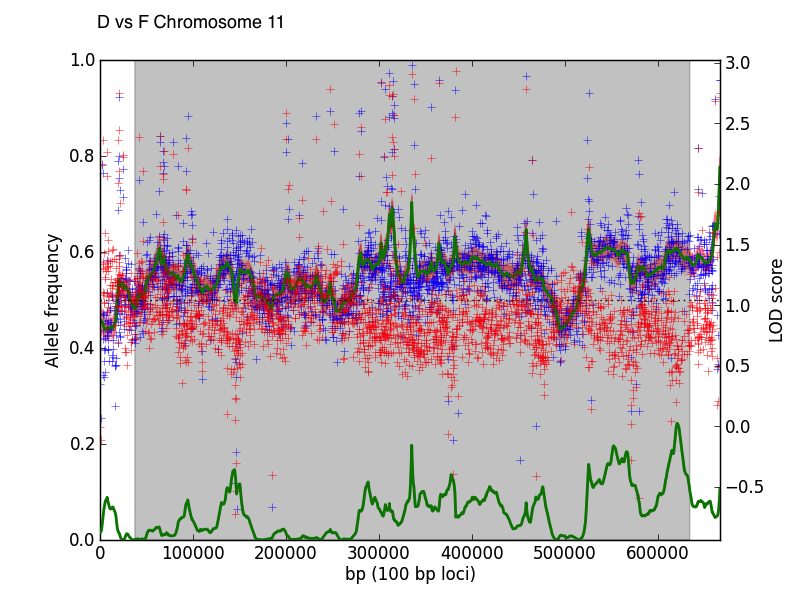

Supplement: Figure 1—source data 1. [file elife-52063-fig1-data1.zip › DvF/DvF_n46_ch11.png]

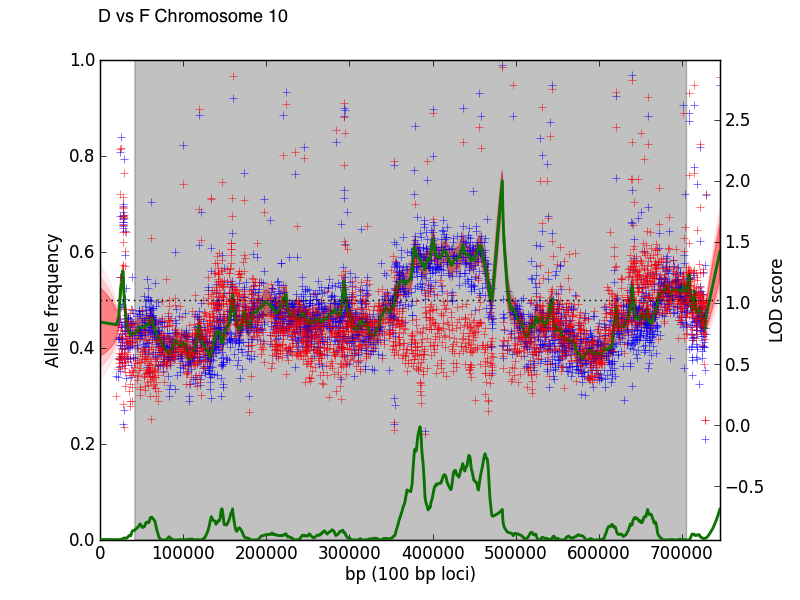

Supplement: Figure 1—source data 1. [file elife-52063-fig1-data1.zip › DvF/DvF_n46_ch10.png]

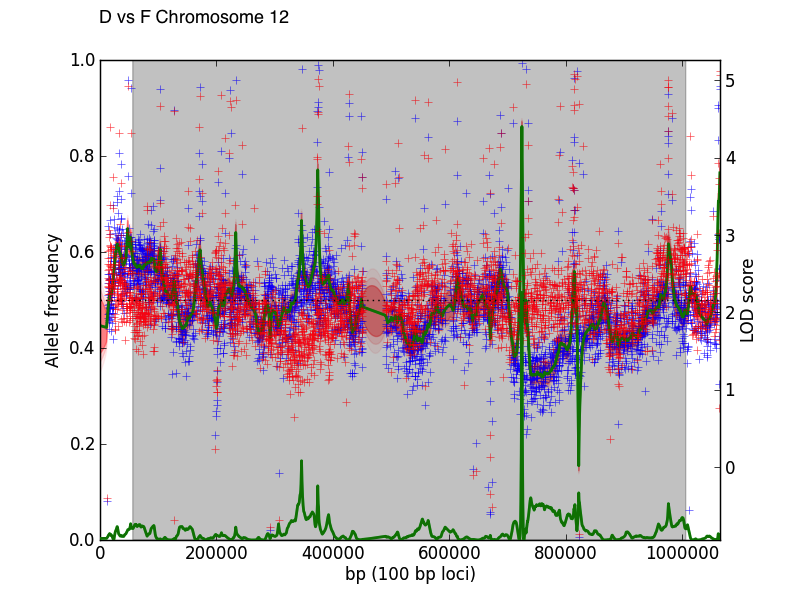

Supplement: Figure 1—source data 1. [file elife-52063-fig1-data1.zip › DvF/DvF_n46_ch12.png]

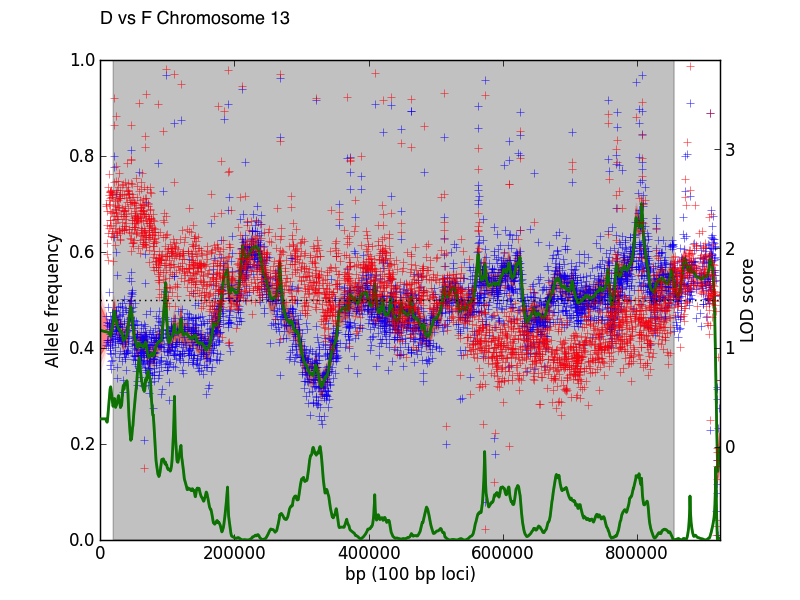

Supplement: Figure 1—source data 1. [file elife-52063-fig1-data1.zip › DvF/DvF_n46_ch13.png]

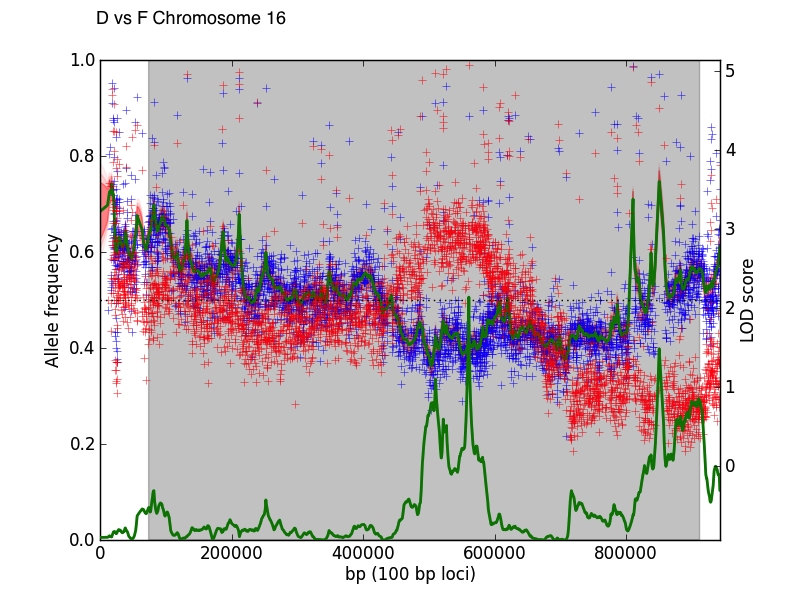

Supplement: Figure 1—source data 1. [file elife-52063-fig1-data1.zip › DvF/DvF_n46_ch16.png]

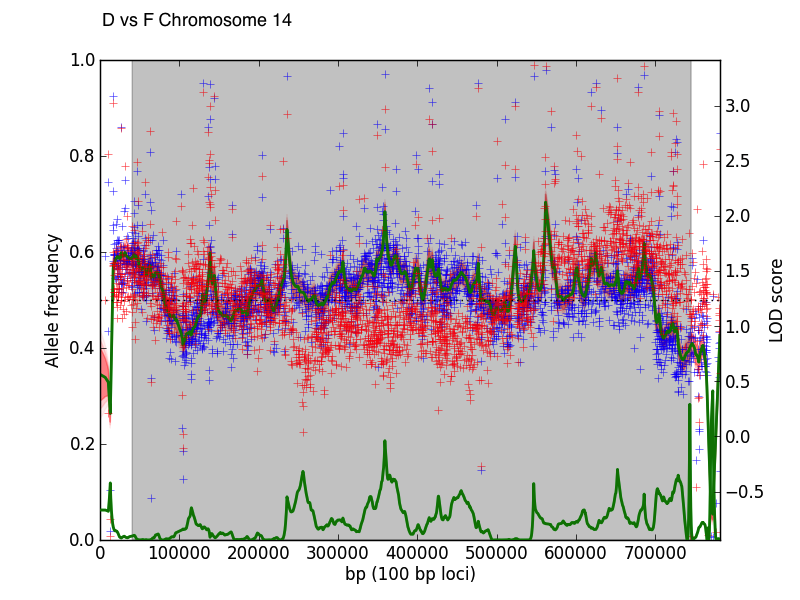

Supplement: Figure 1—source data 1. [file elife-52063-fig1-data1.zip › DvF/DvF_n46_ch14.png]

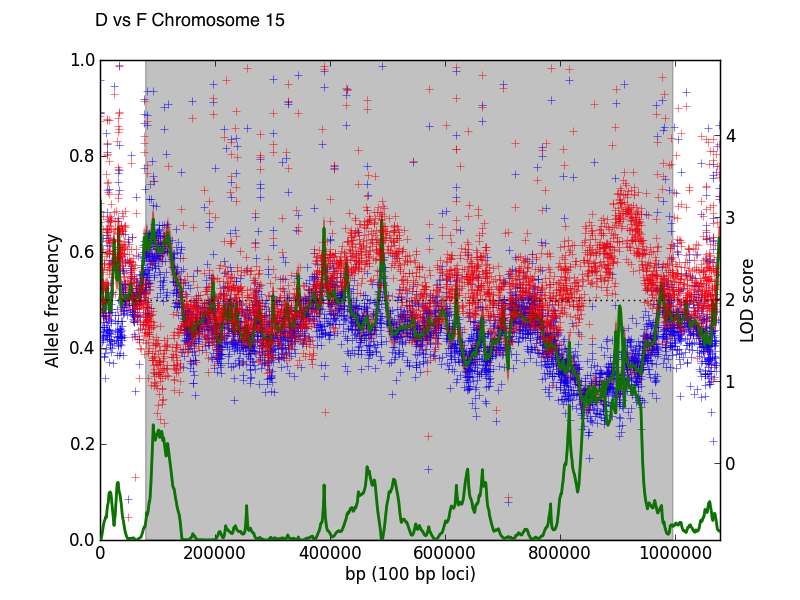

Supplement: Figure 1—source data 1. [file elife-52063-fig1-data1.zip › DvF/DvF_n46_ch15.png]

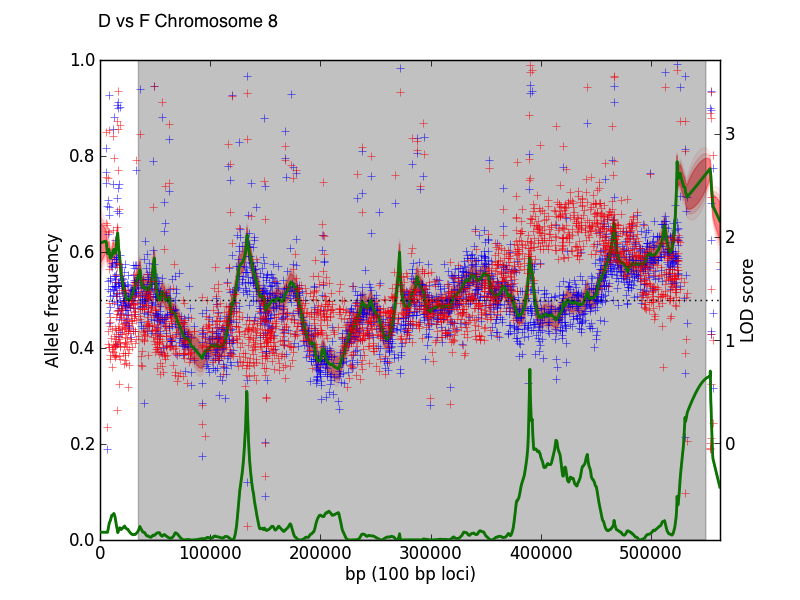

Supplement: Figure 1—source data 1. [file elife-52063-fig1-data1.zip › DvF/DvF_n46_ch8.png]

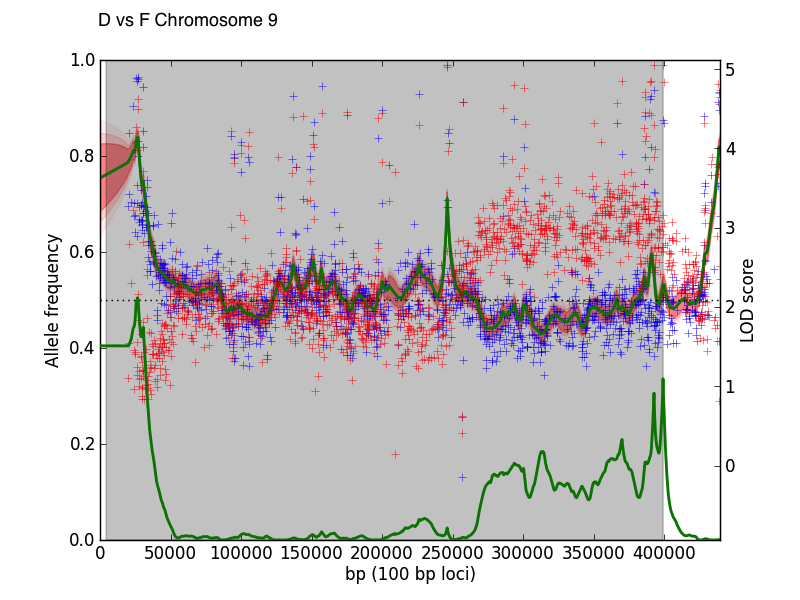

Supplement: Figure 1—source data 1. [file elife-52063-fig1-data1.zip › DvF/DvF_n46_ch9.png]
