# Supplementary material for: The genetic basis of aneuploidy tolerance in wild yeast
Source: eLife. 2020 Jan 7;9:e52063. doi: 10.7554/eLife.52063 (PMC6970514; doi:10.7554/eLife.52063)
Supplement: Figure 6—source data 1. — Fasta files were recapitulated by mapping SNPs from the published vcf file8 onto the S288c sequence and performing a multiple alignment using Clustal Omega (https://www.ebi.ac.uk/Tools/msa/clustalo/). Allelic differences are highlighted in yellow below. Only regions with polymorphisms are shown. [file elife-52063-fig6-data1.pdf]

Clustal Omega alignment of YPS1009 and seven other strains with truncated alleles.

Fasta files were recapitulated by mapping SNPs from the published vcf file () onto the S288c sequence and performing a multiple alignment using Clustal Omega (https://www.ebi.ac.uk/Tools/msa/clustalo/). Allelic differences are highlighted in yellow below. Only the YPS1009 sequence is shown beyond amino acid 840.

```
CH02_IvoryCoast_AVN:YDR293C      MSKNSNVNNNRSQEPNNMFVQTTGGGKNAPKQIHVAHRRSQSELTNLMIEQFTLQKQLEQ 60
CH13_IvoryCoast_AVT:YDR293C      MSKNSNVNNNRSQEPNNMFVQTTGGGKNAPKQIHVAHRRSQSELTNLMIEQFTLQKQLEQ 60
YPS1009_ADF:YDR293C              MSKNSNVNNNRFQEPNNMFVQTTGGGKNAPKQIHVAHRRSQSELTNLMIEQFTLQKQLEQ 60
CBS6308_AleBeer                  MSKNSNVNNNRSQEPNNMFVQTTGGGKNAPKQIHVAHRRSQSELTNLMIEQFTLQKQLEQ 60
CBK_Insect_Mosaic:YDR293C        MSKNSNVNNNRSQEPNNMFVQTTGGGKNAPKQIHVAHRRSQSELTNLMIEQFTLQKQLEQ 60
W303                             MSKNSNVNNNRSQEPNNMFVQTTGGGKNAPKQIHVAHRRSQSELTNLMIEQFTLQKQLEQ 60
CBL_InsectMosaic:YDR293C         MSKNSNVNNNRSQEPNNMFVQTTGGGKNAPKQIHVAHRRSQSELTNLMIEQFTLQKQLEQ 60
CBM_InsectMosaic:YDR293C         MSKNSNVNNNRSQEPNNMFVQTTGGGKNAPKQIHVAHRRSQSELTNLMIEQFTLQKQLEQ 60
                                 *********** ************************************************

CH02_IvoryCoast_AVN:YDR293C      VQAQQRQLMAQQQQLAQQTGQYLSGNSGSNNHFTPQPPHPHYNSNGNSPGMSAGGSRSRT 120
CH13_IvoryCoast_AVT:YDR293C      VQAQQRQLMAQQQQLAQQTGQYLSGNSGSNNHFTPQPPHPHYNSNGNSPGMSAGGSRSRT 120
YPS1009_ADF:YDR293C              VQAQQQQLMAQQQQLAQQTGQYLSGNSGSNNHFTPQPPHPHYNSNGNSPGMSAGGSRSRT 120
CBS6308_AleBeer                  VQAQQQQLMAQQQQLAQQTGQYLSGNSGSNNHFTPQPPHPHYNSNGNSPGMSAGGSRSRT 120
CBK_Insect_Mosaic:YDR293C        VQAQQQQLMAQQQQLAQQTGQYLSGNSGSNNHFTPQPPHPHYNSNGNSPGMSAGGSRSRT 120
W303                             VQAQQQQLMAQQQQLAQQTGQYLSGNSGSNNHFTPQPPHPHYNSNGNSPGMSAGGSRSRT 120
CBL_InsectMosaic:YDR293C         VQAQQQQLMAQQQQLAQQTGQYLSGNSGSNNHFTPQPPHPHYNSNGNSPGMSAGGSRSRT 120
CBM_InsectMosaic:YDR293C         VQAQQQQLMAQQQQLAQQTGQYLSGNSGSNNHFTPQPPHPHYNSNGNSPGMSAGGSRSRT 120
                                 *****:******************************************************

CH02_IvoryCoast_AVN:YDR293C      HSRNNSGYYHNSYDNNNNSNNPGSNSHRKTTSQSSIYGHSRRHSLGLNEAKKAAAEEQAK 180
CH13_IvoryCoast_AVT:YDR293C      HSRNNSGYYHNSYDNNNNSNNPGSNSHRKTSSQSSIYGHSRRHSLGLNEAKKAAAEEQAK 180
YPS1009_ADF:YDR293C              HSRNNSGYYHNSYDNNNNSNNPGSNSHRKTSSQSSIYGHSRRHSLGLNEAKKAAAEEQAK 180
CBS6308_AleBeer                  HSRNNSGYYHNSYDNNNNSNNPGSNSHRKTSSQSSIYGHSRRHSLGLNEAKKAAAEEQAK 180
CBK_Insect_Mosaic:YDR293C        HSRNNSGYYHNSYDNNNNSNNPGSNSHRKTSSQSSIYGHSRRHSLGLNEAKKAAAEEQAK 180
W303                             HSRNNSGYYHNSYDNNNNSNNPGSNSHRKTSSQSSIYGHSRRHSLGLNEAKKAAAEEQAK 180
CBL_InsectMosaic:YDR293C         HSRNNSGYYHNSYDNNNNSNNPGSNSHRKTSSQSSIYGHSRRHSLGLNEAKKAAAEEQAK 180
CBM_InsectMosaic:YDR293C         HSRNNSGYYHNSYDNNNNSNNPGSNSHRKTSSQSSIYGHSRRHSLGLNEAKKAAAEEQAK 180
                                 *****************************:******************************

CH02_IvoryCoast_AVN:YDR293C      RISGVKQA*--------------------------------------------------- 188
CH13_IvoryCoast_AVT:YDR293C      RISGGEAGVTVKMDSVQADSGSSSTTEQSDFKFPPPPNAHQGHRRATSNLSPPSFKFPPN 240
YPS1009_ADF:YDR293C              RISGGEAGVTVKIDSVQADSGSNFTTEQSDFKFPPPPNAHQGHRRATSNLSPPSFKFPPN 240
CBS6308_AleBeer                  RISGGEAGVTVKIDSVQADSGSNSTTEQSDFKFPPPPNAHQGHRRATSNLSPPSFKFPPN 240
CBK_Insect_Mosaic:YDR293C        RISGGEAGVTVKIDSVQADSGSNSTTEQSDFKFPPPPNAHQGHRRATSNLSPPSFKFPPN 240
W303                             RISGGEAGVTVKIDSVQADSGSNSTTEQSDFKFPPPPNAHQGHRRATSNLSPPSFKFPPN 240
CBL_InsectMosaic:YDR293C         RISGGEAGVTVKIDSVQADSGSNSTTEQSDFKFPPPPNAHQGHRRATSNLSPPSFKFPPN 240
CBM_InsectMosaic:YDR293C         RISGGEAGVTVKIDSVQADSGSNSTTEQSDFKFPPPPNAHQGHRRATSNLSPPSFKFPPN 240
                                 **** : .

CH02_IvoryCoast_AVN:YDR293C      ------------------------------------------------------------ 188
CH13_IvoryCoast_AVT:YDR293C      SHGDNDDEFIATSSTHRRSKTRNNEYSPGINSNWRNQSQQPQQQLSPFRHRGSNSRDYNS 300
YPS1009_ADF:YDR293C              SHGDNDDEFIATSSTHRRSKTRNNEYSPGINSNWRNQSQQPQQQLSPFRHRGSNSRDYNS 300
CBS6308_AleBeer                  SHGDNDDEFIATSSTHRRSKTRNNEYSPGINSNWRNQSQQPQQQLSPFRHRGSNSRDYNS 300
CBK_Insect_Mosaic:YDR293C        SHGDNDDEFIATSSTHRRSKTRNNEYSPGINSNWRNQSQQPQQQLSPFRHRGSNSRDYNS 300
W303                             SHGDNDDEFIATSSTHRRSKTRNNEYSPGINSNWRNQSQQPQQQLSPFRHRGSNSRDYNS 300
CBL_InsectMosaic:YDR293C         SHGDNDDEFIATSSTHRRSKTRNNEYSPGINSNWRNQSQQPQQQLSPFRHRGSNSRDYNS 300
CBM_InsectMosaic:YDR293C         SHGDNDDEFIATSSTHRRSKTRNNEYSPGINSNWRNQSQQPQQQLSPFRHRGSNSRDYNS 300

CH02_IvoryCoast_AVN:YDR293C      ------------------------------------------------------------ 188
CH13_IvoryCoast_AVT:YDR293C      FNTLEPPAIFQQGHKHRASNSSVHSFSSQGNNNGGGRKSLFAPYLPQANIPELIQEGRLV 360
YPS1009_ADF:YDR293C              FNTLEPPAIFQQGHKHRASNSSVHSFSSQGNNNGGGRKSLFAPYLPQANIPELIQEGRLV 360
CBS6308_AleBeer                  FNTLEPPAIFQQGHKHRASNSSVHSFSSQGNNNGGGRKSLFAPYLPQANIPELIQEGRLV 360
CBK_Insect_Mosaic:YDR293C        FNTLEPPAIFQQGHKHRASNSSVHSFSSQGNNNGGGRKSLFAPYLPQANIPELIQEGRLV 360
W303                             FNTLEPPAIFQQGHKHRASNSSVHSFSSQGNNNGGGRKSLFAPYLPQANIPELIQEGRLV 360
```

```
CBL_InsectMosaic:YDR293C        FNTLEPPAIFQQGHKHRASNSSVHSFSSQGNNNGGGRKSLFAPYLPQANIPELIQEGRLV 360
CBM_InsectMosaic:YDR293C        FNTLEPPAIFQQGHKHRASNSSVHSFSSQGNNNGGGRKSLFAPYLPQANIPELIQEGRLV 360

CH02_IvoryCoast_AVN:YDR293C     ------------------------------------------------------------ 188
CH13_IvoryCoast_AVT:YDR293C     AGILRVNKKNRSDAWVSTDGALDADIYICGSKDRNRALEGDLVAVELLVVDDVWESKKEK 420
YPS1009_ADF:YDR293C             AGILRVNKKNRSDAWVSTDGALDADIYICGSKDRNRALEGDLVAVELLVVDDVWESKKEK 420
CBS6308_AleBeer                 AGILRVNKKNRSDAWVSTDGALDADIYICGSKDRNRALEGDLVAVELLVVDDVWESKKEK 420
CBK_Insect_Mosaic:YDR293C       AGILRVNKKNRSDAWVSTDGALDADIYICGSKDRNRALEGDLVAVELLVVDDVWESKKEK 420
W303                            AGILRVNKKNRSDAWVSTDGALDADIYICGSKDRNRALEGDLVAVELLVVDDVWESKKEK 420
CBL_InsectMosaic:YDR293C        AGILRVNKKNRSDAWVCTDGALDADIYICGSKDRNRALEGDLVAVELLVVDDVWESKKEK 420
CBM_InsectMosaic:YDR293C        AGILRVNKKNRSDAWVCTDGALDADIYICGSKDRNRALEGDLVAVELLVVDDVWESKKEK 420

CH02_IvoryCoast_AVN:YDR293C     ------------------------------------------------------------ 188
CH13_IvoryCoast_AVT:YDR293C     EEKKRRKDASMQHDLIP*------------------------------------------ 437
YPS1009_ADF:YDR293C             EEKKRRKDASMQHDLIPLNSSDDYHNDASVTAATSNNFLSSPSSSDSLSKDDLSVRRKRS 480
CBS6308_AleBeer                 EEKKRRKDASMQHDLIPFNSSDDYHNDASVTAATSNNFLSSPSSSDSLSKDDLSVRRKRS 480
CBK_Insect_Mosaic:YDR293C       EEKKRRKDASMQHDLIPLNSSDDYHNDASVTAATSNNFLSSPSSSDSLSKDDLSVRRKRS 480
W303                            EEKKRRKDASMQHDLIPLNSSDDYHNDASVTAATSNNFLSSPSSSDSLSKDDLSVRRKRS 480
CBL_InsectMosaic:YDR293C        EEKKRRKDASMQHDLIPLNSSDDYHNDASVTAATSNNFLSSPSSSDSLSKDDLSVRRKRS 480
CBM_InsectMosaic:YDR293C        EEKKRRKDASMQHDLIPLNSSDDYHNDASVTAATSNNFLSSPSSSDSLSKDDLSVRRKRS 480

CH02_IvoryCoast_AVN:YDR293C     ------------------------------------------------------------ 188
CH13_IvoryCoast_AVT:YDR293C     ------------------------------------------------------------ 437
YPS1009_ADF:YDR293C             STINNDSDSLSSPTKSGVRRRSSLKQRPTQKKNDDVEVEGQSLLLVEEEEINDKYKPLYA 540
CBS6308_AleBeer                 STINNDSDSLSSPTKSGVRRRSSLKQRPTQKKNDDVEVEGQSLLLVEEEEINDKYKPLYA 540
CBK_Insect_Mosaic:YDR293C       STINNDSDSLSSPTKSGVRRRSSLKQRPTQKKNDDVEVEGQSLLLVEEEEINDKYKPLYA 540
W303                            STINNDSDSLSSPTKSGVRRRSSLKQRPTQKKNDDVEVEGQSLLLVEEEEINDKYKPLYA 540
CBL_InsectMosaic:YDR293C        STINNDSDSLSSPTKSGVRRRSSLKQRPTQKKNDDVEVEGQSLLLVEEEEINDKYKPLYA 540
CBM_InsectMosaic:YDR293C        STINNDSDSLSSPTKSGVRRRSSLKQRPTQKKNDDVEVEGQSLLLVEEEEINDKYKPLYA 540

CH02_IvoryCoast_AVN:YDR293C     ------------------------------------------------------------ 188
CH13_IvoryCoast_AVT:YDR293C     ------------------------------------------------------------ 437
YPS1009_ADF:YDR293C             GHVVAVLDRIPGQLFSGTLGLLRPSQQANSDNNKPPQSPKIAWFKPTDKKVPLIAIPTEL 600
CBS6308_AleBeer                 GHVVAVLDRIPGQLFSGTLGLLRPSQQANSDNNKPPQSPKIAWFKPTDKKVPLIAIPTEL 600
CBK_Insect_Mosaic:YDR293C       GHVVAVLDRIPGQLFSGTLGLLRPSQQANSDNNKPPQSPKIAWFKPTDKKVPLIAIPTEL 600
W303                            GHVVAVLDRIPGQLFSGTLGLLRPSQQANSDNNKPPQSPKIAWFKPTDKKVPLIAIPTEL 600
CBL_InsectMosaic:YDR293C        GHVVAVLDRIPGQLFSGTLGLLRPSQQANSDNNKPPQSPKIAWFKPTDKKVPLIAIPTEL 600
CBM_InsectMosaic:YDR293C        GHVVAVLDRIPGQLFSGTLGLLRPSQQANSDNNKPPQSPKIAWFKPTDKKVPLIAIPTEL 600

CH02_IvoryCoast_AVN:YDR293C     ------------------------------------------------------------ 188
CH13_IvoryCoast_AVT:YDR293C     ------------------------------------------------------------ 437
YPS1009_ADF:YDR293C             APKDFVENADKYSEKLFVASIKRWPITSLHPFGILVSELGDIHDPDTEIDSILRDNNFLS 660
CBS6308_AleBeer                 APKDFVENADKYSEKLFVASIKRWPITSLHPFGILVSELGDIHDPDTEIDFILRDNNFLS 660
CBK_Insect_Mosaic:YDR293C       APKDFVENADKYSEKLFVASIKRWPITSLHPFGILVSELGDIHDPDTEIDSILRDNNFLS 660
W303                            APKDFVENADKYSEKLFVASIKRWPITSLHPFGILVSELGDIHDPDTEIDSILRDNNFLS 660
CBL_InsectMosaic:YDR293C        APKDFVENADKYSEKLFVASIKRWPITSLHPFGILVSELGDIHDPDTEIDSILRDNNFLS 660
CBM_InsectMosaic:YDR293C        APKDFVENADKYSEKLFVASIKRWPITSLHPFGILVSELGDIHDPDTEIDSILRDNNFLS 660

CH02_IvoryCoast_AVN:YDR293C     ------------------------------------------------------------ 188
CH13_IvoryCoast_AVT:YDR293C     ------------------------------------------------------------ 437
YPS1009_ADF:YDR293C             NEYLDQKNPQKEKPSFQPLPLTAESLEYRRNFMDTNEYNIFAISELGWVSEFALHVRNNG 720
CBS6308_AleBeer                 NEYLDQKNPQKEKPSFQPLPLTAESLEYRRNFTDTNEYNIFAISELGWVSEFALHVRNNG 720
CBK_Insect_Mosaic:YDR293C       NEYLDQKNPQKEKPSFQPLPLTAESLEYRRNFTDTNE*---------------------- 697
W303                            NEYLDQKNPQKEKPSFQPLPLTAESLEYRRNFTDTNE*---------------------- 697
CBL_InsectMosaic:YDR293C        NEYLDQKNPQKEKPSFQPLPLTAESLEYRRNFMDTNE*---------------------- 697
CBM_InsectMosaic:YDR293C        NEYLDQKNPQKEKPSFQPLPLTAESLEYRRNFMDTNE*---------------------- 697

CH02_IvoryCoast_AVN:YDR293C     ------------------------------------------------------------ 188
```

```
CH13_IvoryCoast_AVT:YDR293C      ------------------------------------------------------------ 437
YPS1009_ADF:YDR293C              NGTLELGCHVVDVTSHIEEGSSVDRRARKRSSAVFMPQKLVNLLPQSFNDELSLAPGKES 780
CBS6308_AleBeer                  NGTLELGCHVVDVTSHIEEGSSVDRRARKRSSAVFMPQKLVNLLPQSFNDELSLALARNQ 780
CBK_Insect_Mosaic:YDR293C        ------------------------------------------------------------ 697
W303                             ------------------------------------------------------------ 697
CBL_InsectMosaic:YDR293C         ------------------------------------------------------------ 697
CBM_InsectMosaic:YDR293C         ------------------------------------------------------------ 697

CH02_IvoryCoast_AVN:YDR293C      ------------------------------------------------------------ 188
CH13_IvoryCoast_AVT:YDR293C      ------------------------------------------------------------ 437
YPS1009_ADF:YDR293C              ATLSVVYTLDSSTLRIKSTWVGESTISPSNILSLEQLDEKLSTGSPSSYLSTVQEIARSF 840
CBS6308_AleBeer                  PRCRL-FTL*-------------------------------------------------- 788

YPS1009_ADF:YDR293C              YARRINDPEATLLPTLSLLESLDDEKVKVDLNILDRTLGFVVINEIKRKVNSTVAEKIYT 900
YPS1009_ADF:YDR293C              KLGDLALLRRQMQPIATKMASFRKKIQNFGYNFDTNTADELIKGVLKIKDDDVRVGIEIL 960
YPS1009_ADF:YDR293C              LFKTMPRARYFIAGKVDPDQYGHYALNLPIYTHFTAPMRRYADHVVHRQLKAVIHDTPYT 1020
YPS1009_ADF:YDR293C              EDMEALKITSEYCNFKKDCAYQAQEQAIHLLLCKTINDMGNTTGQLLTMATVLQVYESSF 1080
YPS1009_ADF:YDR293C              DVFIPEFGIEKRVHGDQLPLIKAEFDGTNRVLELHWQPGVDSATFIPADEKNPKSYRNSI 1140
YPS1009_ADF:YDR293C              KNKFRSTAAEIANIELDKEAESEPLISDPLSKELSDLHLTVPNLRLPSAGDNKQNPLEKF 1200
YPS1009_ADF:YDR293C              ISTTETRIENDNYIQEIHELQKIPILLRAEVGMALPCLTVRALNPFMKRV* 1250
```
